# Co-operation, Competition and Crowding: A Discrete Framework Linking Allee Kinetics, Nonlinear Diffusion, Shocks and Sharp-Fronted Travelling Waves

**DOI:** 10.1038/srep42134

**Published:** 2017-02-14

**Authors:** Stuart T. Johnston, Ruth E. Baker, D. L. Sean McElwain, Matthew J. Simpson

**Affiliations:** 1 Mathematical Sciences, Queensland University of Technology (QUT), Brisbane, Australia; 2 Tissue Repair and Regeneration Program, Institute of Health and Biomedical Innovation, QUT, Brisbane, Australia; 3 Wolfson Centre for Mathematical Biology, Mathematical Institute, University of Oxford, United Kingdom

## Abstract

Invasion processes are ubiquitous throughout cell biology and ecology. During invasion, individuals can become isolated from the bulk population and behave differently. We present a discrete, exclusion-based description of the birth, death and movement of individuals. The model distinguishes between individuals that are part of, or are isolated from, the bulk population by imposing different rates of birth, death and movement. This enables the simulation of various co-operative or competitive mechanisms, where there is either a positive or negative benefit associated with being part of the bulk population, respectively. The mean-field approximation of the discrete process gives rise to 22 different classes of partial differential equation, which can include Allee kinetics and nonlinear diffusion. Here we examine the ability of each class of partial differential equation to support travelling wave solutions and interpret the long time behaviour in terms of the individual-level parameters. For the first time we show that the strong Allee effect and nonlinear diffusion can result in shock-fronted travelling waves. We also demonstrate how differences in group and individual motility rates can influence the persistence of a population and provide conditions for the successful invasion of a population.

Processes where individuals invade, and subsequently colonise, a region of space are prevalent in cell biology and ecology^1^,^2^,^3^,^4^,^5^,^6^,^7^,^8^,^9^,^10^,^11^. In cell biology, wound healing involves the invasion of fibroblasts into the wound space for tissue regeneration^7^. The invasion of glioma cells throughout the brain can lead to the formation of malignant brain tumours (glioblastoma)^1^,^3^,^10^. In ecology, the introduction and subsequent invasion of an alien species is a significant factor contributing to the extinction of native species^2^,^11^.

During invasion, individuals that become separated from the bulk population have been observed to have different behaviours to individuals within the bulk population^8^,^12^,^13^,^14^,^15^. This is intuitive in ecological processes, as a decrease in the number of individuals within the bulk population can reduce the number of potential mates^13^,^15^,^16^,^17^ or lessen the efficacy of predator avoidance^14^,^15^,^16^. In cell biology, individual micrometastases have been observed to have reduced growth rates below a threshold size, which suggests that the presence of additional cells enhances the birth rate^12^.

Continuum mathematical models of invasion processes have been studied extensively since the Fisher-Kolmogorov model was first proposed in 1937^15^,^18^,^19^,^20^,^21^,^22^,^23^,^24^,^25^,^26^,^27^,^28^,^29^,^30^. The Fisher-Kolmogorov model is a partial differential equation (PDE) description of the evolution of population density, where the temporal change in population density is attributed to a combination of linear diffusion and logistic growth^24^,^27^. The Fisher-Kolmogorov model has been applied to various problems in cell biology and ecology^31^,^32^,^33^,^34^. The logistic growth term implies that the population density will always tend toward the carrying capacity^29^. This prediction does not reflect the observation that isolated individuals can experience a reduction in their birth rate^14^. This effect, known as the Allee effect, has two known forms. First, the strong Allee effect, where the growth rate is negative for sufficiently low densities^15^,^35^. Second, the weak Allee effect, where the growth rate is reduced, but remains positive, at low densities^15^. Reaction-diffusion PDEs incorporating linear diffusion and Allee growth kinetics have been proposed and analysed^15^,^19^,^22^,^23^,^25^,^26^,^28^,^30^. A key feature of interest for models of invasion is whether the PDE supports a travelling wave solution, where a wave front of constant shape moves through space with a constant speed. The sign of the wave speed indicates whether successful invasion occurs, and the magnitude of the wave speed provides an estimate of how quickly a population invades or recedes. More complicated descriptions of invasion processes with either Fisher or Allee kinetics and density-dependent nonlinear diffusion have been proposed, with the motivation of describing spatial aggregation or segregation^36^,^37^,^38^,^39^,^40^,^41^.

A key feature of the Fisher-Kolmogorov model, and many extensions thereof, is that these PDE models are typically derived using phenomenologically-based arguments without incorporating information from an underlying stochastic description of individual-level behaviours. In this work we consider a relatively straightforward lattice-based discrete birth-death-movement model. An important characteristic of the model is that it explicitly accounts for crowding effects by only allowing for one agent per lattice site. Additionally, the rates at which birth, death and movement events occur depend on whether an agent is part of a group of agents or is isolated. We demonstrate that the standard continuum approximation of this discrete model can lead to either logistic or Allee kinetics, in an appropriate parameter regime. Furthermore, we demonstrate that imposing a different motility rate for agents that are isolated, compared to other agents, leads to a variety of density-dependent nonlinear diffusion functions. Previous studies have examined many different types of phenomenologically-based PDEs that are motivated in an *ad hoc* fashion. In contrast, our PDE description arises from a single, relatively simple, physically-motivated model. In Table 1 we highlight this generality, as the single discrete model gives rise to 22 different classes of PDE that describe the population-level behaviour.

While several of these PDEs have been studied previously, for completeness we examine the ability of each class of PDE to support travelling wave solutions. For certain classes of PDE, we present details of the travelling wave solutions for the first time. Interestingly, we obtain travelling wave solutions for PDEs that have nonlinear diffusivity functions with regions of negative diffusivity. Furthermore, we show that the strong Allee effect combined with these diffusivity functions can lead to novel shock-fronted travelling wave solutions. As these diffusivity functions are obtained directly from a discrete model, we can determine which competitive/co-operative individual-level mechanisms result in shock-fronted travelling wave solutions. Similarly, we are able to interpret the influence of motility on the persistence of a population, and highlight how this influence varies nonlinearly with the carrying capacity density. More generally, we provide new insight into the long time behaviour of an invasive population in terms of its individual-level properties.

## Results

We consider a discrete lattice-based exclusion process where agents undergo birth, death and movement events. We distinguish between isolated agents and grouped agents by imposing different rates of birth, death and movement depending on whether an agent has zero or at least one nearest-neighbour agent, respectively. A more detailed description of the discrete model is presented in the Methods. To derive a continuum limit PDE description of the discrete model^42^,^43^ we consider the change in occupancy of a lattice site *j* during a single time step of duration *τ*, and obtain


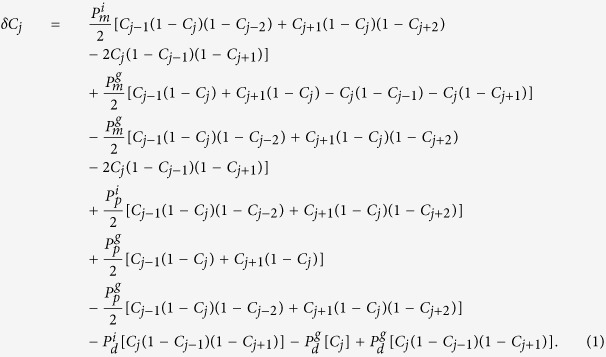


Here, *C*_*j*_ represents the probability that the site *j* is occupied and, therefore, 1 − *C*_*j*_ represents the probability that the site is vacant^43^. Furthermore, as products of probabilities are interpreted as net transition probabilities, the usual assumption that the occupancy of lattice sites are independent is made^42^,^44^,^45^,^46^,^47^.

Note that *C*_*j*_ is the total occupancy of site *j*, that is, the sum of the occupancy of isolated agents and the occupancy of grouped agents at that site. We now interpret the terms on the right-hand side of Equation (1) in terms of the physical change in lattice occupancy. The positive terms proportional to 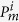 correspond to isolated agents moving into site *j*, while the negative terms correspond to isolated agents moving out of site *j*. Each term consists of three factors. For the negative terms, these factors are the probability that site *j* is occupied, and the probabilities that sites *j* − 1 and *j* + 1 are vacant. For the positive terms, the three factors are the probability that site *j* ± 1 is occupied, and the probabilities that sites *j* and *j* ± 2 are vacant. The third factor is required to ensure that the term describes isolated agents. The positive/negative terms proportional to the first 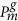 term on the right-hand side of Equation (1) correspond to grouped agents moving in/out of site *j*. These terms consist of two factors; the probability that the selected site is occupied and the probability that the target site is vacant. The second 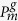 term ensures that the isolated agents are not counted twice. The remaining terms can be interpreted similarly; products of probabilities that specific sites are occupied or vacant that describe the change of occupancy of a site in response to a birth or death event.

To obtain a PDE description we divide Equation (1) by *τ* and consider *C*_*j*_ as a continuous function, *C(x, t*). We expand *C(x, t*) in a Taylor series around *x* = *j*Δ, truncating terms of 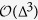, where Δ is the lattice spacing^42^,^43^. Taking the limit Δ → 0 and *τ* → 0 such that Δ^2^/*τ* is held constant^43^,^48^,^49^ gives


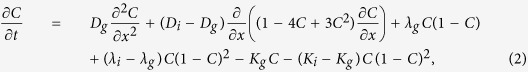


where


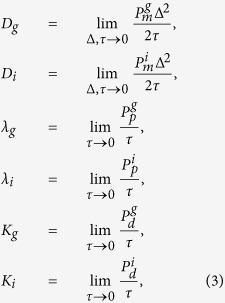


with the further assumption that 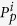, 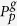, 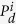, 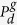 are 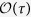
43. The individual-level parameters are treated as being interchangeable with the continuum-level parameters as defined in (3). All implementations of the discrete model in this work have Δ = *τ* = 1.

It is convenient to write Equation (2) in conservation form


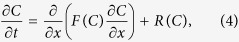


where


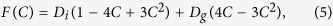


is the nonlinear diffusivity function, and


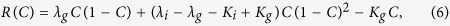


is the source/sink term.

The aims of this work are to first illustrate that the very different types of behaviour encoded in the discrete model are also reflected in the solution of Equation (2). Once we have demonstrated this connection, we focus on examining travelling wave solutions for the 22 different classes embedded within Equation (2), as summarised in Table 1. In the main document we highlight novel and key results for specific classes of PDEs resulting from the discrete model, and provide relevant discussion about the implications of the long time population behaviour. A more thorough investigation of the travelling wave solutions arising from all 22 classes of PDEs is presented in the Supplementary Material.

**Figure 1:**
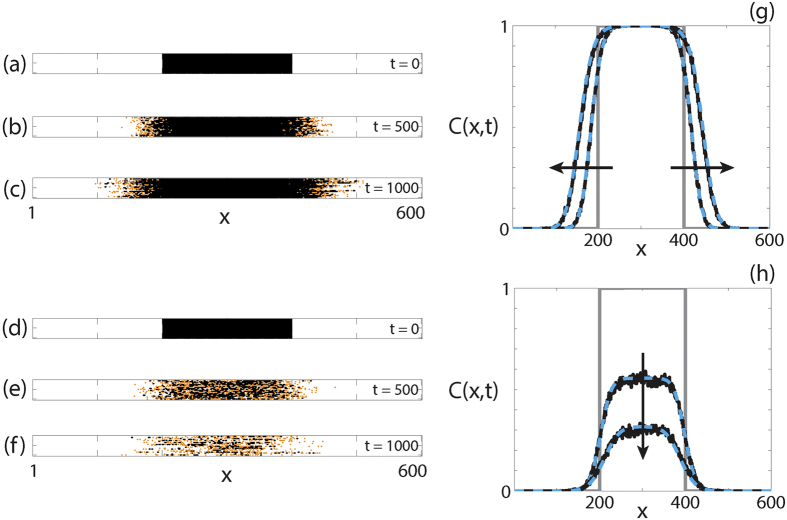
Comparison of the discrete model and the continuum approximation. (**a**–**f**) 20 identically-prepared realisations of the discrete model at (**a**,**d**) *t* = 0; (**b**,**e**) *t* = 500; (**c**,**f**) *t* = 1000. The discrete model simulations correspond to (**a**–**c**) 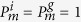, 
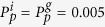
, 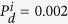, 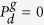; (**d**–**f**) 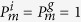, 
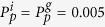
, 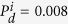, 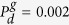. For all simulations *τ* = 1, Δ = 1. (**g**,**h**) Comparisons between the averaged discrete model (black, solid) and the numerical solution of Equation (2) (cyan, dashed) at *t* = 0, *t* = 500 and *t* = 1000 for the parameters in (**a**–**f**), respectively. The grey lines indicate the initial condition and the arrow indicates the direction of increasing time. For all discrete solutions, *M* = 1000, *X* = 600, Δ = *τ* = 1. For all continuum solutions, *δx* = 1, *δt* = 0.1, *ε* = 10^−6^.

Twenty identically-prepared realisations of the discrete model are presented in Fig. 1(a–f) for two different parameter regimes. In the first parameter regime, where 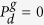, the initially-occupied region of the lattice remains fully occupied, as shown in Fig. 1(a–c). When we introduce 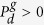, as shown in Fig. 1(d–f), the initially-occupied region of the lattice becomes partially vacant as time increases. We also compare the average discrete behaviour and the corresponding numerical solution of Equation (2) in Fig. 1(g,h). This comparison shows that the solution of the continuum PDE matches the average discrete behaviour well, and predicts both the spread of the agent population in Fig. 1(g) and the decrease in agent density in Fig. 1(h).

The governing PDE, Equation (2), can be simplified in specific parameter regimes. While several of these simplified PDEs have been studied extensively, we summarise all non-trivial cases for completeness. It is instructive to consider each case and discuss the implications of the long term behaviour in terms of the discrete model parameters, as previous derivations of these PDEs have arisen from a variety of *ad hoc* arguments rather than working with a single unifying model. In Table 1 we summarise the salient features of 22 different special cases of Equation (2). The nonlinear diffusivity function, *F(C*), has four key properties:

- *F(C*) can either be a constant, or a function of the density of individuals;
- *F(C*) can be degenerate, which implies that at one or more densities, C*, we have F(C*) = R(C*) = 0;
- *F(C*) can be zero at values of C* that are non-degenerate, that is, F(C*) = 0, 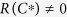. In our model, this can occur at either zero, one or two different values of *C*; and
- *F(C*) can be negative for an interval of *C* values.
- The source term, *R(C*), has two key properties:
- *R(C*) can represent either Fisher kinetics (logistic growth) or Allee kinetics (bistable); and
- The grouped agent death rate, 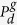, can be zero or non-zero. If the rate is non-zero, the carrying capacity density is reduced.

There are three different types of Allee kinetics considered in this work; weak, strong and reverse. We consider these three kinetics together for brevity, as it is relatively simple to change the parameter regime to alter the type of Allee effect without changing the competitive/co-operative mechanism described. The reverse Allee effect, which we describe here for the first time, refers to a growth rate that is reduced at high density, compared to logistic kinetics, but remains positive.

### Fisher kinetics

The choice of whether the birth and death mechanisms imposed in the discrete model are neutral or are competitive/co-operative determines the form of the source term. If both 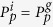 and 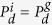, and hence both the birth and death mechanisms are neutral, the source term represents Fisher kinetics and Equation (2) simplifies to


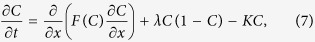


where *λ* = *λ*_*i*_ = *λ*_*g*_ and *K* = *K*_*i*_ = *K*_*g*_. Transforming Equation (7) into travelling wave co-ordinates *z* = *x* − *vt*, where *v* is a constant wave speed and −∞ < *z* < ∞, results in


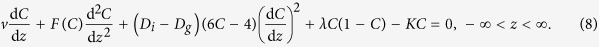


Substituting *U* = d*C*/d*z* allows Equation (8) to be expressed as a system of ordinary differential equations (ODEs)


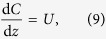



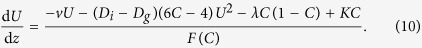


The equilibrium points of Equations (9 and 10) occur at (*C, U*) = (0, 0) and (*C, U*) = (*S*, 0), where *S* = (*λ* − *K*)/*λ*. The range of physically relevant *C* values correspond to 0 ≤ *C* ≤ *S*. Hence the carrying capacity density, *S*, determines the numbers of times that *F(C*) = 0 for physically relevant *C* values. As such, we introduce a new variable 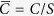 such that the agent density is scaled by the carrying capacity and the zeroes of 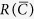 occur at 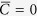 and 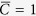.

**Figure 2:**
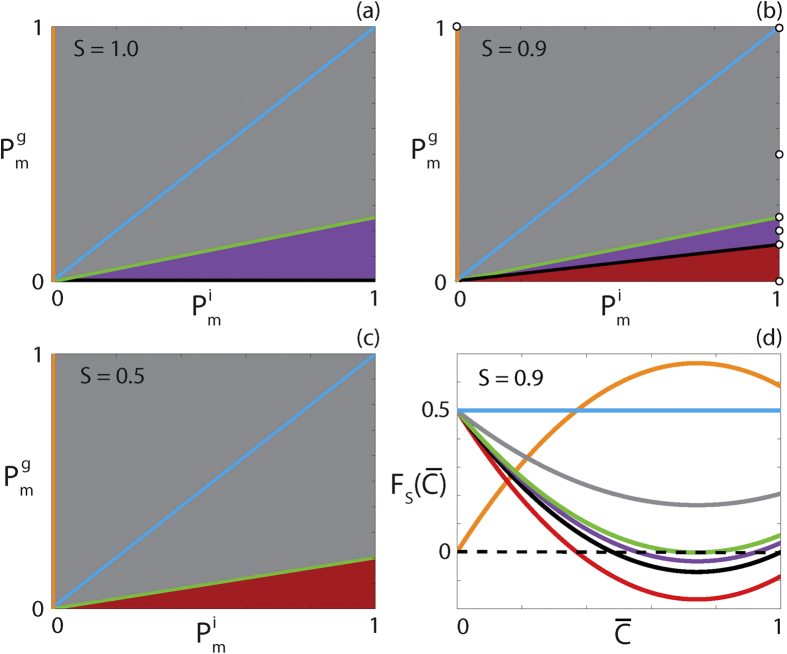
Classification of 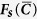 for different carrying capacity densities. (**a**–**c**) Type of 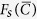 function for 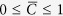 for the parameter space 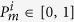 and 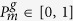 with (**a**) *S* = 1.0; (**b**) *S* = 0.9; (**c**) *S* = 0.5. Grey regions correspond to parameter pairs that result in strictly positive 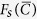, purple regions correspond to parameter pairs that result in positive-negative-positive 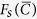 and red regions correspond to parameter pairs that result in positive-negative 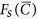. Cyan, orange and black lines correspond to constant, extinction-degenerate non-negative and capacity-degenerate positive-negative 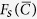 curves, respectively. (**d**) Example 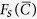 for each region in (**b**). The white circles in (**b**) denote the parameter pairs used to generate the curves in (**d**).

Transforming Equation (7) in terms of 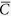, we obtain


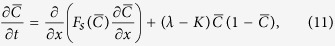


where 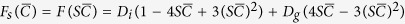. Equation (11) is the Fisher-Kolmogorov equation in terms of 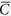 with a nonlinear diffusivity function, 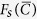. This new nonlinear diffusivity function has different properties depending on *S, D*_*i*_ and *D*_*g*_. To highlight this, Fig. 2 shows the 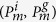 parameter space for three different choices of *S* and the qualitative behaviour of the corresponding 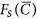 function. For all *S* values, parameter pairs that result in a constant 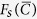 are highlighted in cyan. All parameter pairs that result in 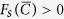 for 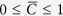 are denoted by the grey regions. This type of diffusivity function is referred to as *strictly positive*. Similarly, for all *S* values, there are parameter pairs that result in 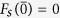, and 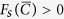 otherwise, which are highlighted in orange. We refer to this type of diffusivity function as *extinction*-*degenerate non*-*negative*.

For *S* = 1, presented in Fig. 2(a), 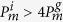, denoted in purple, results in an interval 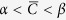, *α* < *β* < 1, where 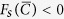. We refer to this type of nonlinear diffusivity function as *positive*-*negative*-*positive*. Decreasing *S* to 0.9, presented in Fig. 2(b), we observe that the purple region again occurs for 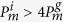. However, if 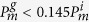, highlighted in red, 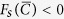 for 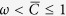, and hence 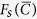 has only one zero in 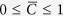. This type of nonlinear diffusivity function is not observed with *S* = 1 and we refer to it as *positive*-*negative*. Specifically, this behaviour occurs when 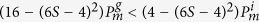 and 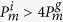. Furthermore, this implies that for *S* < 2/3 there are no 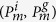 values that correspond to positive-negative-positive 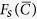. A choice of 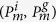 that demonstrates this is shown in Fig. 2(c). Unlike in Fig. 2(a,b), we see that there is no purple region. Finally, if 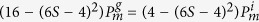, highlighted in black, *F*_*s*_(1) = 0 and 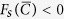 for 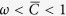, which we refer to as *capacity*-*degenerate positive*-*negative*. Note that for *S* < 1/3, 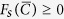 for 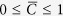. An example 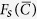 curve for each type of diffusivity function is presented in Fig. 2(d). PDE models that contain diffusivity functions with a region of negative diffusivity have been considered previously. However, these models either do not contain a source term or consider source terms that do not support travelling wave solutions^50^,^51^,^52^. Hence the model and analysis considered in this work is significantly different to the previous studies.

For all combinations of neutral, competitive and co-operative mechanisms that give rise to a reaction-diffusion equation with Fisher kinetics we examine the ability of the equation to give rise to long time travelling wave solutions. While details of the travelling wave solutions for certain types of diffusivity functions have been presented previously, we summarise the key features of the travelling wave solutions in tabular form for all cases for completeness in Table 2. For the cases where solution profiles have not been presented previously, we provide more detailed discussion. A detailed analysis for each case is presented in the Supplementary Material.

#### Positive-negative-positive nonlinear diffusivity function

The first diffusivity function we examine in detail is the positive-negative-positive nonlinear diffusivity function, where 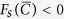 for an interval 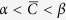. The simplest positive-negative-positive 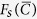 occurs where 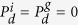 and hence *S* = 1. For these parameters, 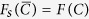. Note that introducing non-zero 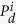 and 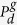 merely scales the governing equation and hence extending this analysis to cases with non-zero agent death is straightforward, provided that 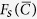 has two zeroes on the interval 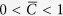. Parameters that result in a positive-negative-positive 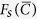 are highlighted in purple in Fig. 2 and, for this case, with 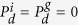, occur when 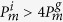. For positive-negative-positive *F(C*), Equation (10) is singular at *C* = *α* and *C* = *β*, where the interval of *F(C*) < 0 is given by


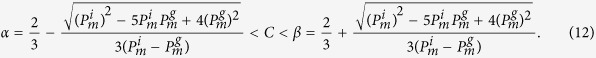


The singularities at *C* = *α* and *C* = *β* cannot be removed using a stretching transformation (Supplementary Material) since 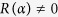 and 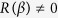. However, it is possible for d*U*/d*z* to be finite at *C* = *α* and *C* = *β* if *U*_*α*_ and *U*_*β*_ exist such that


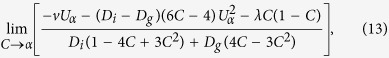



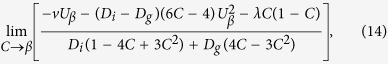


are both finite. This requires the numerator in the expressions (13 and 14) vanish at *C* = *α* and *C* = *β*, respectively. As such, *U*_*α*_ and *U*_*β*_ are obtained by solving the system


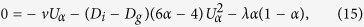



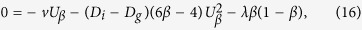


resulting in 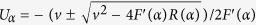 and 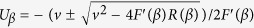. We note that as *R(C*) ≥ 0 for 0 ≤ *C* ≤ 1, and that *F*′(*α*) ≤ 0 for all possible *α* values, *U*_*α*_ will be real-valued. Subsequently, we have a wave speed condition that 
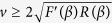
, as 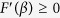 for all possible *β* values. Ferracuti *et al*.^36^ prove that the minimum wave speed, v*, is greater than a threshold value, which in turn is greater than max{*R*′(0) *F*(0), *F*′(*β*) *R(β*)}. Therefore, *U*_*β*_ will also always be real-valued.

Applying L’Hopital’s Rule to Equation (10), we obtain


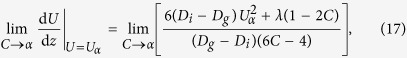



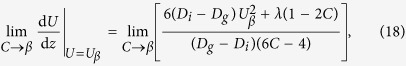


which are finite provided that 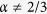 and 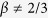. For the system of Equations (9 and 10), we have two straight lines in the phase plane where d*U*/d*z* is infinite, at *C* = *α* and *C* = *β*. These kind of lines have previously been called *walls of singularities* for hyperbolic models related to chemotactic and haptotactic invasion^53^. For a smooth solution trajectory joining the two equilibrium points on opposite sides of the wall of singularities, we require that the trajectory passes through the wall of singularities. This implies that the solution trajectory must pass through the wall of singularities at the special points, (*α, U*_*α*_) and (*β, U*_*β*_), known as *holes in the wall*^53^,^54^. Otherwise, a smooth heteroclinic orbit between (1, 0) and (0, 0) cannot exist, as 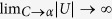 and 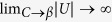. As *U*_*α*_ and *U*_*β*_ are real valued and the limits in Equations (11 and 12) are finite, the holes in the wall always exist for Fisher kinetics.

**Figure 3:**
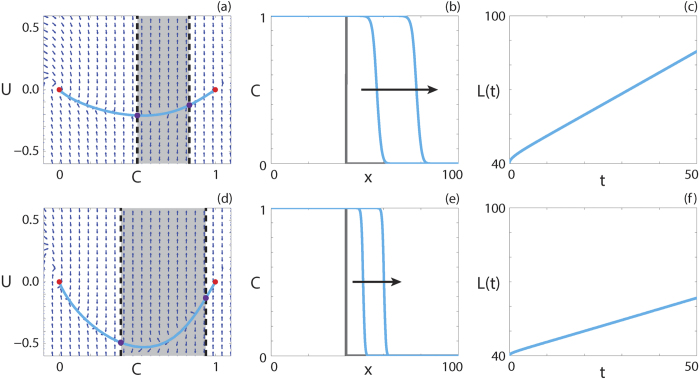
Travelling wave behaviour for Equation (7) with positive-negative-positive *F(C*) (Case 2.3). (**a**,**d**) Phase plane for the system (9 and 10) with the numerical solution of Equation (7), in (*C, U*) co-ordinates, superimposed. The grey region corresponds to values of *C* where *F(C*) < 0. The dashed black lines denote a wall of singularities. Red circles correspond to equilibrium points and purple circles correspond to holes in the wall. (**b**,**e**) Numerical solution of Equation (7) at *t* = 100 and *t* = 200. The grey lines indicate the initial condition and the arrows indicate the direction of increasing time. (**c**,**f**) The time evolution of the position of the leading edge of the travelling wave solution, *L(t*). All results are obtained using 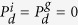, *δx* = 0.01, 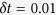, 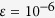 and (**a**–**c**) 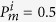, 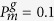, 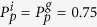, *v* = 0.864; (**d**–**f**) 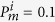, 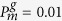, 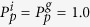, *v* = 0.448.

We superimpose the numerical solution of Equation (7) in (*C, U*) co-ordinates on the phase plane for the system (9 and 10) in Fig. 3(a,d). Details of the numerical techniques used to solve Equation (7) and to generate the phase planes are given in the Methods. The numerical solution appears to form a heteroclinic orbit between (1, 0) and (0, 0) in both cases, and passes through the holes in the wall of singularities, denoted using purple circles. Continuum models with negative diffusivity and no source terms have been relatively well studied, and exhibit shock behaviour across the region of negative diffusion^55^,^56^. Interestingly, our solution does not include a shock and is instead smooth through the region of negative diffusion.

The numerical solution of Equation (7) at *t* = 100 and *t* = 200 is shown in Fig. 3(b,e), confirming that the waveform does not change with time. To quantify the wave speed we calculate the time evolution of the leading edge, *L(t*) = *x*_*f*_ such that 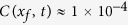. If the solution of Equation (7) forms a travelling wave, *L(t*) will tend to a straight line with slope *v*, as *t* → ∞. In Fig. 3(c,f), we observe that *L(t*) is approximately linear with slope *v*, and hence the solution of Equation (7) moves with approximately constant speed at long times. Overall, these features suggest that the solution of Equation (7) with positive-negative-positive *F(C*) approaches a travelling wave.

#### Capacity-degenerate positive-negative nonlinear diffusivity function

For the special case where 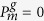, *F*_*s*_(1) = 0. Again, we consider the case with zero agent death for simplicity, and note that it is straightforward to extend the analysis for cases with non-zero agent death. As *F(C*) is degenerate at *C* = 1, it is intuitive to expect there could be sharp-fronted travelling wave solutions, with the sharp front near *C* = 1, similar to the results in ref. 57 and in the Supplementary Material. However, unlike these cases, we have an interval 1/3 < *C* < 1 where *F(C*) < 0. To determine whether this negative diffusivity influences the presence of sharp fronts, we follow the approach of Maini *et al*.^38^, who show that the existence of travelling waves for reaction-diffusion equations with capacity-degenerate positive-negative *F(C*) can be determined by considering the existence of travelling waves for


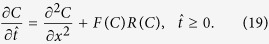


The restriction on 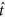 implies *F(C*) > 0. As *F(C*) < 0 for 1/3 < *C* < 1, Equation (19) is only equivalent to Equation (7) for 0 ≤ *C* ≤ 1/3. For 1/3 ≤ *C* ≤ 1, Equation (7) is equivalent to


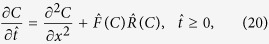


where 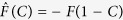 and 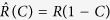
38. Equations (19 and 20) have minimum travelling wave speeds 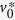 and 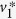, respectively. Maini *et al*.^38^ prove that sharp fronts in the travelling wave near *C* = 1 only exist if *F*(1) = 0 and 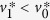. The first condition is obviously satisfied, while the second can be determined by considering the behaviour of the equivalent ordinary differential systems in travelling wave coordinates in the neighbourhood of the equilibrium points. Both equations have minimum wave speed conditions, 
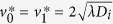
, to obtain physically-relevant heteroclinic orbits, and hence travelling wave solutions with a sharp region near *C* = 1 do not exist.

**Figure 4:**
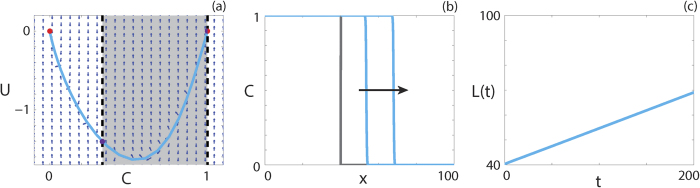
Travelling wave behaviour for Equation (7) with capacity-degenerate positive-negative *F(C*) (Case 2.4). (**a**) Phase plane for the system (9 and 10) with the numerical solution of Equation (7), in (*C, U*) co-ordinates, superimposed. The grey region corresponds to values of *C* where *F(C*) < 0. The dashed black lines denote two walls of singularities. Red circles correspond to equilibrium points and purple circles correspond to holes in the wall. (**b**) Numerical solution of Equation (7) at *t* = 100 and *t* = 200. The grey lines indicate the initial condition and the arrow indicates the direction of increasing time. (**c**) The time evolution of the position of the leading edge of the travelling wave solution, *L(t*). All results are obtained using 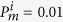, 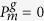, 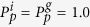, 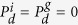, *δx* = 0.01, *δt* = 0.01, *ε* = 10^−6^, *v* = 0.1433.

Travelling wave behaviour for a parameter regime with *F*(1) = 0 is shown in Fig. 4. The equilibrium point at (1, 0) is also a hole in the wall. The solution trajectory forms a heteroclinic orbit between (1, 0) and (0, 0), and passes through the region of *C* where *F(C*) < 0. Although *F*(1) = 0, we do not observe a solution trajectory corresponding to a sharp front, as for capacity-degenerate non-negative *F(C*) (Supplementary Material). This result is consistent with the analysis of Maini *et al*.^38^. The numerical solution of Equation (4), presented in Fig. 4(b), has a relatively steep front but is not sharp near *C* = 1. As *L(t*), presented in Fig. 4(c), becomes linear as *t* increases and the waveform in Fig. 4(b) are consistent, the numerical solution of Equation (7) with *F*(1) = 0 appears to form a classic travelling wave.

#### Positive-negative nonlinear diffusivity function

The positive-negative case, where 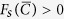 for 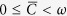 and 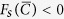 for 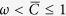, cannot occur with *K* = 0. It is instructive to examine whether stable travelling wave solutions of Equation (7) exist in such a case, as the non-zero equilibrium point now occurs in the region where 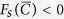. If we perform standard linear analysis on Equations (9 and 10), the Jacobian at (*S*, 0) has eigenvalues 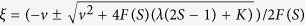, which implies that the equilibrium point is an unstable node provided 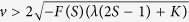. The negative sign is present as *F(S*) < 0 for positive-negative 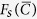. The Jacobian at (0, 0) has eigenvalues 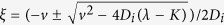, which is a stable node provided that 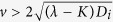. While there are infinitely many solution trajectories out of the unstable node, we require that the solution trajectory passes through the hole in the wall, and hence there is a single solution trajectory that forms a heteroclinic orbit.

**Figure 5:**
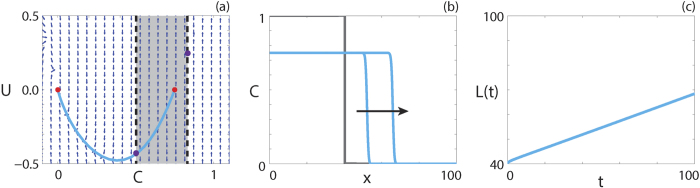
Travelling wave behaviour for Equation (7) with positive-negative 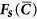 (Case 4.5). (**a**) Phase plane for the system (9 and 10) with the numerical solution of Equation (7), in (*C, U*) co-ordinates, superimposed. The grey region corresponds to values of 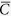 where 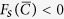. The dashed black lines denote a wall of singularities. Red circles correspond to equilibrium points and purple circles correspond to holes in the wall. (**b**) Numerical solution of Equation (7) at *t* = 50 and *t* = 100. The grey lines indicate the initial condition and the arrow indicates the direction of increasing time. (**c**) The time evolution of the position of the leading edge of the travelling wave solution. All results are obtained using 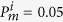, 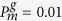, 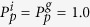, 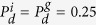, *δx* = 0.1, *δt* = 0.01, *ε* = 10^−6^, *v* = 0.2760.

Travelling wave behaviour for Equation (7) with positive-negative 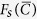 is shown in Fig. 5. The numerical solution of Equation (7), in (*C, U*) co-ordinates, passes through the wall of singularities where Equation (10) is finite and forms a heteroclinic orbit between (*S*, 0) and (0, 0). The travelling wave front is of classic type, a result predicted by the analysis performed by Maini *et al*.^38^ as 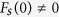 and 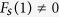.

### Allee kinetics

If the birth and death mechanisms are either competitive or co-operative, that is, 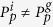 and 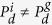, then the source term represents an Allee effect^15^ and hence Equation (2) can be expressed as


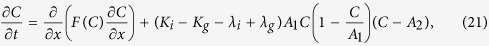


where


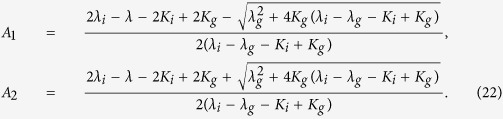


Note that either 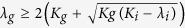 or *λ*_*i*_ > *K*_*i*_ must be satisfied or *R(C*) ≤ 0 for 0 ≤ *C* ≤ 1 and the population will tend to extinction. In travelling wave co-ordinates, Equation (21) is


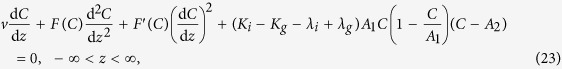


and, making the substitution *U* = d*C*/d*z*, it corresponds to


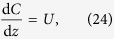



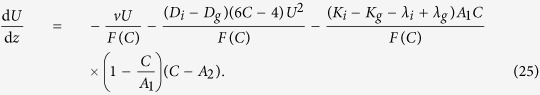


If 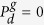, then *A*_1_ = 1, and the source term in (21) simplifies to 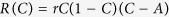, where 
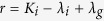
 is the intrinsic growth rate and 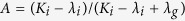 is the Allee parameter^15^.

A new variable 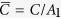 is introduced such that the range of physically relevant 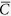 values corresponds to 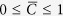. Substituting 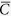 into Equation (21) results in


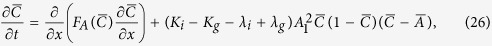


where 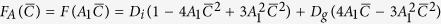 and


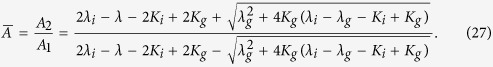


**Figure 6:**
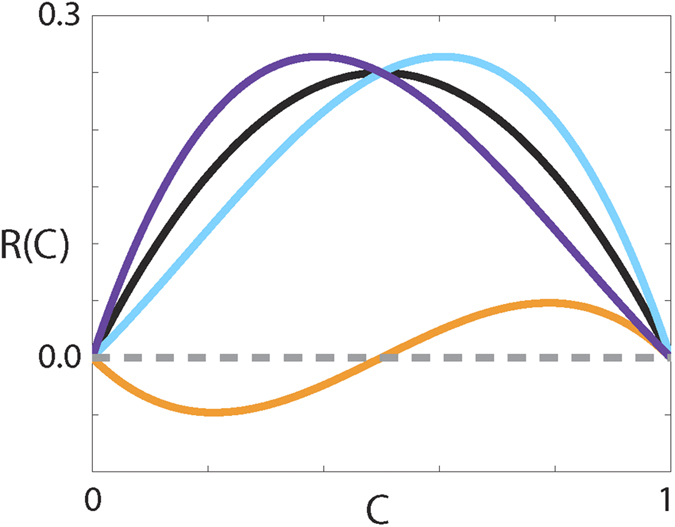
Comparison of source terms. *R(C*) corresponding to the weak Allee effect with *r* = 1, *A* = −0.5 (cyan), strong Allee effect with *r* = 1, *A* = 0.5 (orange), reverse Allee effect with *r* = −1, *A* = 1.5 (purple) and logistic growth with *r* = 1 (black).

The transformed nonlinear diffusivity, 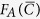, has the same characteristics as 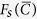, presented in Fig. 2, albeit in terms of the scaled Allee carrying capacity, *A*_1_. For 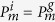, 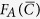 represents linear diffusion. Reaction-diffusion equations with linear diffusion and either weak or strong Allee kinetics have been well-studied^15^,^19^,^22^,^23^,^25^,^26^,^28^,^30^. For additional details we refer the reader to ref. 15. Weak Allee kinetics correspond to 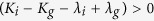 and 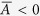, and represent a growth rate that is inhibited at low densities, compared to logistic growth, but remains positive. Strong Allee kinetics correspond to 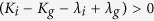 and 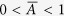
15, and represent a growth rate that is negative beneath a threshold value, and positive otherwise. Interestingly, a third type of Allee kinetics can arise from the parameter values chosen in the discrete model, that has not been considered previously. If 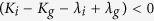 and 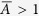, the growth rate is non-negative for all relevant 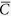 values but is inhibited at high densities, compared to logistic growth, rather than low densities like the weak Allee effect. We term this type of growth term the reverse Allee effect. Representative source terms showing the three types of Allee effect are compared with a logistic source term in Fig. 6.

For all combinations of neutral, competitive and co-operative mechanisms that give rise to a reaction-diffusion equation with Allee kinetics we examine the ability of the equation to give rise to long time travelling wave solutions. Furthermore, the three types of Allee effect arising from the discrete model are considered. Several of these cases have been presented and examined previously, but we present details about the travelling wave solutions for all combinations of diffusivity functions and Allee effects in Table 3. A detailed analysis for each case is presented in the Supplementary Material.

#### Persistence and extinction

A key question of interest for a particular class of PDE is whether the population described persists or becomes extinct in the long time limit. In all cases with Fisher kinetics with *λ* > *K*, the source term is positive for 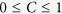, and subsequently the population persists and spreads. As the kinetics representing an Allee effect can contain a source term that is negative for an interval of *C*, it is less obvious whether the minimum wave speed is positive or negative, corresponding to persistence or extinction, respectively.

For the case with constant *F(C*) and 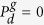, the minimum wave speed for Equation (21) with *A* < −1/2 is 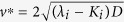 and hence the population persists, provided *λ*_*i*_ > *K*_*i*_. Introducing 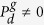 results in the same minimum wave speed, provided that 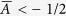. This implies that introducing grouped agent death at a rate that does not result in a population tending to extinction has no influence on the invasion speed of the population. Specifically, the condition for *A* < −1/2 with *K*_*g*_ = 0 corresponds to 3(*λ*_*i*_ − *K*_*i*_) > *λ*_*g*_. It can be shown that, with 3(*λ*_*i*_ − *K*_*i*_) > *λ*_*g*_, we require 3*K*_*g*_ < *λ*_*g*_ for 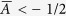. This implies that there is a range of *K*_*g*_ values that result in a travelling wave with a minimum wave speed that is independent of both *K*_*g*_ and *λ*_*g*_. Interestingly, this suggests that if a control is implemented that increases the death rate of grouped agents, there is a threshold value of 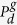 for the control to influence the invasion speed and the subsequent persistence of the population. Introducing a non-zero *K*_*g*_ value for a parameter regime that results in the strong Allee effect with *K*_*g*_ = 0 never changes the type of Allee effect. It is possible to go from a weak Allee effect to a reverse Allee effect by introducing a non-zero *K*_*g*_ value. Non-zero *K*_*g*_ values correspond to a decreased benefit for grouped agents, which explains why the source term, previously a weak Allee effect, becomes the reverse Allee effect, corresponding to inhibited growth at high density.

The reaction-diffusion equation with constant 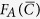 and the strong Allee effect, corresponding to 0 < *A*_2_ < *A*_1_ ≤ 1, has a unique wave speed 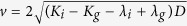 (*A*_1_/2 − *A*_2_)^28^. This implies that for *A*_2_ > *A*_1_/2, *v* < 0 and *v* > 0 otherwise. Furthermore, the same wave speed applies for –*A*_1_/2 < *A*_2_ < 0^28^. For both intervals, the minimum wave speed depends on the *K*_*g*_ value, and hence implementing any kind of partial eradication of the grouped agents will either reduce the speed of invasion or cause the extinction of the population.

For cases where 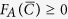 for 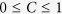 and 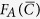 is not constant, we follow the approach of Hadeler to establish whether the minimum wave speed is positive, and hence the population persists^58^,^59^,^60^. The integral condition for the wave speed to be positive,


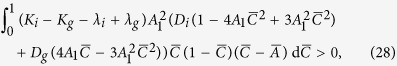


corresponds to


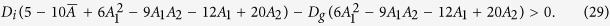


If *D*_*i*_ = *D*_*g*_, then 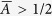 leads to *v* < 0. For the strong Allee effect, 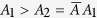, we can determine the threshold value for the persistence of the population, namely,


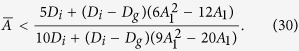


**Figure 7:**
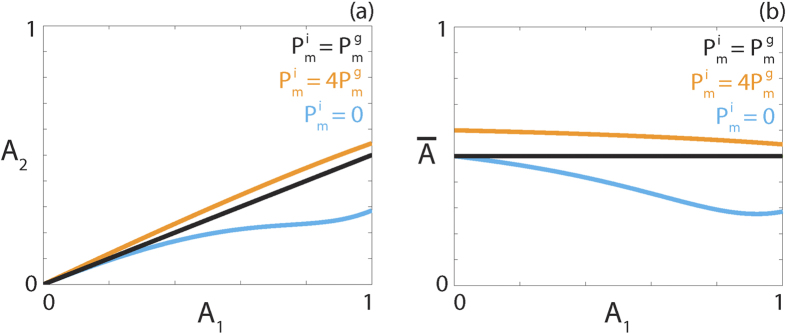
Persistence threshold. Persistence threshold as a function of the carrying capacity *A*_1_, expressed as (**a**) an explicit value; (**b**) a proportion of the carrying capacity for three different diffusivities, corresponding to 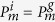 (black), 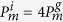 (orange) and 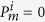 (cyan).

Considering the two limiting cases for strictly positive 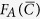, where *D*_*i*_ = 0 and *D*_*i*_ = 4*D*_*g*_, 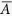 takes on a value of 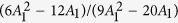 and 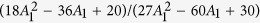, respectively. These values reduce to 6/11 and 2/7 in the case that *A*_1_ = 1, corresponding to *K*_*g*_ = 0. Therefore, populations with isolated agents that are more motile than grouped agents are less susceptible to extinction. To illustrate how the threshold value changes with *A*_1_, 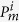 and 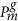, Fig. 7 shows the maximum *A*_2_ and 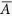 values for three different 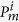 and 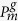 combinations. The *A*_2_ value corresponds to the persistence threshold for a given *A*_1_ value. The 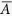 value can be interpreted as the highest proportion of a given *A*_1_ value that will result in the persistence of the population. For example, in Fig. 7(a), we see that with 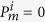 and *A*_1_ = 0.5 we require *A*_2_ < 0.194 for persistence. This corresponds to 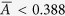.

#### Extinction-degenerate non-negative nonlinear diffusivity function

**Figure 8:**
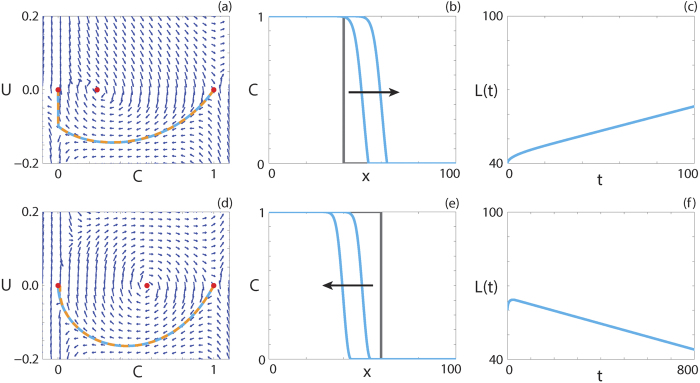
Travelling wave behaviour for Equation (21) with the strong Allee effect and extinction-degenerate non-negative *F(C*) (Case 6.2). (**a**,**d**) Phase plane for the system (24 and 25) with the numerical solution of Equations (21) (cyan, solid) and (23) (orange, dashed), in (*C, U*) co-ordinates, superimposed. Red circles correspond to equilibrium points. (**b**,**e**) Numerical solution of Equation (21) calculated at (**b**) *t* = 50 and *t* = 100; (**e**) *t* = 400 and *t* = 800. The grey lines indicate the initial condition and the arrows indicate the direction of increasing time. (**c**,**f**) The time evolution of *L(t*). All results are obtained with *δx* = 0.01, *δt* = 0.005, *ε* = 10^−6^, 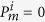, 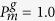, 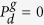, (**a**–**c**) 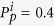, 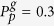, 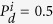, *v* = 0.199; (**d**–**f**) 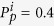, 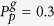, 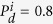, 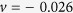.

Travelling wave behaviour for the strong Allee effect with extinction-degenerate non-negative *F(C*) is shown in Fig. 8. The numerical solution of Equation (21) with *A* = 1/4, in Fig. 8(a–c), leads to a sharp-fronted travelling wave solution near *C* = 0 with *v* > 0. With *A* = 1/4, we expect to obtain *v* > 0. For a parameter regime that results in *A* = 4/7, we obtain a travelling wave solution of Equation (21) with *v* < 0 (Fig. 8(d–f)). Interestingly, the sharp front near *C* = 0 is not present for the strong Allee effect with *v* < 0.

#### Positive-negative-positive nonlinear diffusivity function

A positive-negative-positive *F(C*), where there is an interval *α* < *C* < *β* where *F(C*) < 0, corresponds to parameter pairs highlighted in purple in Fig. 2(a). Kuzmin and Ruggerini^37^ examine reaction-diffusion equations with similar properties for the strong Allee effect, in the context of diffusion-aggregation models, and provide conditions for smooth travelling wave solutions to exist. For a solution with *v* > 0, we require *A* < *α*^37^ and


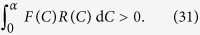


Furthermore, we require^37^


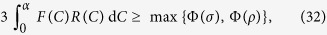


where


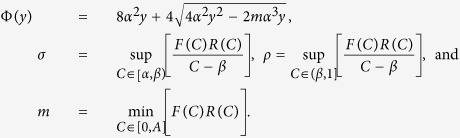


**Figure 9:**
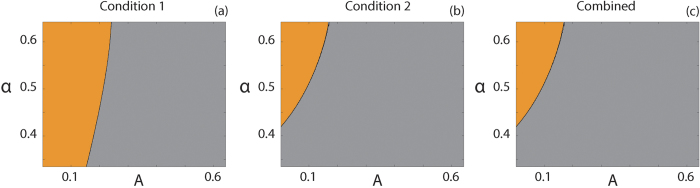
Parameter pairs that satisfy Kuzmin and Ruggerini’s Conditions. (**a**) Condition (31); (**b**) Condition (32); (**c**) Conditions (31 and 32) combined. Orange regions correspond to parameter pairs that satisfy the respective condition(s), whereas grey regions correspond to parameter pairs that do not.

A suite of 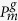 values with 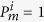 that correspond to 1/3 < *α* < 2/3 are considered for parameter regimes that result in *A* < *α*. Figure 9(a–c) show the parameter pairs, (*A, α*), that satisfy Condition (31), Condition (32) and Conditions (31 and 32) simultaneously, respectively. Orange regions represent parameter pairs where the condition is satisfied and grey regions represent parameter pairs where the condition is not satisfied. These results suggest that smooth travelling wave solutions should exist for certain choices of parameters. Interestingly, all parameter pairs that satisfy Condition (31) also satisfy Condition (32).

For Equation (7) with positive-negative-positive *F(C*), smooth travelling wave solutions that pass through holes in the wall of singularities for positive-negative-positive *F(C*) are obtained. The minimum wave speed bound presented by Ferracuti *et al*.^36^ implies that the locations of the holes in the wall occur are real-valued for the wave speed arising from the Heaviside initial condition. As such, to obtain smooth travelling wave solutions of Equation (21) with positive-negative-positive *F(C*), we might expect that the wave speed satisfies 
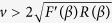
, such that the holes in the wall at *C* = *β* are real-valued.

Following the approach used for Equation (7) with positive-negative-positive *F(C*), it is simple to demonstrate that both the weak and reverse Allee effect have real-valued holes in the wall (Supplementary Material). We now examine numerical solutions of Equation (21) with the strong Allee effect. For parameter regimes that give rise to wave speeds that satisfy 
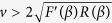
, numerical travelling wave solutions could not be found. While the condition for real-valued holes in the wall is satisfied, the zeroes of Equation (25) are imaginary for a certain interval of *C* > *β*. This corresponds to a nullcline that is not real-valued for certain *C* values.

**Figure 10:**
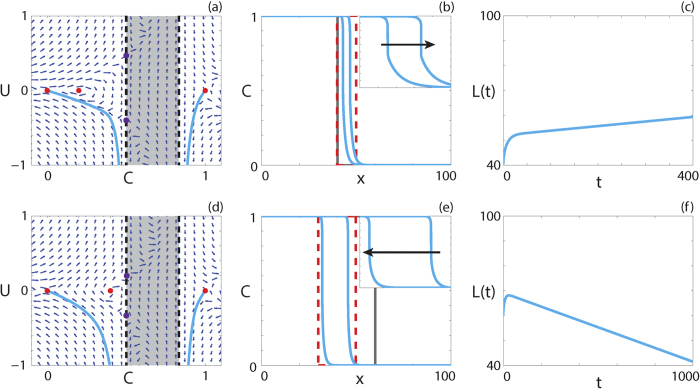
Travelling wave behaviour for Equation (21) with the strong Allee effect and positive-negative-positive *F(C*) (Case 6.3). (**a**,**d**) Phase plane for the system (24 and 25) with the numerical solution of Equation (21) (cyan, solid), in (*C, U*) co-ordinates, superimposed. The dashed black lines denote a wall of singularities. Red circles correspond to equilibrium points and purple circles correspond to holes in the wall. (**b**,**e**) Numerical solution of Equation (21) calculated at (**b**) *t* = 200 and *t* = 400; (**e**) *t* = 500 and *t* = 1000. The grey lines indicate the initial condition and the arrows indicate the direction of increasing time. The insets correspond to the areas within the red dashed lines, and highlight the shocks. (**c**,**f**) The time evolution of *L(t*). All results are obtained with *δx* = 0.05, *δt* = 0.001, *ε* = 10^−6^, 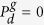, (**a**–**c**) 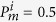, 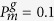, 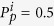, 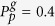, 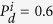, *v* = 0.009; (**d**–**f**) 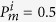, 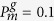, 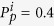, 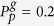, 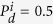, *v* = −0.028.

We now consider parameter regimes corresponding to the strong Allee effect with the additional restriction that 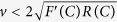 for 2/3 < *C* ≤ 1. For all 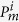 and 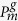 that give rise to a positive-negative-positive *F(C*), holes in the wall at *C* = *β* do not exist and, as such, we do not expect to obtain smooth solutions. Interestingly, we observe travelling wave solutions with shocks such that the solution never enters the region *α* < *C* < *β*. An example of a shock-fronted travelling wave solution for the strong Allee effect with both *v* > 0 and *v* < 0 is shown in Fig. 10(a–f), respectively. Solutions of diffusion equations, without any source terms, that contain shocks have been reported previously^55^,^56^. Similarly, shock-fronted travelling wave solutions arise in other kinds of models, including multispecies models of combustion^61^ and haptotactic cell migration^54^. However, the models presented here are very different as our model contains a source term and no advection term, and it is therefore of interest to determine the properties of the reaction-diffusion equation that lead to shock-fronted travelling wave solutions.

#### Capacity-degenerate positive-negative nonlinear diffusivity function

Capacity-degenerate positive-negative *F(C*), where *F*(1) = 0, arises if 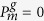 and includes an interval 1/3 < *C* < 1 where *F(C*) < 0. For the corresponding case with Fisher kinetics, despite the degenerate nature of the nonlinear diffusivity function at *C* = 1, we did not obtain solutions with a sharp front near *C* = 1. Instead, the solution passes through the region of negative diffusivity and a hole in the wall at *C* = 1/3, leading to smooth travelling wave solutions. As such, we expect similar solutions for both the weak and reverse Allee effect due to the qualitatively similar behaviour of the *R(C*) function. It is of interest to examine whether smooth or shock-fronted travelling wave solutions arise from Equation (21) for the strong Allee effect and capacity-degenerate positive-negative *F(C*), as for the positive-negative-positive *F(C*) no smooth travelling wave solutions could be found.

As expected, smooth travelling wave solutions for both the weak and reverse Allee effects with capacity-degenerate positive-negative *F(C*) are obtained. The solution behaviour for both the weak and reverse Allee effects are presented in the Supplementary Material. For the strong Allee effect, we examined a considerable number of parameter regimes and initial conditions and were unable to find travelling wave solutions.

#### Positive-negative nonlinear diffusivity function

For the case where 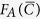 has exactly one zero on the interval 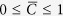 at 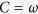, Maini *et al*.^39^ examine the existence of travelling wave solutions, and provide the necessary conditions for existence,


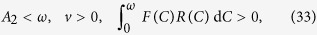


where 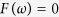 and 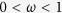. For the strong Allee effect in this parameter regime, the third part of Condition (33) corresponds to


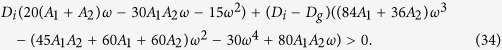


Equation (21) is equivalent to


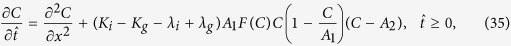


on the interval 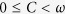, and equivalent to


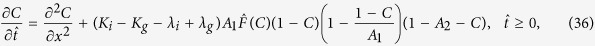


where 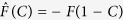, on the interval 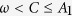. The final necessary and sufficient condition from Maini *et al*.^39^ for the existence of travelling wave solutions is that the minimum wave speed for Equation (35), 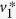, is greater than, or equal to, the minimum wave speed for Equation (36), 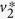. On the interval 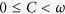, Equation (21) has a strictly positive *F(C*), where *F(C*) ≤ *D*_*i*_, and strong Allee kinetics. Hence, the minimum wave speed for Equation (35) has an upper bound, 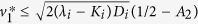. On the interval 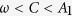 Equation (36) has a source term qualitatively similar to the Fisher-Kolmogorov equation and hence a lower bound for the minimum wave speed exists^39^, 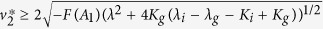. For all parameter regimes considered that correspond to the strong Allee effect with positive-negative 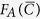 we never observe a case where the upper bound for 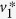 is higher than the lower bound for 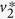 and hence the conditions required for travelling wave solutions are not met. As expected, numerical solutions of Equation (21) in these parameter regimes did not lead to travelling wave behaviour.

## Discussion

In this work we present a discrete lattice-based model of birth, death and movement. The model is an exclusion process, and hence it explicitly incorporates crowding by allowing no more than one agent per site. A key feature of the model is that birth, death and movement rates depend on whether an agent is isolated or whether it is part of a group of agents. The discrete model can, therefore, be used to describe co-operative or competitive mechanisms^8^,^13^,^14^,^15^. These kinds of mechanisms are thought to be relevant to many applications in cell biology^8^,^12^,^62^,^63^ and ecology^13^,^14^,^15^. By considering different combinations of parameters, the continuum limit PDE approximation of the discrete model leads to 22 different cases. These cases are reaction-diffusion equations with either Fisher kinetics or Allee kinetics, and a variety of density-dependent nonlinear diffusivity functions (Table 1). This approach also leads to a new kind of Allee effect, which we call the reverse Allee effect, where the growth rate is inhibited at high density. Although some of the PDEs that we consider have been investigated previously^15^,^18^,^19^,^20^,^21^,^22^,^23^,^24^,^25^,^26^,^27^,^28^,^29^,^30^,^36^,^37^,^38^,^39^,^40^,^41^, they have never been linked together before using a single modelling framework. The presence of Allee kinetics allows for the more realistic description of biological and ecological phenomena, as the standard reaction-diffusion model with Fisher kinetics predicts either the population tending to extinction everywhere or the spread of the population in the form of a travelling wave. In comparison, Allee kinetics can describe population retreat, as well as shocks in the invading front of a population. Well-defined edges are thought to be present in invasive tumours^63^, which can be described with travelling waves containing shocks.

In this work, we summarise properties of the long time travelling wave solutions for all classes of PDEs arising from our discrete model. For certain PDEs, where only existence of travelling wave solutions has been considered previously, we present numerical solutions here for the first time. We find that PDE models with density-dependent nonlinear diffusivity functions that have regions of negative diffusivity require a sufficiently non-negative source term to support smooth travelling wave solutions. Furthermore, there appears to be a threshold proliferation value, depending on the rate of motility, that must be exceeded for travelling wave solutions to be observed numerically. However, we do not comment on the putative relationship between the parameters in the discrete model and the existence of travelling wave solutions in the continuum limit PDE. Interestingly, for the strong Allee effect, shock-fronted travelling wave solutions are obtained. Following arguments presented in ref. 39, we show that smooth travelling wave solutions cannot be obtained for certain types of nonlinear diffusivity functions and the strong Allee effect. We describe how nonlinear diffusion can either hinder or promote the persistence of a population, depending on the relative motility rates of the isolated and grouped agents. Interestingly, the motility rates affect the persistence differently for different carrying capacities. This relationship could provide insight into the requirements for a cell population, for example, to persist in the presence of a chemical treatment.

The six birth, death and motility rate parameters in the discrete model allow for the interpretation of the results in terms of whether individuals are part of, or isolated from, the bulk population. For example, a parameter regime corresponding to the strong Allee effect with constant diffusivity and no grouped agent death leads to the same travelling wave speed in the PDE description as a parameter regime corresponding to the strong Allee effect with constant diffusivity and a non-zero rate of grouped agent death, up to a threshold. This implies that a sufficiently strong intervention strategy aimed at grouped agents must be implemented if the goal of the intervention is to slow or halt the invasion of a population.

The work presented here suggests several avenues for future research. This work could be generalised by considering a two- or three–dimensional discrete process and deriving the continuum limit PDE descriptions in higher dimensions. This kind of higher-dimensional model might provide a more accurate description of real world observations where one-dimensional travelling wave solutions might not apply. In this work, numerical travelling wave solutions for each class of PDE are examined, but the formal stability of these travelling wave solutions is not considered. Another approach for analysing the discrete model would be to consider a coupled multispecies PDE model by accounting for the density of isolated agents and the density of grouped agents separately. This approach would lead to a system of two coupled PDEs instead of a single PDE for the total agent density. However, instead of working with coupled multispecies PDEs, we have taken the simplest and most fundamental approach of considering a single PDE description of the total population. In addition, a significant number of mechanisms could be implemented into the discrete model, such as cell-to-cell adhesion/repulsion^42^,^64^ or directed migration of isolated agents, such as chemotaxis^65^. We leave these extensions for future analysis.

## Methods

### Discrete model

We consider a one-dimensional lattice-based random walk with *X* sites and lattice spacing Δ^48^. Each site may be occupied by, at most, one agent^43^,^44^,^66^. The number of agents at time *t* is *N(t*). Agents attempt to undergo birth, death and movement events. During a birth event, an agent attempts to place a daughter agent at a randomly selected nearest-neighbour site. This event is successful provided that the selected site is vacant. During a death event, an agent is removed from the lattice. During a movement event, an agent attempts to move to a randomly selected nearest-neighbour site. This event is successful provided that the selected site is vacant. We distinguish between types of agents based on the number of occupied nearest-neighbour sites for each agent^45^. We refer to agents with zero occupied nearest-neighbour sites as *isolated agents*, and agents with one or two occupied nearest-neighbour sites as *grouped agents*. This approach allows us to specify different birth, death and movement rates for isolated and grouped agents.

Different parameter choices can be used to impose either co-operative or competitive mechanisms, where an increase in local agent density provides a positive or negative benefit, respectively. Specifically, in situations where the group motility or group proliferation rates are higher than the isolated motility or isolated proliferation rates, respectively, we interpret this choice of parameters as a model of co-operation. Similarly, in situations where the group motility or group proliferation rates are lower than the isolated motility or isolated proliferation rates, respectively, we interpret this as a model of competition.

During each time step of duration *τ, N(t*) agents are selected at random, one at a time, with replacement, and are given the opportunity to undergo a movement event. The constant probability that a selected agent attempts to undergo a movement event is 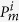 for an isolated agent and 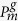 for a grouped agent. We repeat this process for both birth and death events, with respective constant probabilities 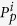 and 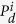 for isolated agents and 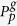 and 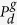 for an agent within a group. At the end of each time step we update *N(t* + *τ*). To obtain the average agent density at each lattice site we perform *M* identically-prepared realisations of the discrete model and average the binary lattice occupancy at each lattice site at each time step. In any single realisation of the discrete model we have *C*_*j*_ = 1 when site *j* is occupied and *C*_*j*_ = 0 when site *j* is vacant. To evaluate the average occupancy of any lattice site we consider an ensemble of *M* identically-prepared realisations and calculate 
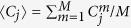
.

### Numerical techniques

Here we describe the techniques used to obtain numerical solutions of Equation (2), the corresponding ODE in travelling wave co-ordinates, and to generate the phase planes in (*C, U*) co-ordinates.

#### Partial differential equations

To obtain numerical solutions of Equation (2), we first spatially discretise Equation (2) onto a grid with uniform grid spacing *δx* by approximating the spatial derivatives with a central finite difference approximation. A backward Euler approximation with constant time steps of duration *δt* is used to approximate the temporal derivative. The resulting system of nonlinear algebraic equations is solved using Picard iteration with absolute convergence tolerance *ε*. The resulting system of tridiagonal algebraic equations is solved using the Thomas algorithm^67^. All results presented correspond to sufficiently small choices of *δx, δt* and *ε* so that the numerical solutions are grid independent. In all cases consider zero-flux boundary conditions are considered, and the finite domain is sufficiently large such that the numerical solution of Equation (2) does not interact with the boundaries on the time scale of the numerical simulations. All numerical solutions correspond to a Heaviside initial condition with *C* = 1 for *x* ≤ *X*_0_, and *C* = 0 otherwise.

#### Ordinary differential equations

The second order ODEs in the travelling wave co-ordinates are solved using Matlab’s ode45 routine^68^. This routine implements an adaptive Runge-Kutta method with relative error tolerance of 10^−3^ and an absolute error tolerance of 10^−6 ^68. Travelling wave ODEs that contain a singularity are not solved numerically. Therefore, for these singular problems we obtain only the numerical solution of the PDE and present this solution in the transformed (*C, U*) travelling wave co-ordinate system.

#### Phase planes

To generate phase planes we substitute *U* = d*C*/d*z* into the second order travelling wave ODE to obtain a system of two first-order ODEs. The phase plane is constructed by considering 22 equally-spaced values of *C* and 22 equally spaced values of *U* to calculate both d*C*/d*z* and d*U*/d*z* at all 22 × 22 = 484 pairs of (*C, U*) values. In each phase plane the same 22 equally spaced values of *C* on the interval 0 ≤ *C* ≤ 1 are considered. However, depending on the steepness of the waveform, we choose a different interval of *U* to construct the phase plane, and this choice is made to accommodate the heteroclinic orbit. The phase planes are constructed using Matlab’s quiver function. The location of the equilibrium points, where d*C*/d*z* = d*U*/d*z* = 0 are superimposed. Furthermore, in many cases the expression for d*U*/d*z* has a rational form, 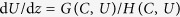. In these cases both the wall of singularities (*H(C, U*) = 0) and the locations of the holes in the wall (*H(C, U*) = *G(U, C*) = 0) are also superimposed.

## Additional Information

**How to cite this article**: Johnston, S. T. *et al*. Co-operation, Competition and Crowding: A Discrete Framework Linking Allee Kinetics, Nonlinear Diffusion, Shocks and Sharp-Fronted Travelling Waves. *Sci. Rep.* **7**, 42134; doi: 10.1038/srep42134 (2017).

**Publisher's note:** Springer Nature remains neutral with regard to jurisdictional claims in published maps and institutional affiliations.

## Supplementary Material

Supplementary Material

## Figures and Tables

**Table 1:** Different classes of PDE associated with the discrete model in appropriate parameter regimes.

	Nonlinear diffusivity	Degenerate diffusivity	Number of non-degenerate zeroes	Negative diffusivity	Source Term	Grouped agent death	Relevant previous analysis
Case 1	x	x	0	x	Fisher	x	18, 19, 20, 21,24,25,27,29
Case 2: Sub-case 1	✓	x	0	x	Fisher	x	58, 59, 60
Case 2: Sub-case 2	✓	✓	0	x	Fisher	x	40,57,69,70
Case 2: Sub-case 3	✓	x	2	✓	Fisher	x	36
Case 2: Sub-case 4	✓	✓	1	✓	Fisher	x	38
Case 3	x	x	0	x	Fisher	✓	18, 19, 20, 21,24,25,27
Case 4: Sub-case 1	✓	x	0	x	Fisher	✓	58, 59, 60
Case 4: Sub-case 2	✓	✓	0	x	Fisher	✓	40,57,69,70
Case 4: Sub-case 3	✓	x	2	✓	Fisher	✓	36
Case 4: Sub-case 4	✓	✓	1	✓	Fisher	✓	38
Case 4: Sub-case 5	✓	x	1	✓	Fisher	✓	38
Case 5	x	x	0	x	Allee	x	15,19,22,23,25,26,28,30
Case 6: Sub-case 1	✓	x	0	x	Allee	x	69,71
Case 6: Sub-case 2	✓	✓	0	x	Allee	x	41,72
Case 6: Sub-case 3	✓	x	2	✓	Allee	x	37
Case 6: Sub-case 4	✓	✓	1	✓	Allee	x	39
Case 7	x	x	0	x	Allee	✓	15,19,22,23,25,26,28,30
Case 8: Sub-case 1	✓	x	0	x	Allee	✓	69,71
Case 8: Sub-case 2	✓	✓	0	x	Allee	✓	41,72
Case 8: Sub-case 3	✓	x	2	✓	Allee	✓	37
Case 8: Sub-case 4	✓	✓	1	✓	Allee	✓	39
Case 8: Sub-case 5	✓	x	1	✓	Allee	✓	39

An Allee source term can correspond to either the weak, strong or reverse Allee effect. Degenerate diffusivity refers to the case where F(C*) = R(C*) = 0 for some value C*. A detailed analysis of all cases is presented in the Supplementary Material.

**Table 2:** Classification of travelling wave solutions arising from different classes of PDEs with Fisher kinetics.

Diffusivity function classification	Travelling wave	Front type	Direction
Constant	Yes	Smooth	Positive
Strictly positive	Yes	Smooth	Positive
Extinction-degenerate non-negative	Yes	Sharp	Positive
**Positive-negative-positive**	**Yes**	**Smooth**	**Positive**
**Capacity-degenerate positive-negative**	**Yes**	**Smooth**	**Positive**
**Positive-negative**	**Yes**	**Smooth**	**Positive**

Bold entries refer to cases analysed in detail in the manuscript; the other cases are analysed in the Supplementary Material.

**Table 3:** Classification of travelling wave solutions arising from different classes of PDEs with Allee kinetics.

Diffusivity function classification	Allee effect	Travelling wave	Front type	Direction
Constant	Weak/Reverse	Yes	Smooth	Positive
Constant	Strong	Yes	Smooth	Positive
Constant	Strong	Yes	Smooth	Negative
Strictly positive	Weak/Reverse	Yes	Smooth	Positive
Strictly positive	Strong	Yes	Smooth	Positive
Strictly positive	Strong	Yes	Smooth	Negative
Extinction-degenerate non-negative	Weak/Reverse	Yes	Sharp	Positive
**Extinction-degenerate non-negative**	**Strong**	**Yes**	**Sharp**	**Positive**
**Extinction-degenerate non-negative**	**Strong**	**Yes**	**Smooth**	**Negative**
Positive-negative-positive	Weak/Reverse	Yes	Smooth	Positive
**Positive-negative-positive**	**Strong**	**Yes**	**Shock**	**Positive**
**Positive-negative-positive**	**Strong**	**Yes**	**Shock**	**Negative**
Capacity-degenerate positive-negative	Weak/Reverse	Yes	Smooth	Positive
**Capacity-degenerate positive-negative**	**Strong**	**No**	**N/A**	**N/A**
Positive-negative	Weak/Reverse	Yes	Smooth	Positive
**Positive-negative**	**Strong**	**No**	**N/A**	**N/A**

Bold entries refer to cases analysed in detail in the manuscript; the other cases are analysed in the Supplementary Material.

## References

[b1] Alvord E. C. Jr. & Shaw C. M. Neoplasms affecting the nervous system of the elderly (Oxford University Press, 1991).

[b2] Clavero M. & García-Berthou E. Invasive species are a leading cause of animal extinctions. Trends in Ecology and Evolution 20, 110–110 (2005). 16701353 10.1016/j.tree.2005.01.003

[b3] Gabriely G. . MicroRNA 21 promotes glioma invasion by targeting matrix metalloproteinase regulators. Molecular and Cellular Biology 28, 5369–5380 (2008). 18591254 10.1128/MCB.00479-08 PMC2519720

[b4] Gerlee P. & Nelander S. The impact of phenotypic switching on glioblastoma growth and invasion. PLoS Computational Biology 8, e1002556 (2012). 22719241 10.1371/journal.pcbi.1002556 PMC3375261

[b5] Gerlee P. & Nelander S. Travelling wave analysis of a mathematical model of glioblastoma growth. Mathematical Biosciences 276, 75–81 (2016). 27021919 10.1016/j.mbs.2016.03.004

[b6] Johnston S. T., Simpson M. J. & McElwain D. L. S. How much information can be obtained from tracking the position of the leading edge in a scratch assay? Journal of The Royal Society Interface 11, 20140325 (2014). 10.1098/rsif.2014.0325 PMC4208362 24850906

[b7] Martin P. Wound healing – aiming for perfect skin regeneration. Science 276, 75–81 (1997). 9082989 10.1126/science.276.5309.75

[b8] Poujade M. . Collective migration of an epithelial monolayer in response to a model wound. Proceedings of the National Academy of Sciences 104, 15988–15993 (2007). 10.1073/pnas.0705062104 PMC2042149 17905871

[b9] Sherratt J. A., Lewis M. A. & Fowler A. C. Ecological chaos in the wake of invasion. Proceedings of the National Academy of Sciences 92, 2524–2528 (1995). 10.1073/pnas.92.7.2524 PMC42250 7708678

[b10] Swanson K. R., Bridge C., Murray J. D. & Alvord E. C. Virtual and real brain tumors: using mathematical modeling to quantify glioma growth and invasion. Journal of the Neurological Sciences 216, 1–10 (2003). 14607296 10.1016/j.jns.2003.06.001

[b11] Townsend C. R. Invasion biology and ecological impacts of brown trout *salmo trutta* in New Zealand. Biological Conservation 78, 13–22 (1996).

[b12] Axelrod R., Axelrod D. E. & Pienta K. J. Evolution of cooperation among tumor cells. Proceedings of the National Academy of Sciences 103, 13474–13479 (2006). 10.1073/pnas.0606053103 PMC1557388 16938860

[b13] Courchamp F., Clutton-Brock T. & Grenfell B. Inverse density dependence and the Allee effect. Trends in Ecology and Evolution 14, 405–410 (1999). 10481205 10.1016/s0169-5347(99)01683-3

[b14] Godin J. G. J. Antipredator function of shoaling in teleost fishes: a selective review. Le Naturaliste Canadien 113, 241–250 (1986).

[b15] Taylor C. M. & Hastings A. Allee effects in biological invasions. Ecology Letters 8, 895–908 (2005).

[b16] Semmens B. X. . Quasi-extinction risk and population targets for the Eastern, migratory population of monarch butterflies (danaus plexippus). Scientific Reports 6, 23265 (2016). 26997124 10.1038/srep23265 PMC4800428

[b17] Wang J. . A framework for the assessment of the spatial and temporal patterns of threatened coastal delphinids. Scientific Reports 6, 19883 (2016). 26804347 10.1038/srep19883 PMC4726438

[b18] Ablowitz M. J. & Zeppetella A. Explicit solutions of Fisher’s equation for a special wave speed. Bulletin of Mathematical Biology 41, 835–840 (1979).

[b19] Aronson D. G. & Weinberger H. F. Multidimensional nonlinear diffusion arising in population genetics. Advances in Mathematics 30, 33–76 (1978).

[b20] Bramson M. Convergence of solutions of the Kolmogorov equation to travelling waves (American Mathematical Society 1983).

[b21] Canosa J. On a nonlinear diffusion equation describing population growth. IBM Journal of Research and Development 17, 307–313 (1973).

[b22] Fife P. C. & McLeod J. B. The approach of solutions of nonlinear diffusion equations to travelling front solutions. Archive for Rational Mechanics and Analysis 65, 335–361 (1977).

[b23] Fife P. C. Long time behavior of solutions of bistable nonlinear diffusion equations. Archive for Rational Mechanics and Analysis 70, 31–36 (1979).

[b24] Fisher R. A. The wave of advance of advantageous genes. Annals of Eugenics 7, 355–369 (1937).

[b25] Hadeler K. P. & Rothe F. Travelling fronts in nonlinear diffusion equations. Journal of Mathematical Biology 2, 251–263 (1975).

[b26] Keitt T. H., Lewis M. A. & Holt R. D. Allee effects, invasion pinning, and species borders. The American Naturalist 157, 203–216 (2001). 10.1086/318633 18707272

[b27] Kolmogorov A. N., Petrovsky I. G. & Piskunov N. S. Étude de léquation de la diffusion avec croissance de la quantité de matiere et son application un probleme biologique. Moscow University Mathematics Bulletin 1, 1–25 (1937).

[b28] Lewis M. A. & Kareiva P. Allee dynamics and the spread of invading organisms. Theoretical Population Biology 43, 141–158 (1993).

[b29] Murray J. D. Mathematical Biology I: An Introduction (Springer, New York, 2002).

[b30] Rothe F. Convergence to pushed fronts. Journal of Mathematics 11, 617–634 (1981).

[b31] Johnston S. T., Shah E. T., Chopin L. K., McElwain D. L. S. & Simpson M. J. Estimating cell diffusivity and cell proliferation rate by interpreting IncuCyte ZOOM assay data using the Fisher-Kolmogorov model. BMC Systems Biology 9, 38 (2015). 26188761 10.1186/s12918-015-0182-y PMC4506581

[b32] Maini P. K., McElwain D. L. S. & Leavesley D. Travelling waves in a wound healing assay. Applied Mathematics Letters 17, 575–580 (2004).

[b33] Maini P. K., McElwain D. L. S. & Leavesley D. I. Traveling wave model to interpret a wound-healing cell migration assay for human peritoneal mesothelial cells. Tissue Engineering 10, 475–482 (2004). 15165464 10.1089/107632704323061834

[b34] Sherratt J. A. & Murray J. D. Models of epidermal wound healing. Proceedings of the Royal Society of London B: Biological Sciences 241, 29–36 (1990). 10.1098/rspb.1990.0061 1978332

[b35] Ito H. . Evolution of periodicity in periodical cicadas. Scientific Reports 5, 14094 (2015). 26365061 10.1038/srep14094 PMC4568538

[b36] Ferracuti L., Marcelli C. & Papalini F. Travelling waves in some reaction-diffusion-aggregation models. Advances in Dynamical Systems and Applications 4, 19–33 (2009).

[b37] Kuzmin M. & Ruggerini S. Front propagation in diffusion-aggregation models with bi-stable reaction. Discrete and Continuous Dynamical Systems: Series B 16, 819–833 (2011).

[b38] Maini P. K., Malaguti L., Marcelli C. & Matucci S. Diffusion-aggregation processes with mono-stable reaction terms. Discrete and Continuous Dynamical Systems: Series B 6, 1175–1189 (2006).

[b39] Maini P. K., Malaguti L., Marcelli C. & Matucci S. Aggregative movement and front propagation for bi-stable population models. Mathematical Models and Methods in Applied Sciences 17, 1351–1368 (2007).

[b40] Sánchez-Garduño F. & Maini P. K. Existence and uniqueness of a sharp travelling wave in degenerate non-linear diffusion Fisher-KPP equations. Journal of Mathematical Biology 33, 163–192 (1994).

[b41] Sánchez-Garduño F. & Maini P. K. Travelling wave phenomena in non-linear diffusion degenerate Nagumo equations. Journal of Mathematical Biology 35, 713–728 (1997).

[b42] Johnston S. T., Simpson M. J. & Baker R. E. Mean-field descriptions of collective migration with strong adhesion. Physical Review E 85, 051922 (2012). 10.1103/PhysRevE.85.051922 23004802

[b43] Simpson M. J., Landman K. A. & Hughes B. D. Cell invasion with proliferation mechanisms motivated by time-lapse data. Physica A: Statistical Mechanics and its Applications 389, 3779–3790 (2010).

[b44] Baker R. E. & Simpson M. J. Correcting mean-field approximations for birth-death-movement processes. Physical Review E 82, 041905 (2010). 10.1103/PhysRevE.82.041905 21230311

[b45] Johnston S. T., Baker R. E. & Simpson M. J. Filling the gaps: A robust description of adhesive birth-death-movement processes. Physical Review E 93, 042413 (2016). 27176335 10.1103/PhysRevE.93.042413

[b46] Simpson M. J. & Baker R. E. Corrected mean-field models for spatially dependent advection-diffusion-reaction phenomena. Physical Review E 83, 051922 (2011). 10.1103/PhysRevE.83.051922 21728586

[b47] Johnston S. T., Simpson M. J. & Baker R. E. Modelling the movement of interacting cell populations: a moment dynamics approach. Journal of Theoretical Biology 370, 81–92 (2015). 25644755 10.1016/j.jtbi.2015.01.025

[b48] Codling E. A., Plank M. J. & Benhamou S. Random walk models in biology. Journal of the Royal Society Interface 5, 813–834 (2008). 10.1098/rsif.2008.0014 PMC2504494 18426776

[b49] Hughes B. D. Random Walks and Random Environments vol. 1 (Clarendon Press, Oxford University Press, 1995).

[b50] Briscoe B. K., Lewis M. A. & Parrish S. E. Home range formation in wolves due to scent marking. Bulletin of Mathematical Biology 64, 261–284 (2002). 11926117 10.1006/bulm.2001.0273

[b51] Turchin P. Population consequences of aggregative movement. Journal of Animal Ecology 58, 75–100 (1989).

[b52] Lews M. A. Spatial coupling of plant and herbivore dynamics: the contribution of herbivore dispersal to transient and persistent “waves” of damage. Theoretical Population Biology 45, 277–312 (1994).

[b53] Pettet G. J., McElwain D. L. S. & Norbury J. Lotka-volterra equations with chemotaxis: Walls, barriers and travelling waves. Mathematical Medicine and Biology 17, 395–413 (2000). 11270751

[b54] Wechselberger M. & Pettet G. J. Folds, canards and shocks in advection–reaction–diffusion models. Nonlinearity 23, 1949 (2010).

[b55] Landman K. A. & White L. R. Terraced spreading of nanofilms under a nonmonotonic disjoining pressure. Physics of Fluids 23, 012004 (2011).

[b56] Witelski T. P. Shocks in nonlinear diffusion. Applied Mathematics Letters 8, 27–32 (1995).

[b57] Sherratt J. A. & Marchant B. P. Nonsharp travelling wave fronts in the Fisher equation with degenerate nonlinear diffusion. Applied Mathematics Letters 9, 33–38 (1996).

[b58] Hadeler K. P. Travelling fronts and free boundary value problems. In Numerical Treatment of Free Boundary Value Problems, 90–107 (Springer, 1982).

[b59] Hadeler K. P. Free boundary problems in biological models. Free boundary problems: Theory and applications 2, 664–671 (1983).

[b60] Hadeler K. P. Traveling fronts in parabolic and hyperbolic equations. In Dynamical Systems, 154–164 (Springer, 1987).

[b61] Forbes L. K. & Derrick W. A combustion wave of permanent form in a compressible gas. The ANZIAM Journal 43, 35–58 (2001).

[b62] Korolev K. S., Xavier J. B. & Gore J. Turning ecology and evolution against cancer. Nature Reviews Cancer 14, 371–380 (2014). 24739582 10.1038/nrc3712 PMC13213539

[b63] Sewalt L., Harley K., van Heijster P. & Balasuriya S. Influences of Allee effects in the spreading of malignant tumours. Journal of Theoretical Biology 394, 77–92 (2016). 26802481 10.1016/j.jtbi.2015.12.024

[b64] Anguige K. & Schmeiser C. A one-dimensional model of cell diffusion and aggregation, incorporating volume filling and cell-to-cell adhesion. Journal of Mathematical Biology 58, 395–427 (2009). 18563413 10.1007/s00285-008-0197-8

[b65] Keller E. F. & Segel L. A. Model for chemotaxis. Journal of Theoretical Biology 30, 225–234 (1971). 4926701 10.1016/0022-5193(71)90050-6

[b66] Chowdhury D., Schadschneider A. & Nishinari K. Physics of transport and traffic phenomena in biology: from molecular motors and cells to organisms. Physics of Life Reviews 2, 318–352 (2005). 10.1016/j.plrev.2024.11.008 39571466

[b67] Press W. H. Numerical Recipes 3rd Edition: The Art of Scientific Computing (Cambridge University Press, 2007).

[b68] Shampine L. F. & Reichelt M. W. The Matlab ODE suite. SIAM Journal on Scientific Computing 18, 1–22 (1997).

[b69] Malaguti L. & Marcelli C. Sharp profiles in degenerate and doubly degenerate Fisher-KPP equations. Journal of Differential Equations 195, 471–496 (2003).

[b70] Sherratt J. A. On the form of smooth-front travelling waves in a reaction-diffusion equation with degenerate nonlinear diffusion. Mathematical Modelling of Natural Phenomena 5, 64–79 (2010).

[b71] Padrón V. Effect of aggregation on population recovery modeled by a forward-backward pseudoparabolic equation. Transactions of the American Mathematical Society 356, 2739–2756 (2004).

[b72] Malaguti L., Marcelli C. & Matucci S. Front propagation in bistable reaction-diffusion-advection equations. Advances in Differential Equations 9, 1143–1166 (2004).

