# Supplementary material for: Co-operation, Competition and Crowding: A Discrete Framework Linking Allee Kinetics, Nonlinear Diffusion, Shocks and Sharp-Fronted Travelling Waves
Source: Sci Rep. 2017 Feb 14;7:42134. doi: 10.1038/srep42134 (PMC5307329; doi:10.1038/srep42134)
Supplement: Supplementary Material [file srep42134-s1.pdf]

SUPPLEMENTARY MATERIAL FOR 'CO-OPERATION, COMPETITION AND CROWDING: A DISCRETE FRAMEWORK LINKING ALLEE KINETICS, NONLINEAR DIFFUSION, SHOCKS AND SHARP-FRONTED TRAVELLING WAVES'

STUART T. JOHNSTON[1,2], RUTH E. BAKER[3], D.L SEAN MCELWAIN[1,2], AND MATTHEW J. SIMPSON*[1,2]

Case 1. Equal motility rates, equal proliferation rates, no agent death 2
Case 2. Different motility rates, equal proliferation rates, no agent death 3
Sub-case 2.1. Strictly positive nonlinear diffusivity function 4
Sub-case 2.2. Extinction-degenerate non-negative nonlinear diffusivity function 6
Sub-case 2.3. Positive-negative-positive nonlinear diffusivity function 7
Sub-case 2.4. Capacity-degenerate positive-negative nonlinear diffusivity function 10
Case 3. Equal motility rates, equal proliferation rates, equal death rates 11
Case 4. Different motility rates, equal proliferation rates, equal death rates 13
Sub-case 4.1. Strictly positive nonlinear diffusivity function 14
Sub-case 4.2. Extinction-degenerate non-negative nonlinear diffusivity function 16
Sub-case 4.3. Positive-negative-positive nonlinear diffusivity function 16
Sub-case 4.4. Capacity-degenerate positive-negative nonlinear diffusivity function 16
Sub-case 4.5. Positive-negative nonlinear diffusivity function 16
Case 5. Equal motility rates, no grouped agent death 17
Case 6. Different motility rates, no grouped agent death 21
Sub-case 6.1. Strictly positive nonlinear diffusivity function 21
Sub-case 6.2. Extinction-degenerate non-negative nonlinear diffusivity function 22
Sub-case 6.3. Positive-negative-positive nonlinear diffusivity function 23
Sub-case 6.4. Capacity-degenerate positive-negative nonlinear diffusivity function 27
Case 7. Equal motility rates, different death rates 28
Case 8. Different motility rates, different death rates 30
Sub-case 8.1. Strictly positive nonlinear diffusivity function 32
Sub-case 8.2. Extinction-degenerate non-negative nonlinear diffusivity function 32
Sub-case 8.3. Positive-negative-positive nonlinear diffusivity function 33
Sub-case 8.4. Capacity-degenerate positive-negative nonlinear diffusivity function 33
Sub-case 8.5. Positive-negative nonlinear diffusivity function 35
References 36

This supplementary material for the manuscript 'Co-operation, competition and crowding: a discrete framework linking Allee kinetics, nonlinear diffusion, shocks and sharp-fronted travelling waves' contains a more detailed analysis of all of the different cases of partial differential equations (PDEs) that arise from the continuum description of the discrete model of co-operation and competition. Eight cases of different combinations of co-operative, competitive or neutral mechanisms are now considered systematically. Some of these cases involve distinct sub-cases so that, in total, we consider 22 different classes of PDE models of invasion. The properties of each of these distinct cases are presented in Table 1 in the main document. Note that certain cases are examined in the main document and hence there is some unavoidable overlap between the two documents, as here we examine all cases in a systematic manner.

* Corresponding author: Matthew J. Simpson (matthew.simpson@qut.edu.au).
[1]Mathematical Sciences, Queensland University of Technology (QUT), Brisbane, Australia.
[2]Tissue Repair and Regeneration Program, Institute of Health and Biomedical Innovation, QUT, Brisbane, Australia.
[3]Wolfson Centre for Mathematical Biology, Mathematical Institute, University of Oxford, United Kingdom.

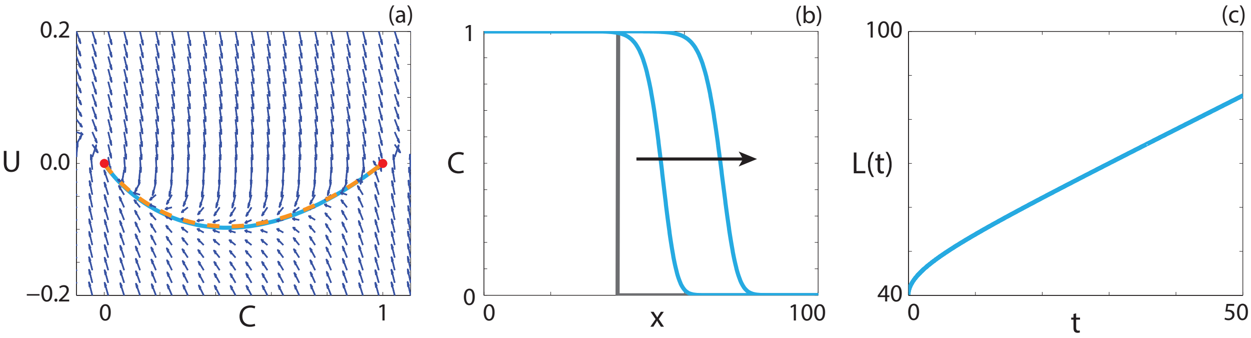

FIGURE S1. **Travelling wave behaviour for the Fisher-Kolmogorov model (Case 1).** (a) Phase plane for the system (S1.3)-(S1.4). Red circles denote equilibrium points. The numerical solutions of Equation (S1.1) (cyan, solid) and Equation (S1.2) (orange, dashed), in (C, U) co-ordinates, are superimposed. (b) Numerical solution of Equation (S1.1) at $t = 25$ and $t = 50$ (blue). The grey line indicates the initial condition and the arrow indicates the direction of increasing time. (c) The time evolution of $L(t)$. All results are obtained using $P_m^i = P_m^g = 1$, $P_p^i = P_p^g = 0.3$, $P_d^g = P_d^i = 0$, $\delta x = 0.1$, $\delta t = 0.01$, $\epsilon = 10^{-6}$, $v = 0.768$.

Case 1. EQUAL MOTILITY RATES, EQUAL PROLIFERATION RATES, NO AGENT DEATH.

For $P_m^i = P_m^g$, $P_p^i = P_p^g$ and $P_d^i = P_d^g = 0$, there is no co-operative or competitive mechanism. This gives $F(C) = D = D_i = D_g$, and $R(C) = \lambda C(1 - C)$, where $\lambda = \lambda_i = \lambda_g$. Therefore, Equation (2) reduces to the Fisher-Kolmogorov equation [24, 27, 29]

$$\frac{\partial C}{\partial t} = D\frac{\partial^2 C}{\partial x^2} + \lambda C(1 - C). \tag{S1.1}$$

As the source term is non-negative for all physical values of $C \geq 0$, the agent population will always eventually reach the carrying capacity.

The Fisher-Kolmogorov equation has been studied extensively [18–21, 24, 25, 27, 29]. Here we present the key results in the context of examining the long time travelling wave solution. We seek right moving travelling waves in the co-ordinate $z = x - vt$, $-\infty < z < \infty$, where v is a constant wave speed [29]. Transforming Equation (S1.1) into the travelling wave co-ordinate gives

$$D\frac{\mathrm{d}^2 C}{\mathrm{d}z^2} + v\frac{\mathrm{d}C}{\mathrm{d}z} + \lambda C(1 - C) = 0, \quad -\infty < z < \infty. \tag{S1.2}$$

With $U = \mathrm{d}C/\mathrm{d}z$, Equation (S1.2) can be expressed as a system of ordinary differential equations (ODEs),

$$\frac{\mathrm{d}C}{\mathrm{d}z} = U, \tag{S1.3}$$

$$\frac{\mathrm{d}U}{\mathrm{d}z} = -\frac{v}{D}U - \frac{\lambda}{D}C(1 - C). \tag{S1.4}$$

This system has two equilibrium points: $(C, U) = (0, 0)$, and $(C, U) = (1, 0)$. The linear stability of these equilibrium points can be analysed by examining the eigenvalues of the Jacobian at each equilibrium point. At $(0, 0)$ the characteristic equation has solutions $\xi = (-v \pm \sqrt{v^2 - 4\lambda D})/2D$, implying that $(0, 0)$ is a stable node provided that $v > 2\sqrt{\lambda D}$, and a stable spiral (focus) otherwise, as λ and D are both positive. We therefore have a minimum wave speed condition, $v^* = 2\sqrt{\lambda D}$, that must be satisfied otherwise the solution trajectory will enter non-physical regions of the phase plane [29]. The Jacobian of the linearised system at $(1, 0)$ has eigenvalues $\xi = (-v \pm \sqrt{v^2 + 4\lambda D})/2D$, implying that $(1, 0)$ is a saddle point.

The phase plane and associated heteroclinic orbit for Equations (S1.3)-(S1.4) are shown in Figure S1(a). Details of the numerical techniques used to solve Equation (S1.2) and to generate the phase planes are given in the Methods (Main Document). Provided $v \geq 2\sqrt{\lambda D}$ we observe a heteroclinic orbit between $(1, 0)$ and $(0, 0)$. The numerical solution of Equation (S1.2) and the numerical solution of Equation (S1.1), transformed into (C, U) co-ordinates, are superimposed, showing a good match. This result is unsurprising, as Equation (S1.2) is solved using the minimum wave speed, $v = v^* = 2\sqrt{\lambda D}$, and the numerical solution of Equation (S1.1) evolves from a Heaviside initial condition, which is known to approach a travelling wave moving at the minimum wave speed [29]. The numerical solution of Equation (S1.1) at $t = 25$ and $t = 50$ is shown in Figure S1(b), confirming that the waveform does not change with time. To quantify the wave speed we calculate the time evolution of the leading edge, $L(t) = x_f$ such that $C(x_f, t) \approx 1 \times 10^{-4}$. If the solution of Equation (S1.1) forms a travelling wave, $L(t)$ will tend to a straight line with slope v, as $t \to \infty$. In Figure S1(c), we observe that $L(t)$ is approximately linear with slope v, and hence the solution of Equation (S1.1) moves with approximately constant speed at late time. Overall, these features suggest that the solution of Equation (S1.1) is a travelling wave.

Case 2. Different motility rates, equal proliferation rates, no agent death.

If $P_m^i \neq P_m^g$ the governing PDE contains a nonlinear diffusivity term. Since the agent birth rate is independent of agent type and agents do not die, we consider the same source term as for Case 1. Again, there are no competitive or co-operative mechanisms associated with birth/death but it could be either advantageous ($P_m^i > P_m^g$) or disadvantageous ($P_m^i < P_m^g$) for an individual to be isolated from the bulk population, if the goal for the population is to invade unoccupied space. In this parameter regime, Equation (2) simplifies to

$$\frac{\partial C}{\partial t} = \frac{\partial}{\partial x}\left(F(C)\frac{\partial C}{\partial x}\right) + \lambda C(1 - C), \tag{S2.1}$$

where $\lambda = \lambda_i = \lambda_g$ and $F(C) = D_i(1 - 4C + 3C^2) + D_g(4C - 3C^2)$.

$F(C)$ has different properties depending on the choice of P_m^i and P_m^g. To illustrate this, we present the (P_m^i, P_m^g) parameter space in Figure S2(a), and highlight regions of different behaviour of $F(C)$. If $P_m^i > 4P_m^g$, there will be an interval, $1/3 \leq \alpha < C < \beta \leq 1$, centred around $C = 2/3$, where $F(C) < 0$. Specifically, this interval is given by

$$\alpha = \frac{2}{3} - \frac{\sqrt{(P_m^i)^2 - 5P_m^i P_m^g + 4(P_m^g)^2}}{3(P_m^i - P_m^g)} < C < \beta = \frac{2}{3} + \frac{\sqrt{(P_m^i)^2 - 5P_m^i P_m^g + 4(P_m^g)^2}}{3(P_m^i - P_m^g)}. \tag{S2.2}$$

All parameter pairs that result in $F(C) < 0$, which we refer to as *positive-negative-positive*, correspond to the purple region in Figure S2(a), and an example $F(C)$ curve is given in Figure S2(b). Parameter pairs that result in $F(C) < 0$ with $F(1) = 0$ correspond to the black line in Figure S2(a), and an example $F(C)$ curve is given in Figure S2(b). We refer to this type of nonlinear diffusivity function as *capacity-degenerate positive-negative.* It is relevant for us to remark that nonlinear diffusivity functions with negative regions can lead to shocks in the solution of nonlinear diffusion equations without any source term [50, 51]. Therefore, it is instructive to consider whether shock-fronted travelling waves exist with Fisher-Kolmogorov kinetics.

For specific parameter regimes, $F(C)$ is degenerate at $C = 0$, that is, $F(0) = R(0) = 0$. This type of nonlinear diffusivity function, which we refer to as *extinction-degenerate non-negative*, leads to sharp-fronted travelling waves, provided that $F(C) \geq 0$ for $0 \leq C \leq 1$ [40, 46, 47]. For Equation (S2.1), this corresponds to $P_m^i = 0$. The parameter pairs that satisfy this condition correspond to the orange line in Figure S2(a), and a typical $F(C)$ curve is given in Figure S2(b). The special case $P_m^i = P_m^g$ leads to a constant diffusivity, and parameter pairs that satisfy lie along the cyan line in Figure S2(a). A typical $F(C)$ curve for this case is presented in Figure S2(b). For all other parameter pairs $F(C) > 0$, which we refer to as *strictly positive*, and these parameter pairs correspond to the grey region in Figure S2(a), for which an example $F(C)$ curve

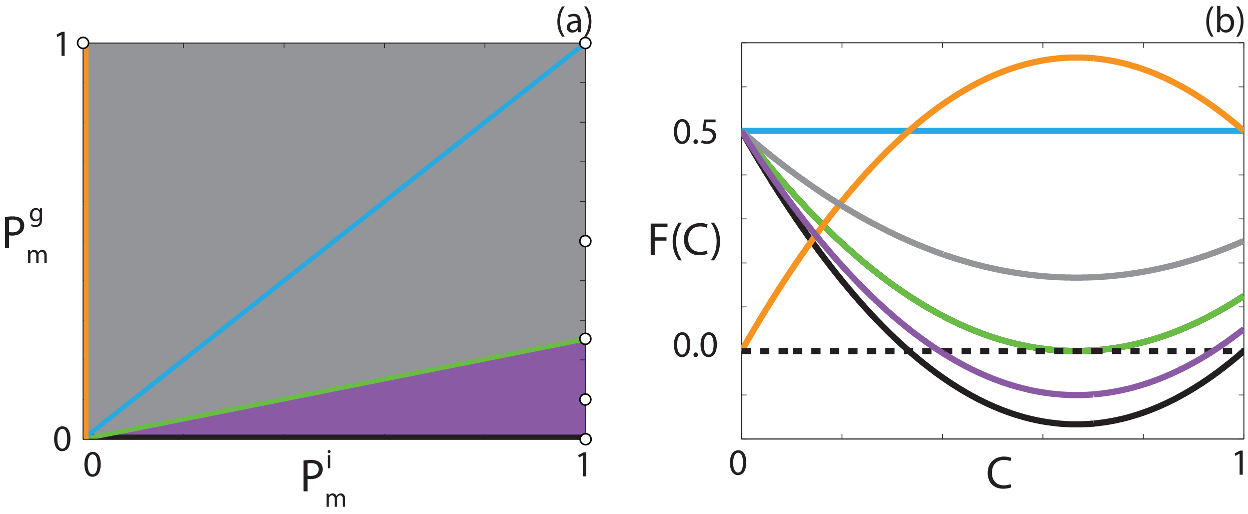

FIGURE S2. **Classification of** $F(C)$. (a) Type of $F(C)$ function for $0 \leq C \leq 1$ for the parameter space $P_m^i \in [0,1]$ and $P_m^g \in [0,1]$. The grey region represents parameter pairs that result in only positive $F(C)$, and the purple region represents parameter pairs that result in negative $F(C)$ for an interval of C. The orange line represents parameter pairs that result in $F(0) = 0$, the green line represents parameter pairs that result in $F(2/3) = 0$, the cyan line represents parameter pairs that result in constant $F(C)$, and the black line represents parameter pairs that result in negative $F(C)$ for an interval of C with $F(1) = 0$. (b) Example $F(C)$ for each region in (a). Positive $F(C)$ (grey), corresponding to $P_m^i = 1$ and $P_m^g = 0.5$. Negative $F(C)$ for an interval of C (purple), corresponding to $P_m^i = 1$ and $P_m^g = 0.1$. Negative $F(C)$ for an interval of C with $F(1) = 0$ (black), corresponding to $P_m^i = 1$ and $P_m^g = 0.1$. $F(0) = 0$ (orange), corresponding to $P_m^i = 0$ and $P_m^g = 1$. $F(2/3) = 0$ (green), corresponding to $P_m^i = 1$ and $P_m^g = 0.25$. Constant $F(C)$ (cyan), corresponding to $P_m^i = 1$ and $P_m^g = 1$. The white circles in (a) denote the parameter pairs used to generate the curves in (b).

is shown in Figure S2(b).

We look for a right moving travelling wave solution of Equation (S2.1) in terms of the co-ordinate $z = x - vt$. Transforming Equation (S2.1) into travelling wave co-ordinates, we obtain

$$v\frac{\mathrm{d}C}{\mathrm{d}z} + F(C)\frac{\mathrm{d}^2C}{\mathrm{d}z^2} + (D_i - D_g)(6C-4)\left(\frac{\mathrm{d}C}{\mathrm{d}z}\right)^2 + \lambda C(1-C) = 0, \quad -\infty < z < \infty. \tag{S2.3}$$

Making the substitution $U = \mathrm{d}C/\mathrm{d}z$ gives

$$\frac{\mathrm{d}C}{\mathrm{d}z} = U, \tag{S2.4}$$

$$\frac{\mathrm{d}U}{\mathrm{d}z} = \frac{-vU - (D_i - D_g)(6C-4)U^2 - \lambda C(1-C)}{F(C)}. \tag{S2.5}$$

We now consider the properties of the travelling wave solutions for several sub-cases within Case 2. Unlike the Fisher-Kolmogorov equation, the minimum wave speed is unknown and hence all phase planes presented in this section are generated with v obtained from the numerical solution of Equation (S2.1) at sufficiently late time.

Sub-case 2.1. **Strictly positive nonlinear diffusivity function.** If $F(C) > 0$ for $0 \leq C \leq 1$, Equations (S2.4)-(S2.5) are not singular for $0 \leq C \leq 1$. Hence the linear analysis performed for Case 1 is valid in

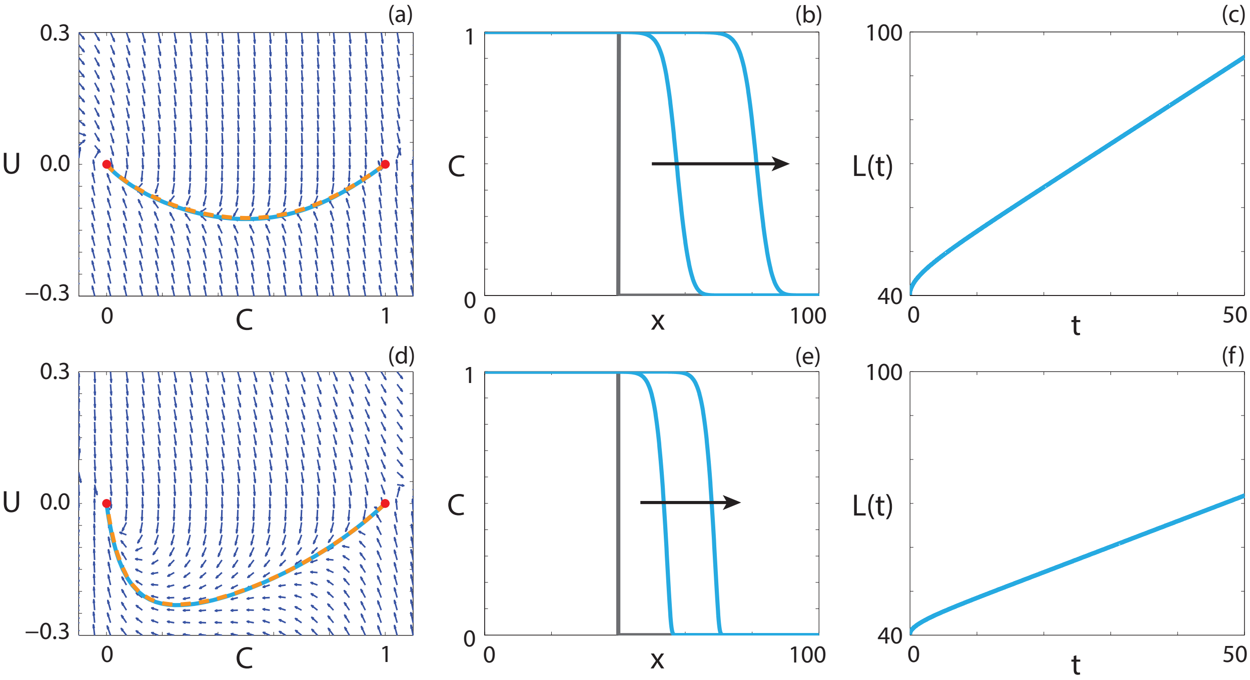

FIGURE S3. **Travelling wave behaviour for Equation** (S2.1) **with strictly positive** $F(C)$ **(Case 2.1)**. (a), (d) Phase plane for the system (S2.4)-(S2.5). Red circles denote equilibrium points. The numerical solutions of Equation (S2.1) (cyan, solid) and Equation (S2.3) (orange, dashed), in (C, U) co-ordinates, are superimposed. (b), (e) Numerical solution of Equation (S2.1) at $t = 25$ and $t = 50$ (blue). The grey lines indicate the initial condition and the arrows indicate the direction of increasing time. (c), (f) The time evolution of the position of the leading edge of the travelling wave solution. All results are obtained using $P_p^i = P_p^g = 0.5$, $P_d^i = P_d^g = 0$, $\delta x = 0.1$, $\delta t = 0.01$, $\epsilon = 10^{-6}$ and (a)-(c) $P_m^i = 1.0$, $P_m^g = 0.5$, $v = 0.992$; (d)-(f) $P_m^i = 0.2$, $P_m^g = 0.8$, $v = 0.584$.

terms of the position and stability of the equilibrium points. The exception is the minimum wave speed condition for the equilibrium point at $(0, 0)$ to be a stable node, which becomes $v > 2\sqrt{\lambda D_i}$, which is always positive [42–44].

Solutions of Equation (S2.1), illustrating travelling wave behaviour for two different $F(C)$ functions are given in Figures S3(a)-(c) and Figures S3(d)-(f), respectively. In both cases the solution trajectory in the phase plane, Figure S3(a) and Figure S3(d), forms a heteroclinic orbit between $(1, 0)$ and $(0, 0)$. Interestingly, the waveform in Figure S3(e), with $P_m^g > P_m^i$, is relatively sharp near $C = 0$. If $P_m^g > P_m^i$, $F(C)$ is concave up with a minimum value of $P_m^i/2$ at $C = 0$, for $0 \leq C \leq 1$, whereas $F(C)$ has a minimum value at $C = 2/3$ for $P_m^i > P_m^g$. This suggests that $F(0)$ has considerable influence on the waveform.

The observed wave speed in Figure S3(a), $v = 0.992$, is close to the predicted minimum wave speed $v^* = 2\sqrt{\lambda D_i} = 1$, whereas the observed wave speed in Figure S3(b), $v = 0.584$, is greater than the predicted minimum wave speed $v^* = 0.447$. To determine whether v^* provides an accurate prediction of the observed wave speed, we calculate the long time numerical solution of Equation (S2.1) and measure v for a suite of P_m^i and P_m^g values. Predicted minimum wave speeds and observed wave speeds are compared in Figure S4. In Figure S4(a), the predicted wave speed is accurate for all P_m^g values with $P_m^i = 0.8$. Interestingly, with $P_m^i = 0.2$, the predicted wave speed is only accurate for $P_m^g \leq 0.4$. Setting $P_m^g = 1$ and varying P_m^i we observe, in Figure S4(b), that the prediction is accurate for $P_m^i \geq 0.5$. Hence, it appears that for $P_m^i \geq 2P_m^g$

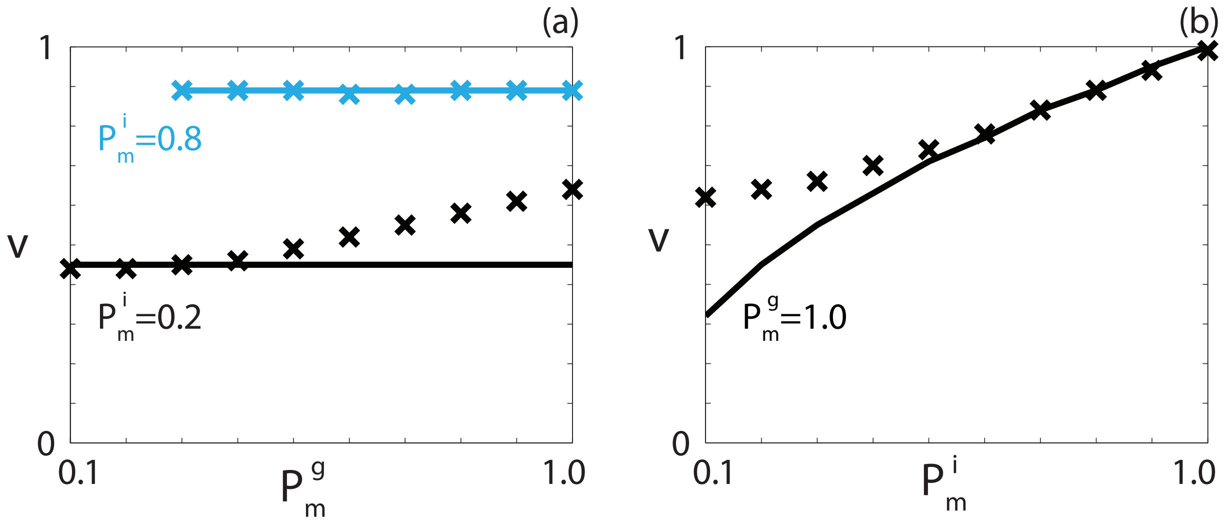

FIGURE S4. **Wave speed comparison for Case 2.1**. Comparison of the minimum wave speed condition (solid) and the observed wave speed at sufficiently late time (crosses) for (a) constant P_m^g and a suite of P_m^i values; (b) constant P_m^i and a suite of P_m^g values. All results are obtained using $P_p^i = P_p^g = 0.5$, $P_d^i = P_d^g = 0$, $\delta x = 0.1$, $\delta t = 0.01$, $\epsilon = 10^{-6}$ and the Heaviside initial condition.

the minimum wave speed condition is accurate. For $P_m^i < 2P_m^g$ the grouped agents may have more successful movement events than the individual agents. Therefore, the dominant contribution to the invasion of the population may be attributed to the grouped agents, which could explain why the minimum wave speed, which depends on P_m^i, does not provide a good estimate of the observed wave speed in these parameter regimes.

Sub-case 2.2. **Extinction-degenerate non-negative nonlinear diffusivity function.** The case where $F(0) = R(0) = 0$, and $F(C) > 0$ for $0 < C \leq 1$, occurs when $P_m^i = 0$. Under these conditions Equations (S2.4)-(S2.5) simplify to

$$\frac{\mathrm{d}C}{\mathrm{d}z} = U, \quad \text{(S2.6)}$$

$$\frac{\mathrm{d}U}{\mathrm{d}z} = \frac{1}{D_g(4C-3C^2)}\Big(-vU + D_g(6C-4)U^2 - \lambda C(1-C)\Big), \quad -\infty < z < \infty. \quad \text{(S2.7)}$$

Note that Equation (S2.7) is singular at $C = 0$ and, furthermore, that $R(0) = 0$. Hence we apply a stretching transformation

$$\zeta = \int_0^z \frac{1}{D_g(4C(s) - 3C(s)^2)}\,\mathrm{d}s, \quad \text{(S2.8)}$$

to remove the singularity, which gives

$$\frac{\mathrm{d}C}{\mathrm{d}\zeta} = D_g U(4C - 3C^2), \quad \text{(S2.9)}$$

$$\frac{\mathrm{d}U}{\mathrm{d}\zeta} = -vU + D_g(6C-4)U^2 - \lambda C(1-C), \ \zeta \geq 0. \quad \text{(S2.10)}$$

Equations (S2.9)-(S2.10) have equilibrium points at $(C,U) = (1,0)$, $(C,U) = (0,0)$ and $(C,U) = (0, -v/4D_g)$. The additional equilibrium point in the transformed system corresponds to a solution trajectory approaching $C = 0$ with a non-zero slope. Performing linear analysis to determine the eigenvalues of the Jacobian at the steady states, we find that the characteristic equation at $(0,0)$ has solutions $\xi = 0$ and $\xi = v$, implying that $(0,0)$ is an improper node. Sánchez-Garduño and Maini [40] investigate the stability of this equilibrium

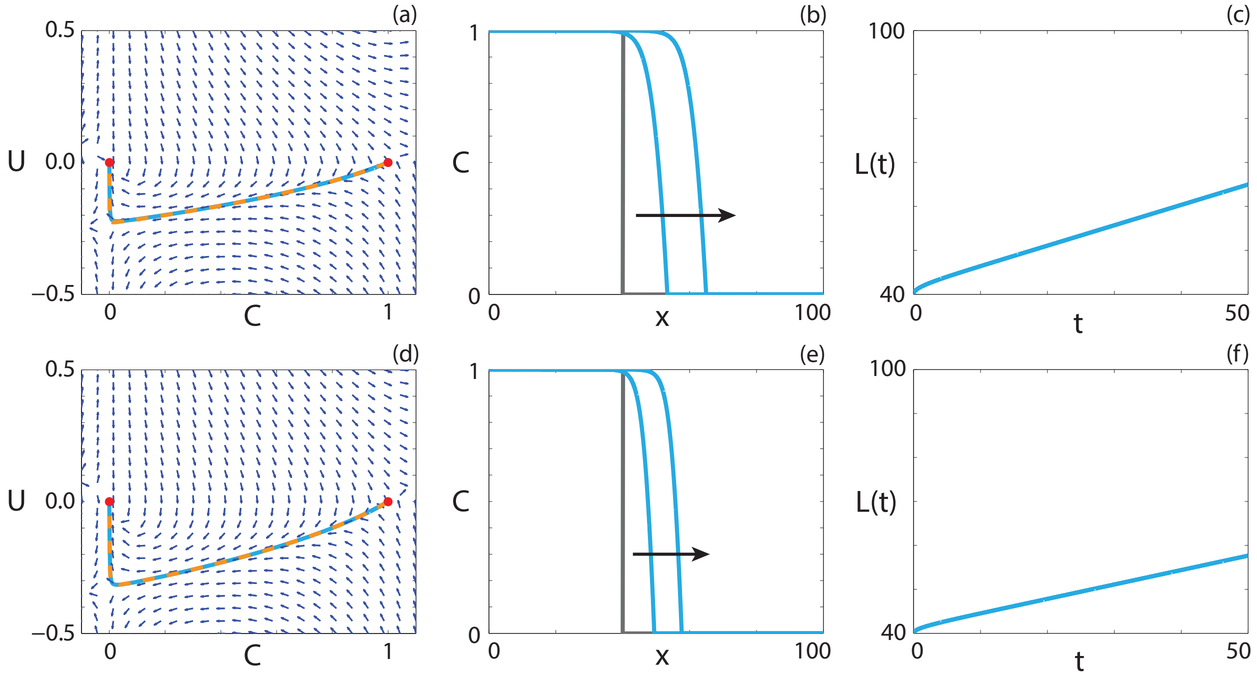

FIGURE S5. **Travelling wave behaviour for Equation** (S2.1) **with extinction-degenerate non-negative** $F(C)$ **(Case 2.2)**. (a), (d) Phase plane for the system (S2.6)-(S2.7). Red circles denote equilibrium points. The numerical solutions of Equations (S2.1) (cyan, solid) and (S2.3) (orange, dashed), in (C, U) co-ordinates, are superimposed. (b), (e) Numerical solution of Equation (S2.1) at $t = 25$ and $t = 50$. The grey lines indicate the initial condition and the arrows indicate the direction of increasing time. (c), (f) The time evolution of the position of the leading edge of the travelling wave solution. All results are obtained using $P_m^i = 0$, $P_p^i = P_p^g = 0.3$, $P_d^g = P_d^i = 0$, $\delta x = 0.01$, $\delta t = 0.01$, $\epsilon = 10^{-6}$ and (a)-(c) $P_m^g = 1.0$, $v = 0.463$; (d)-(f) $P_m^g = 0.5$, $v = 0.328$.

point and find that the equilibrium point is a saddle node. The characteristic equation at $(1, 0)$ has solutions $\xi = (-v \pm \sqrt{v^2 + 4\lambda D_g})/2$, implying that $(1, 0)$ is a saddle point. Finally the characteristic equation of the equilibrium point at $(0, -v/4D_g)$ has eigenvalues $\xi = \pm v$, implying that $(0, -v/4D_g)$ is a saddle point. A critical value v^* exists such that $v < v^*$ results in no travelling wave solution, $v = v^*$ results in a sharp-fronted travelling wave and $v > v^*$ results in a classic (smooth) travelling wave [40].

Numerical solutions illustrating travelling wave behaviour for Equation (S2.1) with $P_m^i = 0$ are given in Figure S5. In the phase plane for both cases, Figure S5(a) and Figure S5(d), the solution trajectory tends to the origin with $\mathrm{d}U/\mathrm{d}C$ large and negative. The corresponding numerical solutions of Equation (S2.1), presented in Figure S5(b) and Figure S5(e), approach a travelling wave solution with a sharp front near $C = 0$. This result is expected as the Heaviside initial condition results in the minimum wave speed that, for a degenerate diffusivity function, results in a sharp-fronted wave [46].

Sub-case 2.3. **Positive-negative-positive nonlinear diffusivity function.** In order for $F(C)$ to change sign twice, that is, $F(C) < 0$ for $1/3 \leq \alpha < C < \beta \leq 1$ and $F(C) \geq 0$ otherwise for $0 \leq C \leq 1$, the parameters must lie within the purple region in Figure S2(a). In this situation, Equations (S2.4)-(S2.5) are undefined at $C = \alpha$ and $C = \beta$, and these singularities cannot be removed using a stretching transformation since $R(\alpha) \neq 0$ and $R(\beta) \neq 0$. However, it is possible for $\mathrm{d}U/\mathrm{d}z$ to be finite at $C = \alpha$ and $C = \beta$ if U_α and

U_β exist such that

$$\lim_{C\to\alpha}\left[\frac{-vU_\alpha-(D_i-D_g)(6C-4)U_\alpha^2-\lambda C(1-C)}{D_i(1-4C+3C^2)+D_g(4C-3C^2)}\right], \tag{S2.11}$$

$$\lim_{C\to\beta}\left[\frac{-vU_\beta-(D_i-D_g)(6C-4)U_\beta^2-\lambda C(1-C)}{D_i(1-4C+3C^2)+D_g(4C-3C^2)}\right], \tag{S2.12}$$

are both finite. This requires the numerator in the expressions (S2.11)-(S2.12) vanish at $C=\alpha$ and $C=\beta$, respectively. As such, U_α and U_β are obtained by solving the system

$$0=-vU_\alpha-(D_i-D_g)(6\alpha-4)U_\alpha^2-\lambda\alpha(1-\alpha), \tag{S2.13}$$

$$0=-vU_\beta-(D_i-D_g)(6\beta-4)U_\beta^2-\lambda\beta(1-\beta), \tag{S2.14}$$

resulting in $U_\alpha=-(v\pm\sqrt{v^2-4F'(\alpha)R(\alpha)})/2F'(\alpha)$ and $U_\beta=-(v\pm\sqrt{v^2-4F'(\beta)R(\beta)})/2F'(\beta)$. We note that, as $R(C)\geq 0$ for $0\leq C\leq 1$, and $F'(\alpha)\leq 0$ for all possible α values, U_α will be real-valued. Subsequently, we have a wave speed condition that $v\geq 2\sqrt{F'(\beta)R(\beta)}$, as $F'(\beta)\geq 0$ for all possible β values. Ferracuti *et al.* [36] prove that the minimum wave speed, v^*, is greater than a threshold value, which in turn is greater than $\max\{R'(0)F(0),F'(\beta)R(\beta)\}$. Therefore, U_β will also always be real-valued.

Applying L'Hopital's Rule to Equation (S2.5), we obtain

$$\lim_{C\to\alpha}\left.\frac{\mathrm{d}U}{\mathrm{d}z}\right|_{U=U_\alpha}=\lim_{C\to\alpha}\left[\frac{6(D_i-D_g)U_\alpha^2+\lambda(1-2C)}{(D_g-D_i)(6C-4)}\right], \tag{S2.15}$$

$$\lim_{C\to\beta}\left.\frac{\mathrm{d}U}{\mathrm{d}z}\right|_{U=U_\beta}=\lim_{C\to\beta}\left[\frac{6(D_i-D_g)U_\beta^2+\lambda(1-2C)}{(D_g-D_i)(6C-4)}\right], \tag{S2.16}$$

which are finite provided that $\alpha\neq 2/3$ and $\beta\neq 2/3$. For the system of Equations (S2.4)-(S2.5), we have two straight lines in the phase plane where $\mathrm{d}U/\mathrm{d}z$ is infinite, at $C=\alpha$ and $C=\beta$. These kind of lines have previously been called *walls of singularities* for hyperbolic models related to chemotactic and haptotactic invasion [52]. For a smooth solution trajectory to exist between two equilibrium points on opposite sides of the wall of singularities, we require that the trajectory passes through the wall of singularities. This implies that the solution trajectory must pass through the wall of singularities at the special points, (α,U_α) and (β,U_β), known as holes in the wall [52,53]. Otherwise, a smooth heteroclinic orbit between $(1,0)$ and $(0,0)$ cannot exist, as $\lim_{C\to\alpha}|U|\to\infty$ and $\lim_{C\to\beta}|U|\to\infty$. As U_α and U_β are real valued and the limits in Equations (20)-(21) are finite, the holes in the wall always exist for Fisher kinetics.

Ferracuti *et al.* [36] prove that travelling wave solutions exist for reaction-diffusion equations with positive-negative-positive $F(C)$ and Fisher kinetics, however travelling wave profiles arising from the PDE are not presented. An upper bound on the minimum wave speed is stated as [36]

$$v^*=\max\{v_1,v_2,v_3\}, \tag{S2.17}$$

where

$$F'(0)R(0)+F(0)R'(0)\leq\frac{v_1^2}{4}\leq\sup_{C\in(0,\alpha]}\left[\frac{F(C)R(C)}{C}\right], \tag{S2.18}$$

$$F'(\beta)R(\beta)+F(\beta)R'(\beta)\leq\frac{v_2^2}{4}\leq\sup_{C\in[\alpha,\beta)}\left[\frac{F(C)R(C)}{C-\beta}\right], \tag{S2.19}$$

$$F'(\beta)R(\beta)+F(\beta)R'(\beta)\leq\frac{v_3^2}{4}\leq\sup_{C\in(\beta,1]}\left[\frac{F(C)R(C)}{C-\beta}\right], \tag{S2.20}$$

and the prime denotes ordinary differentiation with respect to C. Numerical solutions of Equation (S2.1) with $P_m^i>4P_m^g$ are presented in Figure S6. We superimpose the numerical solution of Equation (S2.1) in (C,U) co-ordinates on the phase plane for the system (S2.4)-(S2.5) in Figures S6(a) and S6(d). The numerical solution forms a heteroclinic orbit between $(1,0)$ and $(0,0)$ in both cases, and passes through the holes

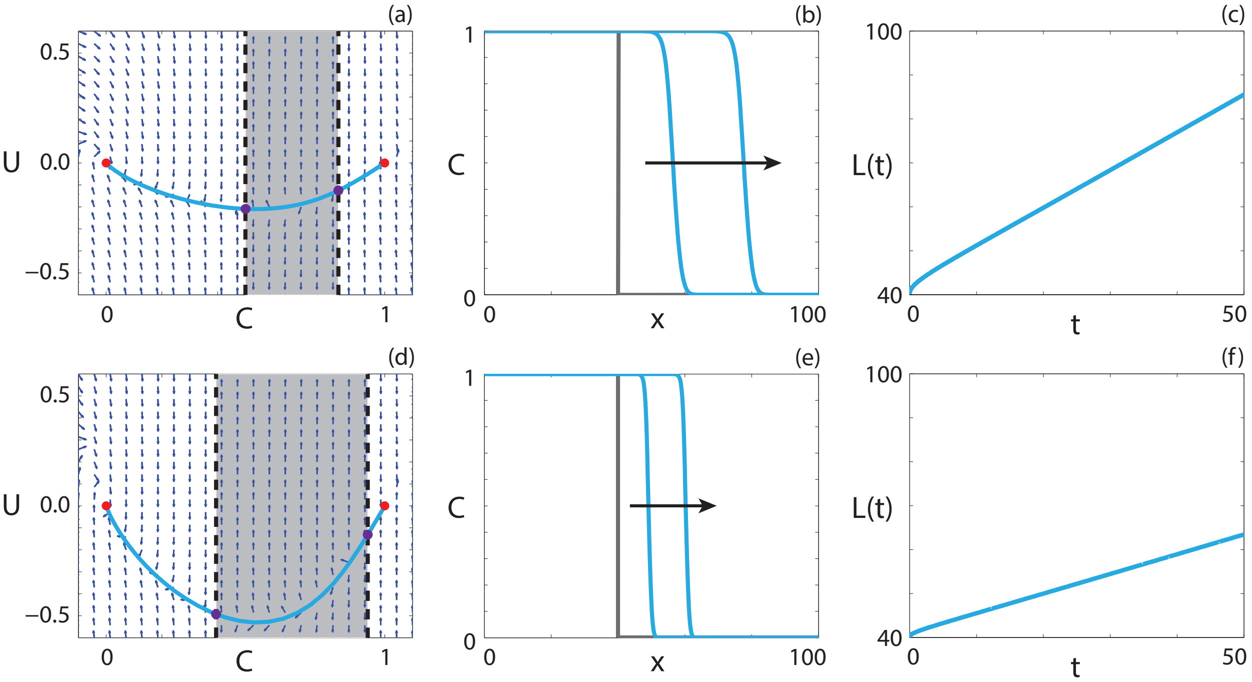

Figure S6. **Travelling wave behaviour for Equation** (S2.1) **with positive-negative-positive** $F(C)$ **(Case 2.3)**. (a), (d) Phase plane for the system (S2.4)-(S2.5) with the numerical solution of Equation (S2.1), in (C, U) co-ordinates, superimposed. The grey region corresponds to values of C where $F(C) < 0$. The dashed black lines denote a wall of singularities. Red circles correspond to equilibrium points and purple circles correspond to holes in the wall. (b), (e) Numerical solution of Equation (S2.1) at $t = 100$ and $t = 200$. The grey lines indicate the initial condition and the arrows indicate the direction of increasing time. (c), (f) The time evolution of the position of the leading edge of the travelling wave solution, $L(t)$. All results are obtained using $P_d^i = P_d^g = 0$, $\delta x = 0.01$, $\delta t = 0.01$, $\epsilon = 10^{-6}$ and (a)-(c) $P_m^i = 0.5$, $P_m^g = 0.1$, $P_p^i = P_p^g = 0.75$, $v = 0.864$; (d)-(f) $P_m^i = 0.1$, $P_m^g = 0.01$, $P_p^i = P_p^g = 1.0$, $v = 0.448$.

in the wall of singularities, denoted using purple circles. Continuum models with negative diffusivity and no source terms have been relatively well studied, and exhibit shock behaviour across the region of negative diffusion [50, 51]. Interestingly, our solution does not include a shock and is instead smooth through the region of negative diffusion.

Numerical solutions of Equation (S2.1) are presented in Figures S6(b) and S6(e), which appear to take the form of travelling waves. The observed wave speeds, $v = 0.864$ and $v = 0.456$, in Figure S6(c) and Figure S6(f), respectively, are well approximated by the upper bound on the minimum wave speed presented by Ferracuti *et al.* [36]. The bound provides values for the minimum wave speed of $v^* = 0.866$ and $v^* = 0.447$, respectively. We might expect that the observed wave speeds correspond to the minimum wave speeds since the initial conditions for the numerical solutions are given by a Heaviside initial condition.

The observed wave speed, obtained from the long time numerical solutions of Equation (S2.1), and the upper bound on the minimum wave speed, given by Equation (S2.17), are shown in Figure S7 for a suite of P_p values and two positive-negative-positive $F(C)$ functions. In all cases the bound provides an accurate prediction of the observed wave speed.

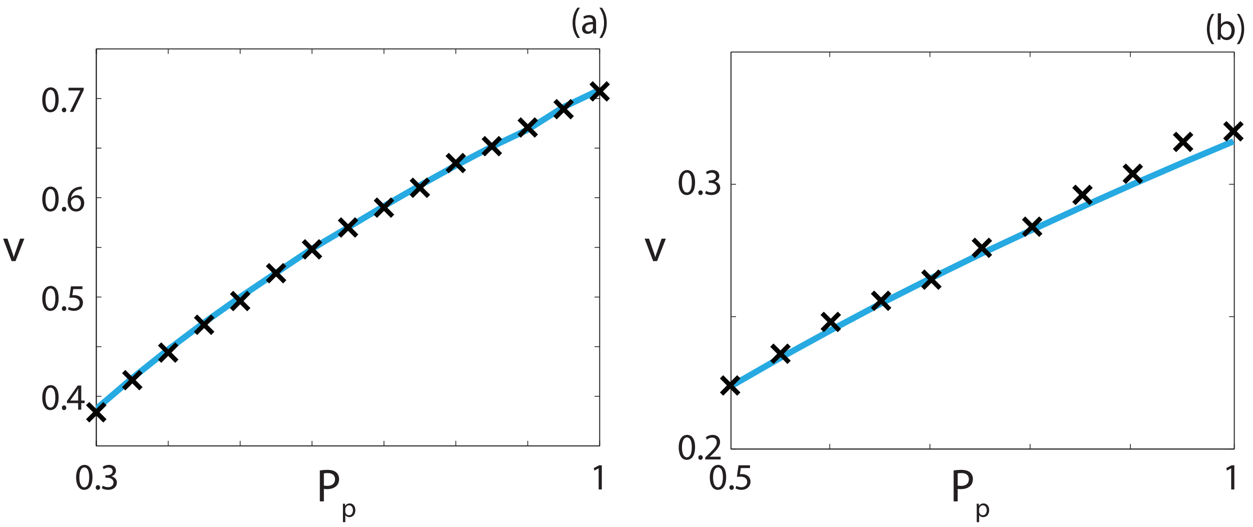

FIGURE S7. **Wave speed comparison for Case 2.3**. Comparison of the observed wave speed and the upper bound of the minimum wave speed obtained from the relationship in Equation (S2.17) for a suite of P_p values. All results are obtained using $P_d^i = P_d^g = 0$, $\delta x = 0.01$, $\delta t = 0.01$, $\epsilon = 10^{-6}$, and (a) $P_m^i = 0.25$, $P_m^g = 0.05$; (b) $P_m^i = 0.05$, $P_m^g = 0.005$, and the Heaviside initial condition. In all cases Equation (S2.18) provided the estimate of the minimum wave speed. Crosses correspond to the observed wave speed and the solid line corresponds to the upper bound of the wave speed.

Sub-case 2.4. **Capacity-degenerate positive-negative nonlinear diffusivity function.** For the special case where $P_m^g = 0$, $F(1) = 0$. As $F(C)$ is degenerate at $C = 1$, it is intuitive to expect there could be sharp-fronted travelling wave solutions, with the sharp front near $C = 1$, similar to the results in Figure S5. However, unlike for the parameter regimes in Figure S5, we have an interval $1/3 < C < 1$ where $F(C) < 0$. To determine whether this negative diffusivity influences the presence of sharp fronts, we follow the approach of Maini *et al.* [38], who show that the existence of travelling waves for reaction-diffusion equations with capacity-degenerate positive-negative $F(C)$ can be determined by considering the existence of travelling waves for

$$\frac{\partial C}{\partial \hat{t}} = \frac{\partial^2 C}{\partial x^2} + F(C)R(C), \ \hat{t} \geq 0. \tag{S2.21}$$

The restriction on $\hat{t}$ implies that $F(C) > 0$. As $F(C) < 0$ for $1/3 < C < 1$, Equation (S2.21) is only equivalent to Equation (S2.1) for $0 \leq C \leq 1/3$. For $1/3 \leq C \leq 1$, Equation (S2.1) is equivalent to

$$\frac{\partial C}{\partial \hat{t}} = \frac{\partial^2 C}{\partial x^2} + \hat{F}(C)\hat{R}(C), \ \hat{t} \geq 0, \tag{S2.22}$$

where $\hat{F}(C) = -F(1-C)$ and $\hat{R}(C) = R(1-C)$ [38]. Equations (S2.21)-(S2.22) have minimum travelling wave speeds v_0^* and v_1^*, respectively. Maini *et al.* [38] prove that sharp fronts in the travelling wave near $C = 1$ only exist if $F(1) = 0$ and $v_1^* < v_0^*$. The first condition is obviously satisfied, while the second can be determined through linear analysis of Equations (S2.21)-(S2.22) in travelling wave co-ordinates. Both equations have minimum wave speed conditions, $v_0^* = v_1^* = 2\sqrt{\lambda D_i}$, to obtain physically-relevant heteroclinic orbits, and hence travelling wave solutions with a sharp region near $C = 1$ do not exist.

Travelling wave behaviour for a parameter regime with $F(1) = 0$ is shown in Figure S8. The equilibrium point at $(1, 0)$ is also a hole in the wall. The solution trajectory forms a heteroclinic orbit between $(1, 0)$ and $(0, 0)$, and moves through the region of C where $F(C) < 0$. Although $F(1) = 0$, we do not observe a solution trajectory corresponding to a sharp front, as we observed in Figure S5, where $F(0) = 0$. This result is consistent with the analysis of Maini *et al.* [38]. The numerical solution of Equation (S8), presented in

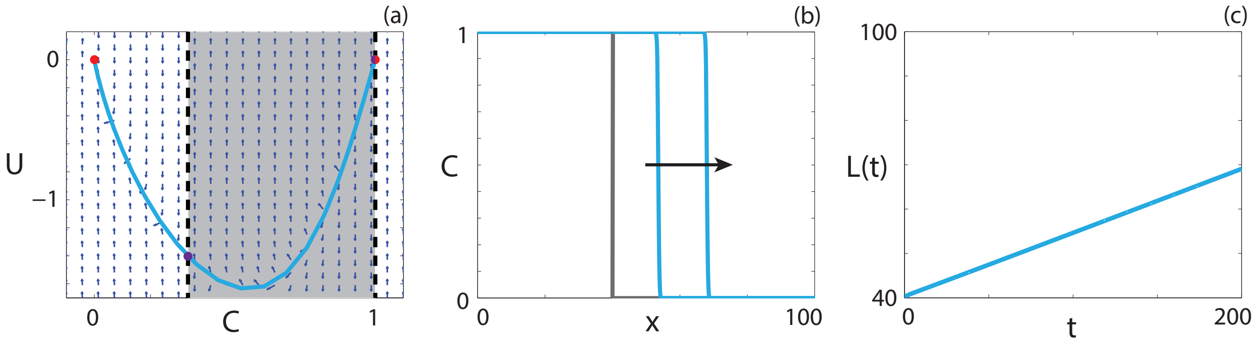

FIGURE S8. **Travelling wave behaviour for Equation** (S2.1) **with capacity-degenerate positive-negative** $F(C)$ **(Case 2.4).** (a) Phase plane for the system (S2.4)-(S2.5) with the numerical solution of Equation (S2.1), in (C, U) co-ordinates, superimposed. The grey region corresponds to values of C where $F(C) < 0$. The dashed black lines denote a wall of singularities. Red circles correspond to equilibrium points and purple circles correspond to holes in the wall. (b) Numerical solution of Equation (S2.1) at $t = 100$ and $t = 200$. The grey lines indicate the initial condition and the arrow indicates the direction of increasing time. (c) The time evolution of the position of the leading edge of the travelling wave solution, $L(t)$. All results are obtained using $P_m^i = 0.01$, $P_m^g = 0$, $P_p^i = P_p^g = 1.0$, $P_d^i = P_d^g = 0$, $\delta x = 0.01$, $\delta t = 0.01$, $\epsilon = 10^{-6}$, $v = 0.1433$.

Figure S8(b), has a relatively steep front but is not sharp near $C = 1$. As $L(t)$, presented in Figure S8(c), becomes linear as t increases and the waveform in Figure S8(b) are consistent, the numerical solution of Equation (S2.1) with $F(1) = 0$ appears to form a classic travelling wave.

Case 3. EQUAL MOTILITY RATES, EQUAL PROLIFERATION RATES, EQUAL DEATH RATES.

For $P_d^i = P_d^g > 0$, with $P_m^i = P_m^g$ and $P_p^i = P_p^g$, there are no competitive or co-operative mechanisms. In this case, Equation (2) becomes

$$\frac{\partial C}{\partial t} = D\frac{\partial^2 C}{\partial x^2} + \lambda C(1-C) - KC, \tag{S3.1}$$

where $D = D_i = D_g$, $\lambda = \lambda_i = \lambda_g$ and $K = K_i = K_g$. The corresponding ODE in travelling wave co-ordinates is

$$D\frac{\mathrm{d}^2 C}{\mathrm{d}z^2} + v\frac{\mathrm{d}C}{\mathrm{d}z} + \lambda C(1-C) - KC = 0, \ -\infty < z < \infty, \tag{S3.2}$$

and, with $U = \mathrm{d}C/\mathrm{d}z$, we obtain

$$\frac{\mathrm{d}C}{\mathrm{d}z} = U, \tag{S3.3}$$

$$\frac{\mathrm{d}U}{\mathrm{d}z} = -\frac{v}{D}U - \frac{\lambda}{D}C(1-C) + \frac{K}{D}C. \tag{S3.4}$$

The source term in Equation (S3.1) is non-positive for all relevant C values if $K > \lambda$, and negative for $C > (\lambda - K)/\lambda$ otherwise. Hence the population will never reach the original carrying capacity of unity. The new carrying capacity can be determined by considering the zeros of the source term, which occur at $C = 0$ and $C = (\lambda - K)/\lambda$. Introducing a new variable, $\overline{C} = \lambda C/(\lambda - K)$, and rewriting Equation (S3.1) in terms of $\overline{C}$ we obtain

$$\frac{\partial \overline{C}}{\partial t} = D\frac{\partial^2 \overline{C}}{\partial x^2} + (\lambda - K)\overline{C}(1-\overline{C}). \tag{S3.5}$$

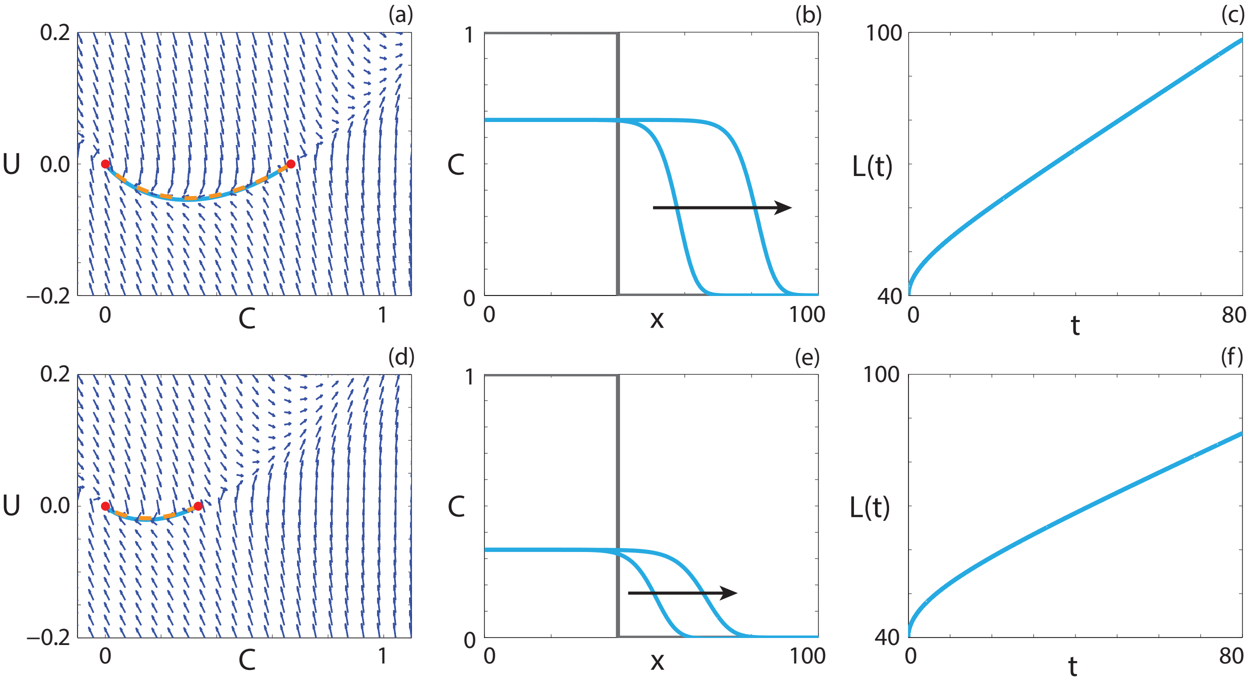

Figure S9. **Travelling wave behaviour for the scaled Fisher-Kolmogorov model (Case 3)**. (a), (d) Phase plane for the system (S3.3)-(S3.4). Red circles denote equilibrium points. The numerical solutions of Equations (S3.1) (cyan, solid) and (S3.2) (orange, dashed), in (C, U) co-ordinates, are superimposed. (b), (e) Numerical solution of Equation (S3.1) at $t = 40$ and $t = 80$. The grey lines indicate the initial condition and the arrows indicate the direction of increasing time. (c), (f) The time evolution of the position of the leading edge of the travelling wave solution. All results are obtained using $P_m^i = P_m^g = 1$, $P_p^i = P_p^g = 0.3$, $\delta x = 0.1$, $\delta t = 0.01$, $\epsilon = 10^{-6}$ and (a)-(c) $P_d^g = P_d^i = 0.1$, $v = 0.615$; (d)-(f) $P_d^g = P_d^i = 0.2$, $v = 0.445$.

Equation (S3.5) is the Fisher-Kolmogorov equation in terms of the new variable, $\overline{C}$, with an intrinsic growth rate $(\lambda - K)$. As such, the analysis performed for Case 1 is applicable here and we obtain information about the stability of the equilibrium points, as well as the minimum wave speed required for physically meaningful travelling wave solutions. If $\lambda > K$, the minimum wave speed is $v^* = 2\sqrt{(\lambda - K)D}$. If $K > \lambda$, there is only one physically relevant equilibrium point, $C = 0$, and hence the population will tend to extinction and travelling wave solutions do not exist.

Travelling wave behaviour for two parameter regimes with $\lambda > K$ are illustrated in Figure S9. The phase plane for $K = 0.1$, presented in Figure S9(a), displays qualitatively similar behaviour to the phase plane for $K = 0.2$, presented in Figure S9(d). Unsurprisingly, the unstable equilibrium point moves closer to zero as K approaches λ. The numerical solutions of Equation (S3.1), presented in Figures S9(b) and S9(e), have significantly different densities behind the wave fronts. However, both travelling wave fronts represent heteroclinic orbits between $(C, U) = ((\lambda - K)/\lambda, 0)$ and $(C, U) = (0, 0)$. Interestingly, the two travelling wave fronts have approximately the same support, even though the waveform is significantly shallower for the case with $K = 0.2$. Results in Figures S9(c) and (f) show that both solutions approach travelling waves as t increases, and that increasing the death rate reduces the wave speed.

Case 4. DIFFERENT MOTILITY RATES, EQUAL PROLIFERATION RATES, EQUAL DEATH RATES.

For $P_p^i = P_p^g$, $P_d^i = P_d^g$ and $P_m^i \neq P_m^g$, the co-operative or competitive mechanism arises due to the difference in the rate of motility. In this case, the governing PDE is

$$\frac{\partial C}{\partial t} = \frac{\partial}{\partial x}\left(F(C)\frac{\partial C}{\partial x}\right) + \lambda C(1-C) - KC, \tag{S4.1}$$

where $F(C) = D_i(1-4C+3C^2) + D_g(4C-3C^2)$, $\lambda = \lambda_i = \lambda_g$ and $K = K_i = K_g$. Equation (S4.1) corresponds to

$$v\frac{\mathrm{d}C}{\mathrm{d}z} + F(C)\frac{\mathrm{d}^2C}{\mathrm{d}z^2} + (D_i - D_g)(6C-4)\left(\frac{\mathrm{d}C}{\mathrm{d}z}\right)^2 + \lambda C(1-C) - KC = 0, \ -\infty < z < \infty, \tag{S4.2}$$

in travelling wave co-ordinates, and with the substitution $U = \mathrm{d}C/\mathrm{d}z$, we obtain

$$\frac{\mathrm{d}C}{\mathrm{d}z} = U, \tag{S4.3}$$

$$\frac{\mathrm{d}U}{\mathrm{d}z} = \frac{-vU - (D_i - D_g)(6C-4)U^2 - \lambda C(1-C) + KC}{F(C)}. \tag{S4.4}$$

The system of Equations (S4.3)-(S4.4) has equilibrium points $(C, U) = (0, 0)$ and $(C, U) = (S, 0)$, where $S = (\lambda - K)/\lambda$. Increasing K causes a decrease in the carrying capacity, S. If $K > \lambda$, the non-zero equilibrium point occurs at a negative C value and hence only one physically relevant equilibrium point exists, implying that the population will become extinct. Hence we only investigate the behaviour of parameter regimes where $\lambda > K$.

We introduce the variable $\overline{C} = C/S$ such that the agent density is scaled by the carrying capacity and, subsequently, the zeros of the source term occur at $\overline{C} = 0$ and $\overline{C} = 1$. This approach allows us to repeat the analysis for Case 2 with a different $F(C)$. We transform Equation (S4.1) in terms of $\overline{C}$ to obtain

$$\frac{\partial \overline{C}}{\partial t} = \frac{\partial}{\partial x}\left(F(S\overline{C})\frac{\partial \overline{C}}{\partial x}\right) + (\lambda - K)\overline{C}(1-\overline{C}). \tag{S4.5}$$

If we define $\overline{U} = \mathrm{d}\overline{C}/\mathrm{d}z$, Equation (S4.5) corresponds to the system,

$$\frac{\mathrm{d}\overline{C}}{\mathrm{d}z} = \overline{U}, \tag{S4.6}$$

$$\frac{\mathrm{d}\overline{U}}{\mathrm{d}z} = \frac{-v\overline{U} - (D_i - D_g)S(6S\overline{C}-4)\overline{U}^2 - (\lambda - K)\overline{C}(1-\overline{C})}{F(S\overline{C})}, \ -\infty < z < \infty. \tag{S4.7}$$

The transformed nonlinear diffusivity function

$$F_s(\overline{C}) = F(S\overline{C}) = D_i(1 - 4S\overline{C} + 3(S\overline{C})^2) + D_g(4S\overline{C} - 3(S\overline{C})^2), \tag{S4.8}$$

has different properties depending on S, D_i and D_g. To highlight this, Figure S10 shows the (P_m^i, P_m^g) parameter space for three different S values and the qualitative behaviour of the corresponding $F_s(\overline{C})$ function. For $S = 1$, presented in Figure S10(a), we recover the nonlinear diffusivity function examined for Case 2, where for $P_m^i > 4P_m^g$, denoted in purple, there is an interval $\alpha < \overline{C} < \beta$, $\alpha < \beta < 1$, where $F_s(\overline{C}) < 0$. Decreasing S to 0.9, presented in Figure S10(b), we observe that the purple region again occurs for $P_m^i > 4P_m^g$. However, if $P_m^g < 0.145P_m^i$, highlighted in red, $F_s(\overline{C}) < 0$ for $\omega < \overline{C} \leq 1$, and hence $F_s(\overline{C})$ has only one zero in $0 \leq \overline{C} \leq 1$. This type of nonlinear diffusivity function is not observed for Case 2 and we refer to it as *positive-negative*. Specifically, this behaviour occurs when $(16-(6S-4)^2)P_m^g < (4-(6S-4)^2)P_m^i$ and $P_m^i > 4P_m^g$. Furthermore, this implies that for $S < 2/3$ there are no (P_m^i, P_m^g) values that correspond to positive-negative-positive $F_s(\overline{C})$. An example of this (P_m^i, P_m^g) parameter space is shown in Figure S10(c). For $S < 1/3$, $F_s(\overline{C}) \geq 0$ for $0 \leq \overline{C} \leq 1$. Parameter pairs that correspond to extinction-degenerate non-negative $F_s(\overline{C})$ (orange) and constant $F_s(\overline{C})$ (cyan) exist for all S values.

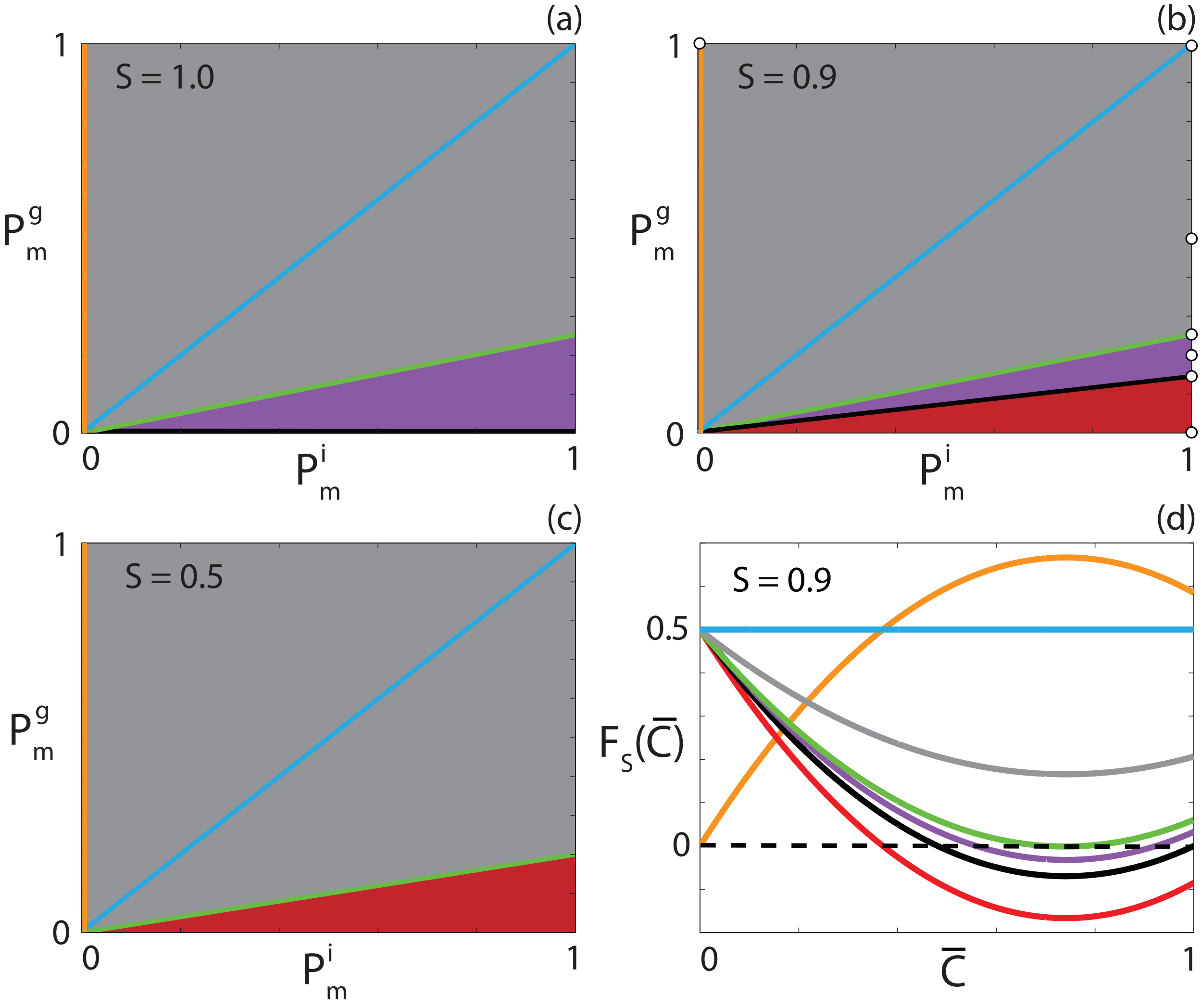

FIGURE S10. **Classification of** $F_s(\overline{C})$ for different carrying capacity densities. (a)-(c) Type of $F_s(\overline{C})$ function for $0 \leq \overline{C} \leq 1$ for the parameter space $P_m^i \in [0,1]$ and $P_m^g \in [0,1]$ with (a) $S = 1.0$; (b) $S = 0.9$; (c) $S = 0.5$. Grey regions correspond to parameter pairs that result in strictly positive $F_s(\overline{C})$, purple regions correspond to parameter pairs that result in positive-negative-positive $F_s(\overline{C})$ and red regions correspond to parameter pairs that result in positive-negative $F_s(\overline{C})$. Cyan, orange and black lines correspond to constant, extinction-degenerate non-negative and capacity-degenerate positive-negative $F_s(\overline{C})$ curves, respectively. (d) Example $F_s(\overline{C})$ for each region in (b). The white circles in (b) denote the parameter pairs used to generate the curves in (d).

Sub-case 4.1. **Strictly positive nonlinear diffusivity function.** If $F_s(\overline{C}) > 0$ for $0 \leq \overline{C} \leq 1$, linear analysis leads to a minimum wave speed $v^* = 2\sqrt{(\lambda - K)D_i}$ for the equilibrium point at $(0,0)$ to be stable. Intuitively, the wave speed is positive provided that the rate of birth is greater than the rate of death.

Travelling wave behaviour for Equation (S4.1) with strictly positive $F_s(\overline{C})$ and $S = 0.5$ is shown in Figures S11(a)-(c). Similar to the strictly positive $F(C)$ considered in Case 2.1, there is a heteroclinic orbit between the two equilibrium points, implying that the solution of Equation (S4.1) forms a travelling wave. Intuitively,

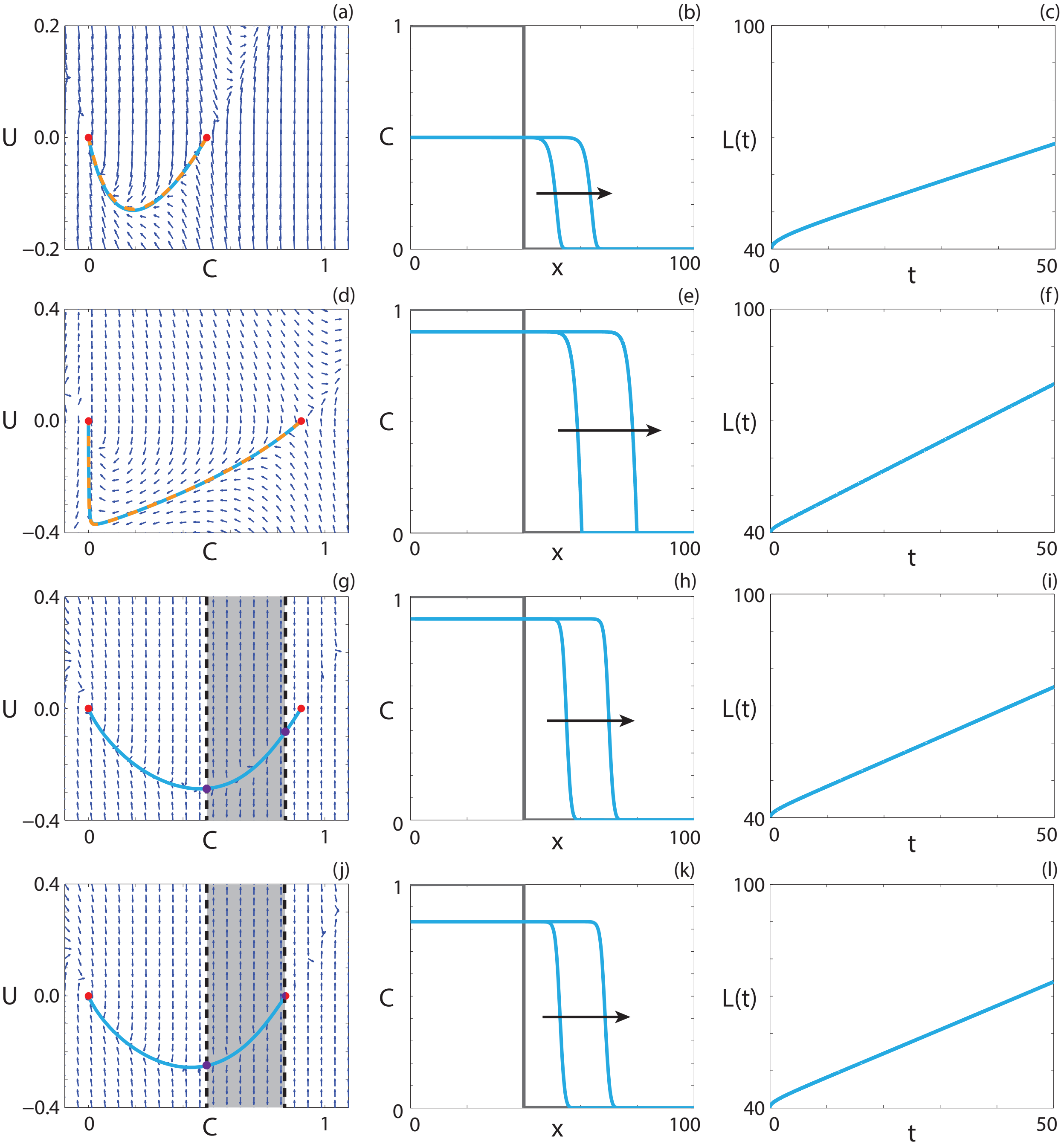

Figure S11. **Travelling wave behaviour for Equation** (S4.1) **for various** $F_s(\overline{C})$. We consider (a)-(c) strictly positive $F_s(\overline{C})$ (Case 4.1), (d)-(f) extinction-degenerate non-negative $F_s(\overline{C})$ (Case 4.2), (g)-(i) positive-negative-positive $F(S\overline{C})$ (Case 4.3), and (j)-(l) capacity-degenerate positive-negative $F_s(\overline{C})$ (Case 4.4). (a), (d), (g), (j) Phase plane for the system (S4.3)-(S4.4) with the numerical solution of Equations (S4.1) (cyan, solid) and (S4.2) (orange, dashed), in (C, U) co-ordinates, superimposed. The grey regions correspond to values of $\overline{C}$ where $F_s(\overline{C}) < 0$. The dashed black lines denote a wall of singularities. Red circles correspond to equilibrium points and purple circles correspond to holes in the wall. (b), (e), (h), (k) Numerical solution of Equation (S4.1) at $t = 25$ and $t = 50$. The grey lines indicate the initial condition and the arrows indicate the direction of increasing time. (c), (f), (i), (l) The time evolution of the position of the leading edge of the travelling wave solution. Parameters used are (a)-(c) $P_m^i = 0.25$, $P_m^g = 0.5$, $P_p^i = P_p^g = 1$, $P_d^i = P_d^g = 0.5$, $v = 0.504$; (d)-(f) $P_m^i = 0$, $P_m^g = 1$, $P_p^i = P_p^g = 1$, $P_d^i = P_d^g = 0.1$, $v = 0.777$; (g)-(i) $P_m^i = 0.25$, $P_m^g = 0.05$, $P_p^i = P_p^g = 1$, $P_d^i = P_d^g = 0.1$, $v = 0.672$; (j)-(l) $P_m^i = 0.25$, $P_m^g = 0.05$, $P_p^i = P_p^g = 1$, $P_d^i = P_d^g = 1/6$, $v = 0.648$. All results are obtained using $\delta x = 0.1$, $\delta t = 0.01$, $\epsilon = 10^{-6}$.

introducing agent death has the effect of reducing the density sufficiently far behind the wave front, and we observe that the non-zero equilibrium point now occurs at $C = S \leq 1$.

Sub-case 4.2. **Extinction-degenerate non-negative nonlinear diffusivity function.** If $F_s(0) = 0$, $F_s(\overline{C}) > 0$ for $0 < \overline{C} \leq 1$, and $R(0) = 0$, a stretching transformation is applied to remove the singularity in Equations (S4.6)-(S4.7), giving

$$\frac{\mathrm{d}\overline{C}}{\mathrm{d}\zeta} = D_g S(4\overline{C} - 3S\overline{C}^2)\overline{U}, \tag{S4.9}$$

$$\frac{\mathrm{d}\overline{U}}{\mathrm{d}\zeta} = -v\overline{U} + D_g S(6S\overline{C} - 4)\overline{U}^2 - (\lambda - K)\overline{C}(1 - \overline{C}),\ \zeta \geq 0, \tag{S4.10}$$

as $D_i = 0$. There are now three equilibrium points: $(\overline{C}, \overline{U}) = (0, 0)$; $(\overline{C}, \overline{U}) = (1, 0)$; and $(\overline{C}, \overline{U}) = (0, -v/4SD_g)$. As for Case 2.2, the saddle-saddle connection between $(1, 0)$ and $(0, -v/4SD_g)$ only occurs for a unique wave speed, v^*, which implies that sharp-fronted solutions exist only for the minimum wave speed.

Sharp-fronted travelling wave solutions of Equation (S4.1) with extinction-degenerate non-negative $F_s(\overline{C})$ and $S = 0.9$ are shown in Figures S11(d)-(f). Introducing agent death does not change the qualitative behaviour compared to the corresponding case with $K = 0$ (Case 2.2). Specifically, $\mathrm{d}C/\mathrm{d}z$ approaches $C = 0$ with a non-zero value and hence the wave front is sharp near $C = 0$. Again, the density behind the wave front decreases such that $C = S$, corresponding to the non-zero equilibrium point.

Sub-case 4.3. **Positive-negative-positive nonlinear diffusivity function.** For positive-negative-positive $F_s(\overline{C})$, the analysis in Case 2.3 holds provided that $\lambda > K$. Specifically, the minimum wave speed condition proved by Ferracuti *et al.* [36] implies that real-valued holes in the wall will be present for the scaled Fisher-Kolmogorov equation with $\lambda > K$. In turn, this suggests that the smooth travelling wave solutions passing through the region of negative diffusivity observed for Case 2.3 will be present with non-zero K. Travelling wave behaviour for Equation (S4.1) with positive-negative-positive $F_s(\overline{C})$ is demonstrated in Figures S11(g)-(i). The travelling wave solution behaviour is similar to the behaviour in the corresponding case with $K = 0$ (Case 2.3), with the exception of the reduced carrying capacity.

Sub-case 4.4. **Capacity-degenerate positive-negative nonlinear diffusivity function.** The capacity-degenerate positive-negative diffusivity case, where $F_s(1) = R(S) = 0$, $F_s(\overline{C}) < 0$ for $\omega < \overline{C} < 1$ and $F_s(\overline{C}) \geq 0$ otherwise, might be thought to lead to travelling wave solutions with a sharp front near the carrying capacity density [38]. Similar to the approach for Case 2.4, we consider the conditions proposed by Maini *et al.* [38]. Again, we satisfy the condition that $F_s(1) = 0$. However, the minimum wave speed for the transformation of Equation (S2.1) in $0 \leq \overline{C} < \omega$ is the same as the minimum wave speed for the transformation of Equation (S2.1) in $\omega < \overline{C} \leq 1$. As such, we do not expect that Equation (S4.1) will approach a travelling wave solution with a sharp front near $\overline{C} = 1$. We present travelling wave behaviour for Equation (S4.1) with capacity-degenerate positive-negative $F_s(\overline{C})$ in Figures S11(j)-(l) and, as anticipated, observe that the travelling wave solution is a classic front.

Sub-case 4.5. **Positive-negative nonlinear diffusivity function.** The positive-negative case, where $F_s(\overline{C}) > 0$ for $0 \leq \overline{C} < \omega$ and $F_s(\overline{C}) < 0$ for $\omega < \overline{C} \leq 1$, cannot occur with $K = 0$. It is instructive to examine whether stable travelling wave solutions of Equation (S4.1) exist, as the non-zero equilibrium point now occurs in the region where $F_s(\overline{C}) < 0$. If we perform standard linear analysis on Equations (S4.3)-(S4.4), the Jacobian at $(S, 0)$ has eigenvalues $\xi = (-v \pm \sqrt{v^2 + 4F(S)(\lambda(2S - 1) + K)})/2F(S)$, which implies that the equilibrium point is an unstable node provided $v > 2\sqrt{-F(S)(\lambda(2S - 1) + K)}$. The negative sign is present as $F(S) < 0$ for positive-negative $F_s(\overline{C})$. The Jacobian at $(0, 0)$ has eigenvalues $\xi = (-v \pm \sqrt{v^2 - 4D_i(\lambda - K)})/2D_i$, which is a stable node provided that $v > 2\sqrt{(\lambda - K)D_i}$. While there are infinitely many solution trajectories out of the unstable node, we require that the solution trajectory passes through the hole in the wall,

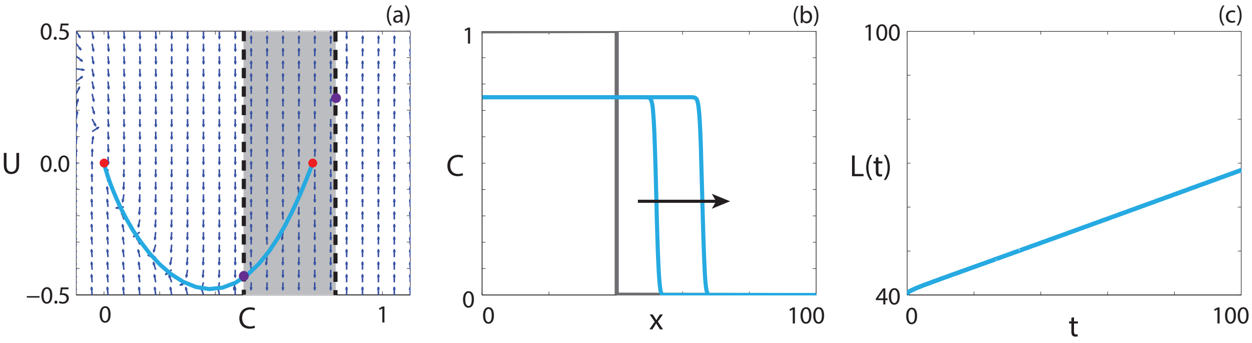

FIGURE S12. **Travelling wave behaviour for Equation** (S4.1) **with positive-negative** $F_s(\overline{C})$ **(Case 4.5)**. (a) Phase plane for the system (S4.3)-(S4.4) with the numerical solution of Equation (S4.1), in (C, U) co-ordinates, superimposed. The grey region corresponds to values of $\overline{C}$ where $F_s(\overline{C}) < 0$. The dashed black lines denote a wall of singularities. Red circles correspond to equilibrium points and purple circles correspond to holes in the wall. (b) Numerical solution of Equation (S4.1) at $t = 50$ and $t = 100$. The grey lines indicate the initial condition and the arrow indicates the direction of increasing time. (c) The time evolution of the position of the leading edge of the travelling wave solution. All results are obtained using $P_m^i = 0.05$, $P_m^g = 0.01$, $P_p^i = P_p^g = 1.0$, $P_d^i = P_d^g = 0.25$, $\delta x = 0.1$, $\delta t = 0.01$, $\epsilon = 10^{-6}$, $v = 0.2760$.

and hence there is a single solution trajectory that forms a heteroclinic orbit.

Travelling wave behaviour for Equation (S4.1) with positive-negative $F_s(\overline{C})$ is shown in Figure S12. The numerical solution of Equation (S4.1), in (C, U) co-ordinates, passes through the wall of singularities where Equation (S4.4) is finite and forms a heteroclinic orbit between $(S, 0)$ and $(0, 0)$. The travelling wave front is of classic type, a result predicted by the analysis performed by Maini *et al.* [38] as $F_s(0) \neq 0$ and $F_s(1) \neq 0$.

Case 5. EQUAL MOTILITY RATES, NO GROUPED AGENT DEATH.

Results in Figure 1 (Main Document) indicate that restricting death events to isolated agents significantly change the behaviour of the agent population. This represents a co-operative mechanism, as there is a benefit to being in close proximity to another agent. In the case where $P_d^i \neq 0$ and $P_d^g = 0$, the source term can be expressed as an Allee effect [15]

$$R(C) = rC(1 - C)(C - A), \tag{S5.1}$$

where

$$r = K_i - \lambda_i + \lambda_g, \tag{S5.2}$$

is the intrinsic growth rate, and

$$A = \frac{K_i - \lambda_i}{K_i - \lambda_i + \lambda_g}, \tag{S5.3}$$

is the Allee parameter. It follows that Equation (2) becomes

$$\frac{\partial C}{\partial t} = D\frac{\partial^2 C}{\partial x^2} + (K_i - \lambda_i + \lambda_g)C(1 - C)\left(C - \frac{K_i - \lambda_i}{K_i - \lambda_i + \lambda_g}\right). \tag{S5.4}$$

If $K_i > \lambda_i$, $R(C)$ represents the strong Allee effect, $A > 0$ [15]. The strong Allee effect has bistable growth kinetics, namely, $R(C) < 0$ for $0 < C < A$ and $R(C) > 0$ for $A < C < 1$. For low densities there are significantly more isolated agents than grouped agents, which corresponds to negative growth if $K_i > \lambda_i$. If $\lambda_i > K_i$, and $\lambda_g > 0$, $R(C) > 0$ for $0 < C < 1$. There are two possibilities for this case: $r > 0$ and $r < 0$.

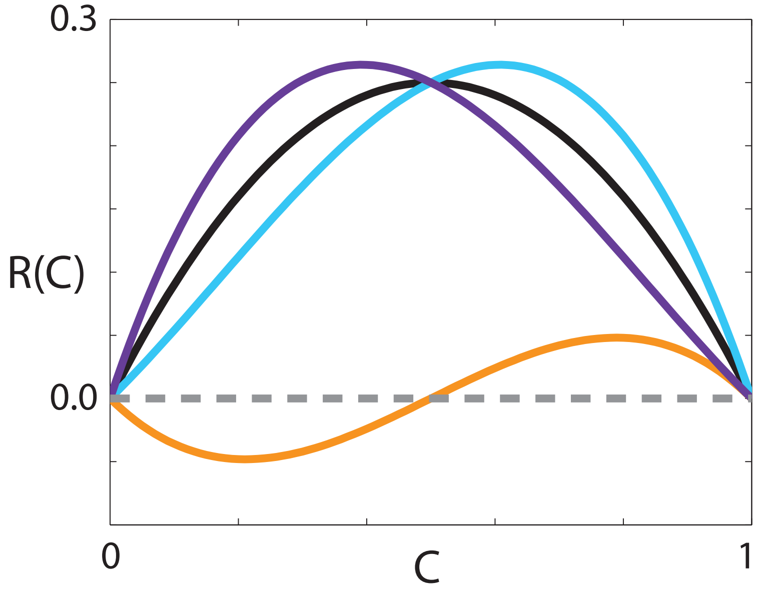

FIGURE S13. **Comparison of source terms**. $R(C)$ corresponding to the weak Allee effect with $r = 1$, $A = -0.5$ (cyan), strong Allee effect with $r = 1$, $A = 0.5$ (orange), reverse Allee effect with $r = -1$, $A = 1.5$ (purple) and logistic growth with $r = 1$ (black).

If $r > 0$ then $A < 0$ and hence the growth rate is inhibited at low density, but remains positive, which corresponds to the weak Allee effect [15]. For the case where $r < 0$ and $R(C) > 0$, we obtain $A > 1$ for all parameter combinations. Interestingly, this implies that the growth rate is inhibited at high density, but remains positive. This situation does not correspond to either the weak or strong Allee effect, and we term this behaviour the reverse Allee effect. It is not possible to have a combination of parameters that results in $r < 0$ and $0 < A < 1$ as all of our parameters are non-negative. Representative source terms showing the three types of Allee effect are compared with a logistic source term in Figure S13.

For $P_m^i = P_m^g$, we have linear diffusion in Equation (S5.4). Reaction-diffusion equations with linear diffusion and either weak or strong Allee kinetics have been well-studied [15, 19, 22, 23, 25, 26, 28, 30]. We briefly present results here and interpret these in the context of examining the long time travelling wave solution. For additional details we refer the reader to [15]. We look for solutions in the travelling wave co-ordinate $z = x - vt$. The existence of such solutions has been examined previously and requirements for the initial conditions to converge to a travelling wave solution have been found for both the case where $A < 0$ and where $0 < A < 1$ [23]. Transforming Equation (S5.4) into travelling wave co-ordinates we obtain

$$D\frac{\mathrm{d}^2C}{\mathrm{d}z^2} + v\frac{\mathrm{d}C}{\mathrm{d}z} + (K_i - \lambda_i + \lambda_g)C(1-C)\left(C - \frac{K_i - \lambda_i}{K_i - \lambda_i + \lambda_g}\right) = 0, \; -\infty < z < \infty, \tag{S5.5}$$

where $D = D_g = D_i$. If $U = \mathrm{d}C/\mathrm{d}z$, Equation (S5.5) can be expressed as

$$\frac{\mathrm{d}C}{\mathrm{d}z} = U, \tag{S5.6}$$

$$\frac{\mathrm{d}U}{\mathrm{d}z} = \frac{-vU}{D} - \frac{(K_i - \lambda_i + \lambda_g)C(1-C)(C-A)}{D}. \tag{S5.7}$$

This system of equations has three equilibrium points: $(C, U) = (0, 0)$; $(C, U) = (1, 0)$; and $(C, U) = (A, 0)$. We are only concerned with physically relevant equilibrium points, where $0 \leq C \leq 1$. If $A < 0$ or $A > 1$, there are only two physically relevant equilibrium points as the equilibrium point at $(C, U) = (A, 0)$ has no physical meaning. Performing standard linear stability analysis by examining the eigenvalues of Jacobian of the system, the characteristic equation at $(0, 0)$ has solutions $\xi = (-v \pm \sqrt{v^2 + 4(K_i - \lambda_i)D})/2D$, which implies that

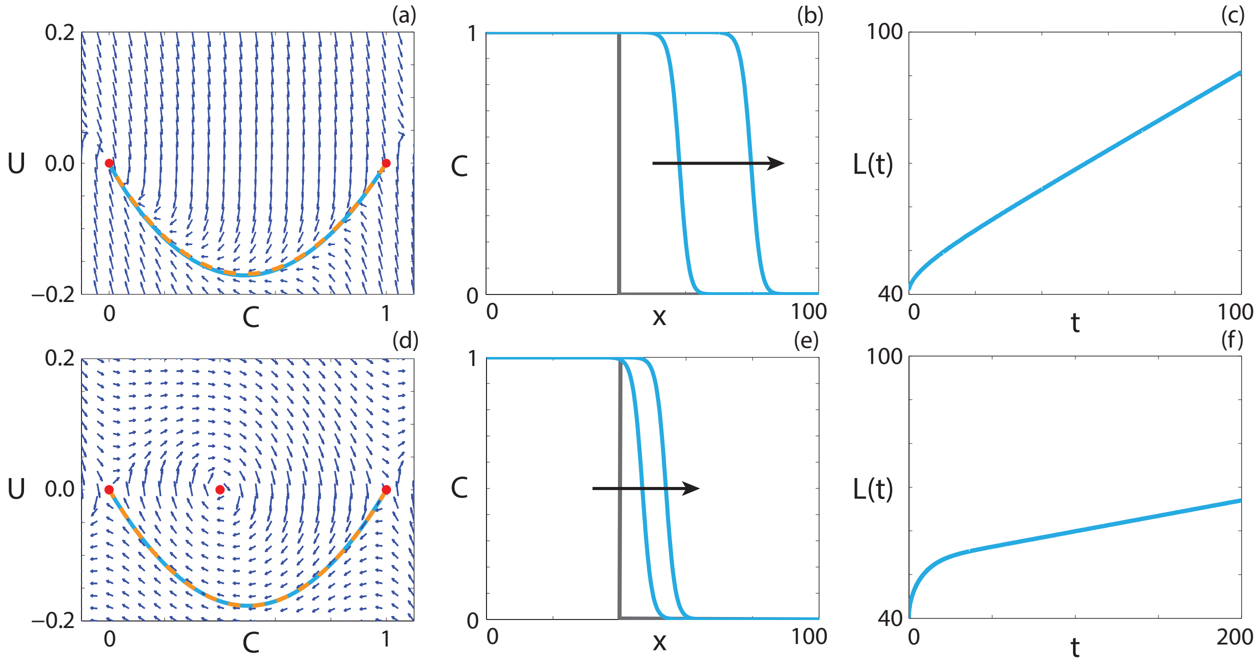

FIGURE S14. **Travelling wave behaviour for the (a)-(c) weak Allee effect and the (d)-(f) strong Allee effect with a constant $F(C)$ (Case 5).** (a), (d) Phase plane for the system (S5.6)-(S5.7) with the numerical solution of Equations (S5.4) (cyan, solid) and (S5.5) (orange, dashed), in (C, U) co-ordinates, superimposed. Red circles correspond to equilibrium points. (b), (e) Numerical solution of Equation (S5.4) calculated at (b) $t = 50$ and $t = 100$; (e) $t = 100$ and $t = 200$. The grey lines indicate the initial condition and the arrows indicate the direction of increasing time. (c), (f) The time evolution of $L(t)$. All results are obtained with $\delta x = 0.1$, $\delta t = 0.01$, $\epsilon = 10^{-6}$, $P_d^g = 0$, (a)-(c) $P_m^i = 0.5$, $P_m^g = 0.5$, $P_p^i = 0.7$, $P_p^g = 0.4$, $P_d^i = 0.5$, $v = 0.44$; (d)-(f) $P_m^i = 1.0$, $P_m^g = 1.0$, $P_p^i = 0.3$, $P_p^g = 0.3$, $P_d^i = 0.5$, $v = 0.072$.

the equilibrium point is a stable node when $\lambda_i > K_i$, provided the wave speed satisfies $v > 2\sqrt{(\lambda_i - K_i)D}$. If the wave speed does not satisfy this condition then the equilibrium point is a stable spiral, which implies that the heteroclinic orbit enters non-physical values of C. If $K_i > \lambda_i$, $(0, 0)$ is a saddle point. The characteristic equation for the equilibrium point at $(1, 0)$ has solutions $\xi = (-v \pm \sqrt{v^2 + 4\lambda_g D})/2D$, which implies that the equilibrium point is a saddle point, as λ_g is non-negative. A heteroclinic orbit between $(1, 0)$ and $(0, 0)$ exists for a unique wave speed [25]. The equilibrium point at $(A, 0)$ has a characteristic equation with solutions $\xi = (-v \pm \sqrt{v^2 - 4\lambda_g AD})/2D$. As we are only concerned with physically realistic equilibrium points, that is, where $0 < A < 1$, the equilibrium point $(A, 0)$ will be a stable node provided that the minimum wave speed $v > 2\sqrt{\lambda_g AD}$ is satisfied, and a stable spiral otherwise. The spiral behaviour does not cause the solution trajectory to become non-physical and hence this wave speed condition is not required to obtain physically meaningful solutions.

Solutions that display travelling wave behaviour for the weak Allee effect are presented in Figures S14(a)-(c). There is a heteroclinic solution trajectory for Equations (S5.6)-(S5.7) between the two equilibrium points, and the numerical solution of Equation (S5.4) matches the solution trajectory in (C, U) co-ordinates. The results in Figures S14(b)-(c) suggest that the numerical solution of Equation (S5.4) with the weak Allee effect approaches a travelling wave solution. Since the source term for the reverse Allee effect is qualitatively similar to the weak Allee effect, we do not present solutions here. Solution behaviour for the reverse Allee

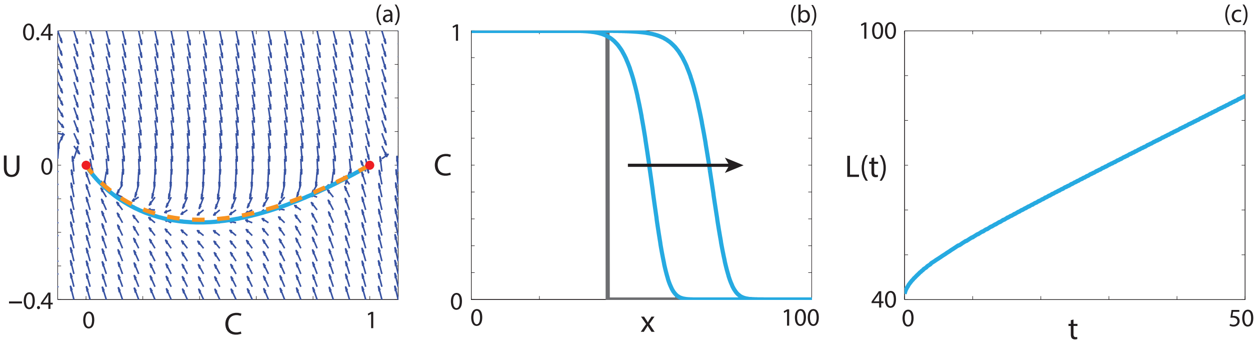

FIGURE S15. **Travelling wave behaviour for Equation** (S5.4) **with the reverse Allee effect and constant** $F(C)$ **(Case 5)**. (a) Phase plane for the system (S5.6)-(S5.7) with the numerical solution to Equations (S5.4) (cyan, solid) and (S5.5) (orange, dashed), in (C, U) co-ordinates, superimposed. Red circles correspond to equilibrium points. (b) Numerical solution to Equation (S5.4) calculated at $t = 25$ and $t = 50$. The grey lines indicate the initial condition and the arrow indicates the direction of increasing time. (c) The time evolution of the position of the leading edge of the wave front. All results are obtained with $\delta x = 0.1$, $\delta t = 0.01$, $\epsilon = 10^{-6}$, $P_m^i = 1.0$, $P_m^g = 1.0$, $P_p^i = 0.6$, $P_p^g = 0.2$, $P_d^i = 0.3$, $P_d^g = 0$, $v = 0.756$.

effect can be found in Figure S15.

A travelling wave solution of Equation (S5.4) in a parameter regime that results in a strong Allee effect is now considered. The phase plane for the system (S5.6)-(S5.7), presented in Figure S14(d), has three physically meaningful equilibrium points, and the equilibrium point at $(0, 0)$ is unstable, unlike in Figure S14(d). However, there is still a heteroclinic orbit between $(1, 0)$ and $(0, 0)$. Unlike for the weak Allee effect, the wave speed that admits this solution trajectory is unique [25]. The numerical solution of Equation (S5.4) shows the solution approaches a travelling wave, although the approach is slower than for the weak Allee effect. This is intuitive, as the growth rate for the weak Allee effect is non-negative, while the strong Allee effect has regions of negative growth.

It is instructive to consider how v depends on A. We calculate the numerical solution of Equation (S5.4) for a range of A values with $r = 1$ for $A \leq 1$, and $r = -1$ for $A > 1$, and use the numerical solution to calculate v at sufficiently late time. We consider $r < 0$ for $A > 1$ because, due to our parameters being non-negative, $A < 1$ for $r > 0$. The minimum wave speed for the travelling wave solution is known for $A \leq 0$, namely, $v^* = 2\sqrt{-ArD}$ for $A \leq -1/2$ and $v^* = 2\sqrt{rD}(1/2 - A)$ for $-1/2 \leq A \leq 0$ [28]. For $0 < A < 1$ there is a unique wave speed, $v = \sqrt{2rD}(1/2 - A)$ [28]. Consequently, for $A > 1/2$, the population will tend to extinction because the travelling wave speed is negative [28]. For the case where $A > 1$ it is unclear whether there is a minimum wave speed condition. A comparison between the observed wave speed for each A value and the predicted minimum wave speed is given in Figure S16. The predicted wave speeds match the observed wave speeds well for $A \leq 1$. For $A > 1$ we superimpose the wave speed prediction $v = 2\sqrt{-ArD}$, and observe that the predictions match the numerical wave speeds well. For the case $A > 1$ we require that $\lambda_i > K_i$, and hence the minimum wave speed condition is the same as for the weak Allee effect.

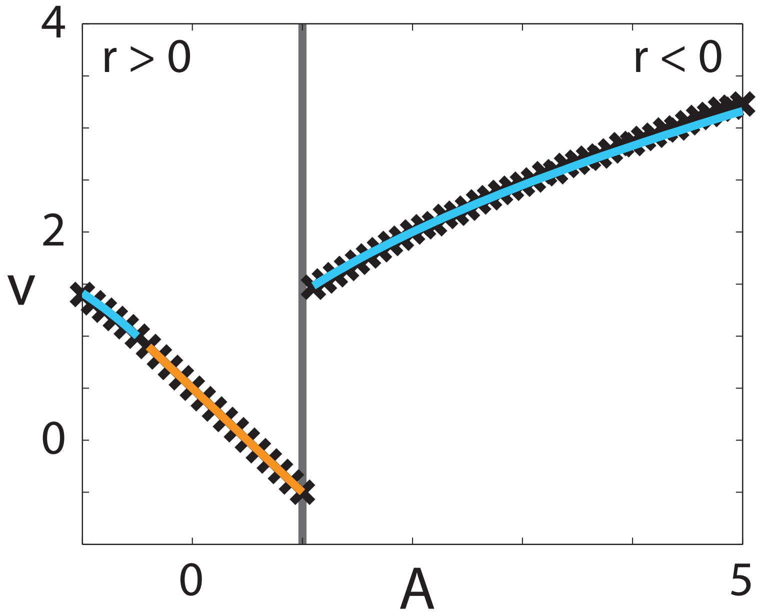

Figure S16. **Wave speed comparison for Case 5**. Comparison of observed wave speeds obtained from the numerical solution of Equation (S5.4). For $A \leq 1$ results are obtained using $r = 1$, whereas for $A > 1$ results are obtained using $r = -1$. The cyan line corresponds to $v = 2\sqrt{-ArD}$ and the orange line corresponds to $v = 2\sqrt{rD}(1/2 - A)$.

Case 6. Different motility rates, no grouped agent death.

For $P_m^i \neq P_m^g$ and $P_d^g = 0$, there is a co-operative mechanism in terms of increased survival for agents in close proximity to other agents. In this parameter regime Equation (2) becomes

$$\frac{\partial C}{\partial t} = \frac{\partial}{\partial x}\left(F(C)\frac{\partial C}{\partial x}\right) + (K_i - \lambda_i + \lambda_g)C(1-C)\left(C - \frac{K_i - \lambda_i}{K_i - \lambda_i + \lambda_g}\right), \tag{S6.1}$$

where $F(C) = D_i(1 - 4C + 3C^2) + D_g(4C - 3C^2)$. Note that $F(C)$ is the same as in Case 2 and, as such, encodes the same four types of qualitative behaviour. To examine the long term travelling wave behaviour of Equation (S6.1), we transform Equation (S6.1) into travelling wave co-ordinates, $z = x - vt$, giving

$$v\frac{\mathrm{d}C}{\mathrm{d}z} + F(C)\frac{\mathrm{d}^2C}{\mathrm{d}z^2} + (D_i - D_g)(6C-4)\left(\frac{\mathrm{d}C}{\mathrm{d}z}\right)^2 + R(C) = 0, \ -\infty < z < \infty, \tag{S6.2}$$

where $R(C) = (K_i - \lambda_i + \lambda_g)C(1-C)(C - (K_i - \lambda_i)/(K_i - \lambda_i + \lambda_g))$. Making the substitution $U = \mathrm{d}C/\mathrm{d}z$ results in

$$\frac{\mathrm{d}C}{\mathrm{d}z} = U, \tag{S6.3}$$

$$\frac{\mathrm{d}U}{\mathrm{d}z} = \frac{-vU - (D_i - D_g)(6C-4)U^2(K_i - \lambda_i + \lambda_g) - R(C))}{F(C)}. \tag{S6.4}$$

Equation (S6.4) is singular if $F(C) = 0$ for $0 \leq C \leq 1$. It is therefore of interest to determine whether travelling wave solutions can be found for each class of $F(C)$.

Sub-case 6.1. **Strictly positive nonlinear diffusivity function.** Strictly positive $F(C)$ corresponds to parameters in the grey region of Figure S2(a). As the wave speed for an arbitrary A is not determined by linear analysis for the Allee equation with constant diffusivity, we follow the approach of Hadeler [42–44] to

determine a condition for $v > 0$. Making the transformation

(S6.5)
$$\hat{z} = \int_0^z \frac{1}{D_i(1 - 4C(s) + 3C(s)^2) + D_g(4C(s) - 3C(s)^2)} \, \mathrm{d}s,$$

results in

(S6.6)
$$\frac{\mathrm{d}C}{\mathrm{d}\hat{z}} = \hat{U},$$

(S6.7)
$$\frac{\mathrm{d}\hat{U}}{\mathrm{d}\hat{z}} = -v\hat{U} - \lambda\Big(D_i(1 - 4C + 3C^2) + D_g(4C - 3C^2)\Big)(K_i - \lambda_i + \lambda_g)C(1 - C)\left(C - \frac{K_i - \lambda_i}{K_i - \lambda_i + \lambda_g}\right).$$

For $v > 0$ we require that the transformed source term is, on average, positive, which corresponds to [42–45]

(S6.8)
$$\int_0^1 (K_i - \lambda_i + \lambda_g)\Big(D_i\big(1 - 4C + 3C^2\big) + D_g\big(4C - 3C^2\big)\Big)C(1 - C)\left(C - \frac{K_i - \lambda_i}{K_i - \lambda_i + \lambda_g}\right)\mathrm{d}C > 0.$$

Condition (S6.8) is equivalent to

(S6.9)
$$(6\lambda_g - 5K_i + 5\lambda_i)D_g - \lambda_g D_i > 0.$$

For the case with $r > 0$, Condition (S6.9) is equivalent to $(A - 1)D_i + (6 - 11A)D_g > 0$. Since $D_i < 4D_g$ for $F(C) > 0$ on $0 \leq C \leq 1$, it is trivial to see that for $A < 0$, $v > 0$. Interestingly, for $A > 0$, the threshold A value for the population to persist increases if $P_m^g > P_m^i$, and decreases otherwise. For example, if $P_m^i = 0$ then $A < 6/11$ leads to persistence, higher than the threshold A value in the case with constant $F(C)$. Alternatively, as $P_m^i \to 4P_m^g$, $A \to 2/7$. This implies that populations where isolated agents are significantly more motile than grouped agents are more susceptible to extinction. This result is intuitive, as the parameter regime considered here describes a co-operative benefit, namely, a reduced death rate for agents in close proximity to other agents. Finally, for the reverse Allee case, where $r < 0$ and $A > 1$, Condition (S6.9) is always satisfied and the population persists.

Travelling wave solutions for the strong Allee effect with a strictly positive $F(C)$ are shown in Figure S17. For the strong Allee effect, with parameters that correspond to $A = 1/4$, presented in Figures S17(a)-(c), we observe a heteroclinic orbit between $(1, 0)$ and $(0, 0)$. The numerical solution of Equation (S6.1) in this parameter regime approaches a travelling wave solution with $v > 0$. However, if we consider a parameter regime that corresponds to the strong Allee effect with $A = 4/9$, presented in Figures S17(d)-(f), we observe that, while a heteroclinic orbit between $(1, 0)$ and $(0, 0)$ exists, it corresponds to a negative wave speed. As a consequence, the population tends to extinction in a birth/death parameter regime that would otherwise result in the persistence of the population if the diffusivity is constant. As both the weak and reverse Allee effect are qualitatively similar to Fisher kinetics, numerical solutions are not presented here. Numerical solutions can be found in Figure S18.

Sub-case 6.2. **Extinction-degenerate non-negative nonlinear diffusivity function.** The case where $F(0) = 0$ corresponds to parameters along the orange line in Figure S2(a). Sánchez-Garduño and Maini [41] demonstrate that Condition (S6.8) must be satisfied for travelling wave solutions to have $v > 0$. Furthermore, there is a critical wave speed that results in a sharp-fronted travelling wave [41]. From the results obtained for Case 6.1, Condition (S6.8) is always satisfied for $A < 0$ or $A > 1$. For parameter regimes where $0 < A < 1$ the choice of P_m^i and P_m^g influences whether Condition (S6.8) is satisfied. To obtain an extinction-degenerate diffusivity we require that $P_m^i = 0$. Hence (S6.9) implies that for $A < 6/11$ the wave speed will be positive. To obtain a positive wave speed with constant $F(C)$, we require $A < 1/2$, which implies that the population is more likely to persist in an parameter regime that leads to extinction-degenerate non-negative $F(C)$.

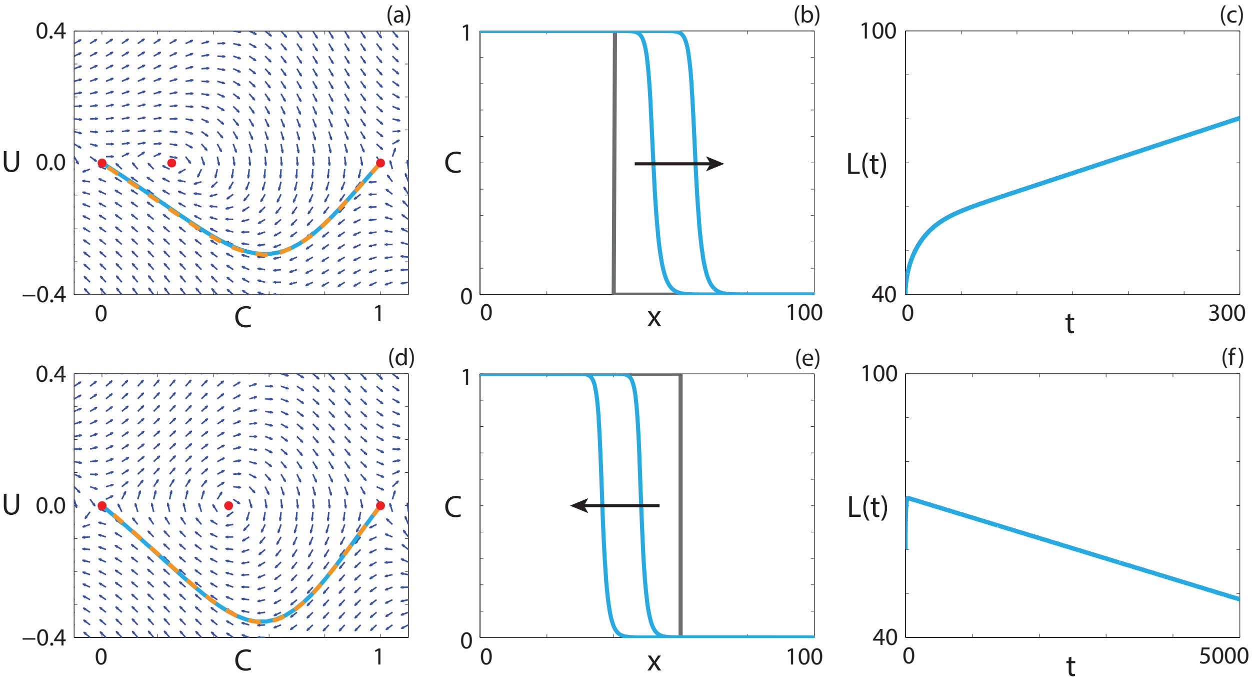

Figure S17. **Travelling wave behaviour for the Equation** (S6.1) **with the strong Allee effect and strictly positive** $F(C)$ **(Case 6.1)**. (a), (d) Phase plane for the system (S6.3)-(S6.4) with the numerical solution of Equations (S6.1) (cyan, solid) and (S6.2) (orange, dashed), in (C, U) co-ordinates, superimposed. Red circles correspond to equilibrium points. (b), (e) Numerical solution of Equation (S6.1) calculated at (b) $= 150$ and $t = 300$; (e) $t = 2500$ and $t = 5000$. The grey lines indicate the initial condition and the arrows indicate the direction of increasing time. (c), (f) The time evolution of $L(t)$. All results are obtained with $\delta x = 0.1$, $\delta t = 0.01$, $\epsilon = 10^{-6}$, $P_d^g = 0$, (a)-(c) $P_m^i = 1.0$, $P_m^g = 0.5$, $P_p^i = 0.4$, $P_p^g = 0.3$, $P_d^i = 0.5$, $v = 0.084$; (d)-(f) $P_m^i = 1.0$, $P_m^g = 0.5$, $P_p^i = 0.4$, $P_p^g = 0.3$, $P_d^i = 0.65$, $v = -0.004$.

Travelling wave behaviour for the strong Allee effect with extinction-degenerate non-negative $F(C)$ is shown in Figure S19. The numerical solution of Equation (S6.1) with $A = 1/4$, in Figures S19(a)-(c), leads to a sharp-fronted travelling wave solution near $C = 0$ with $v > 0$. With $A = 1/4$, we expect to obtain $v > 0$. For a parameter regime that results in $A = 4/7$, we obtain a travelling wave solution of Equation (S6.1) with $v < 0$ (Figures S19(d)-(f)). Interestingly, the sharp front near $C = 0$ is not present for the strong Allee effect with $v < 0$, unlike with $v > 0$, where the wave front is smooth. We present travelling wave behaviour for both the weak Allee effect and the reverse Allee effect in Figure S20.

Sub-case 6.3. **Positive-negative-positive nonlinear diffusivity function.** A positive-negative-positive $F(C)$, where there is an interval $\alpha < C < \beta$ where $F(C) < 0$, corresponds to parameter pairs highlighted in purple in Figure S2(a). Kuzmin and Ruggerini [37] examine reaction-diffusion equations with similar properties for the strong Allee effect, in the context of diffusion-aggregation models, and provide conditions for smooth travelling wave solutions to exist. For a solution with $v > 0$, we require $A < \alpha$ [37] and

$$\int_0^\alpha F(C)R(C)\ \mathrm{d}C > 0. \tag{S6.10}$$

Furthermore, we require [37]

$$3\int_0^\alpha F(C)R(C)\ \mathrm{d}C \geq \max\{\Phi(\sigma), \Phi(\rho)\}, \tag{S6.11}$$

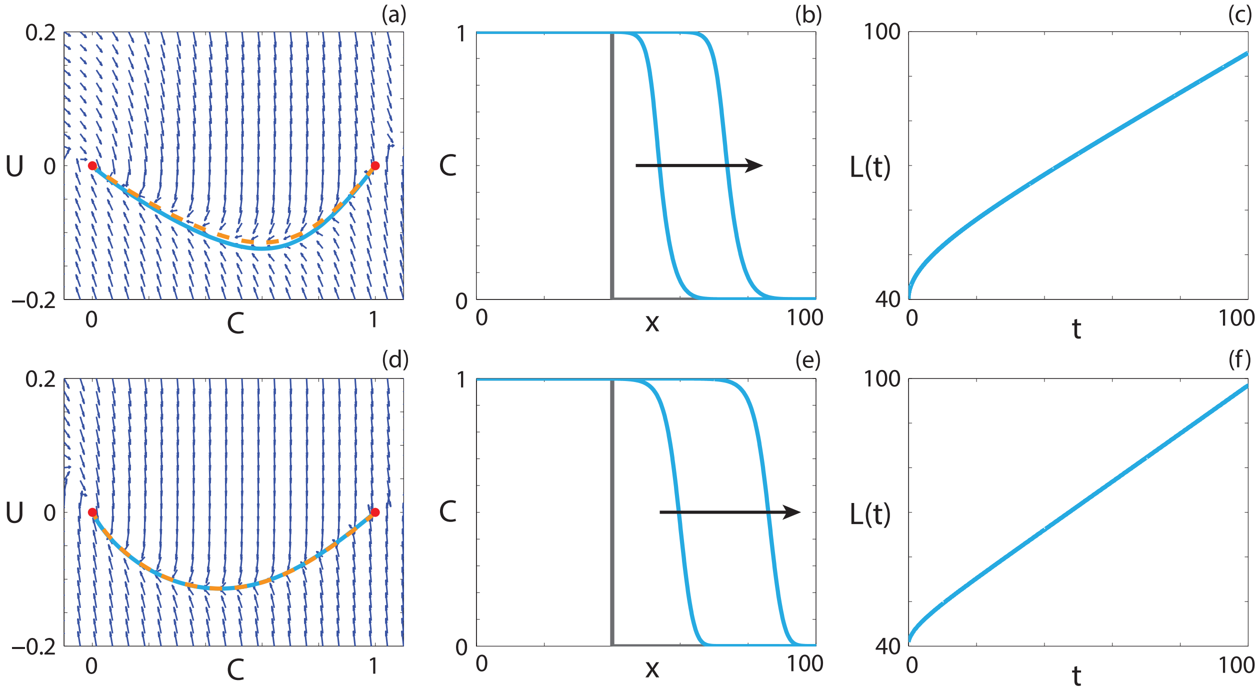

FIGURE S18. **Travelling wave behaviour for Equation** (S6.1) **with the (a)-(c) weak Allee effect and the (d)-(f) reverse Allee effect and strictly positive** $F(C)$ **(Case 6.1)**. (a),(d) Phase plane for the system (S6.3)-(S6.4) with the numerical solution to Equations (S6.1) (cyan, solid) and (S6.2) (orange, dashed), in (C, U) co-ordinates, superimposed. Red circles correspond to equilibrium points. (b),(e) Numerical solution to Equation (S6.1) calculated at $t = 50$ and $t = 100$. The grey lines indicate the initial condition and the arrows indicate the direction of increasing time. (c),(f) The time evolution of the position of the leading edge of the wave front. All results are obtained with $\delta x = 0.1$, $\delta t = 0.01$, $\epsilon = 10^{-6}$, $P_d^g = 0$, (a)-(c) $P_m^i = 1.0$, $P_m^g = 0.5$, $P_p^i = 0.4$, $P_p^g = 0.3$, $P_d^i = 0.3$, $v = 0.448$; (d)-(f) $P_m^i = 0.5$, $P_m^g = 0.25$, $P_p^i = 0.6$, $P_p^g = 0.2$, $P_d^i = 0.3$, $v = 0.536$.

where

$$\Phi(y) = 8\alpha^2 y + 4\sqrt{4\alpha^2 y^2 - 2m\alpha^3 y},$$

$$\sigma = \sup_{C\in[\alpha,\beta)} \left[\frac{F(C)R(C)}{C-\beta}\right], \quad \rho = \sup_{C\in(\beta,1]} \left[\frac{F(C)R(C)}{C-\beta}\right], \text{ and}$$

$$m = \min_{C\in[0,A]} \left[F(C)R(C)\right].$$

A suite of P_m^g values with $P_m^i = 1$, which correspond to $1/3 < \alpha < 2/3$, are considered for parameter regimes that result in $A < \alpha$. Figures S21(a)-(c) show the parameter spaces, (A, α), that satisfy Condition (S6.10), Condition (S6.11) and Conditions (S6.10)-(S6.11) simultaneously, respectively. Orange regions represent parameter pairs where the condition is satisfied and grey regions represent parameter pairs where the condition is not satisfied. These results suggest that smooth travelling wave solutions should exist for certain choices of parameters. Interestingly, all parameter pairs that satisfy Condition (S6.10) also satisfy Condition (S6.11).

For Case 2.3 and Case 4.3, smooth travelling wave solutions that pass through holes in the wall of singularities for positive-negative-positive $F(C)$ are obtained. The minimum wave speed bound presented by Ferracuti *et al.* [36] implies that the location of the holes in the wall occur are real-valued for the wave speed arising from the Heaviside initial condition. As such, to obtain smooth travelling wave solutions of Equation (S6.1)

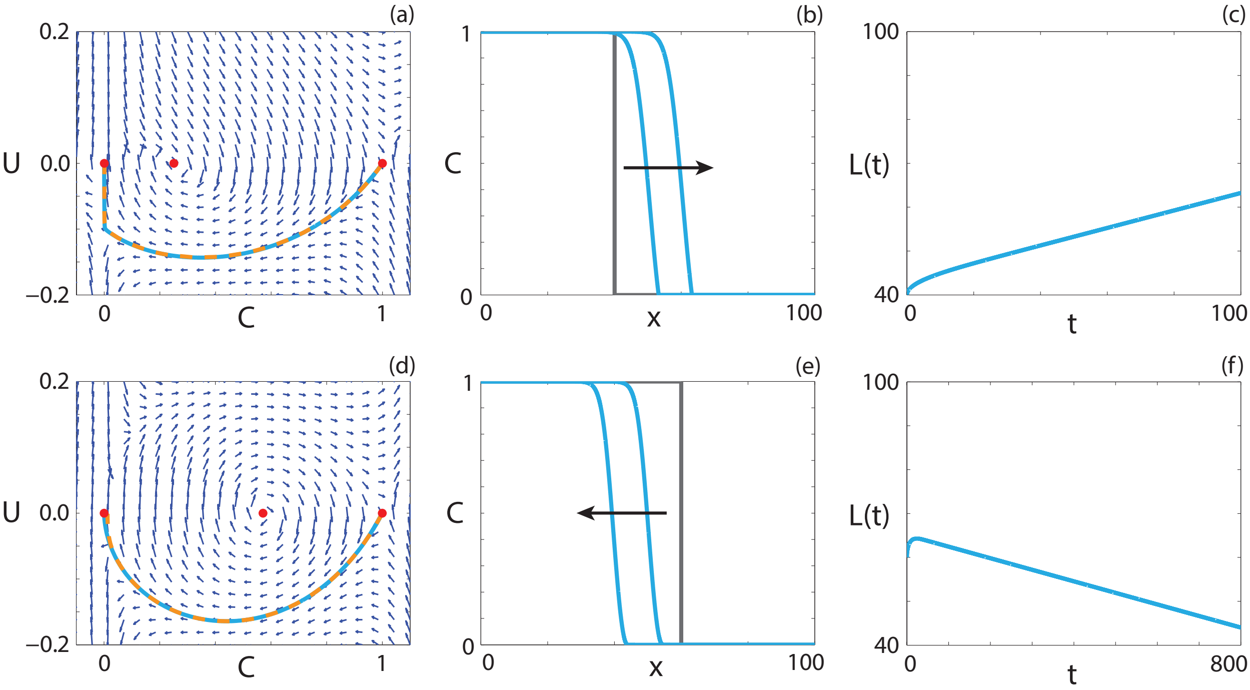

FIGURE S19. **Travelling wave behaviour for Equation** (S6.1) **with the strong Allee effect and extinction-degenerate non-negative** $F(C)$ **(Case 6.2)**. (a), (d) Phase plane for the system (S6.3)-(S6.4) with the numerical solution of Equations (S6.1) (cyan, solid) and (S6.2) (orange, dashed), in (C, U) co-ordinates, superimposed. Red circles correspond to equilibrium points. (b), (e) Numerical solution of Equation (S6.1) calculated at (b) $t = 50$ and $t = 100$; (e) $t = 400$ and $t = 800$. The grey lines indicate the initial condition and the arrows indicate the direction of increasing time. (c), (f) The time evolution of $L(t)$. All results are obtained with $\delta x = 0.01$, $\delta t = 0.005$, $\epsilon = 10^{-6}$, $P_m^i = 0$, $P_m^g = 1.0$,$P_d^g = 0$, (a)-(c) $P_p^i = 0.4$, $P_p^g = 0.3$, $P_d^i = 0.5$, $v = 0.199$; (d)-(f) $P_p^i = 0.4$, $P_p^g = 0.3$, $P_d^i = 0.8$, $v = -0.026$.

with positive-negative-positive $F(C)$, we might expect that the wave speed satisfies $v > 2\sqrt{F'(\beta)R(\beta)}$, such that the holes in the wall at $C = \beta$ are real-valued.

Following the approach used for Case 2.3, it is simple to demonstrate that both the weak and reverse Allee effect have real-valued holes in the wall. As such, we observe heteroclinic orbits between $(1, 0)$ and $(0, 0)$ that pass through the holes in the wall, and present the corresponding travelling wave solutions in Figure S22. We now examine numerical solutions of Equation (S6.1) with the strong Allee effect. For parameter regimes that give rise to wave speeds that satisfy $v > 2\sqrt{F'(\beta)R(\beta)}$, numerical travelling wave solutions could not be found. While the condition for real-valued holes in the wall is satisfied, the zeros of Equation (S6.4) are imaginary for a certain interval of $C > \beta$. This corresponds to a nullcline that is not real-valued for certain C values.

We now consider parameter regimes corresponding to the strong Allee effect with the additional restriction that $v < 2\sqrt{F'(C)R(C)}$ for $2/3 < C \leq 1$. For all P_m^i and P_m^g that give rise to a positive-negative-positive $F(C)$, holes in the wall at $C = \beta$ do not exist and, as such, we do not expect to obtain smooth solutions. Interestingly, we observe travelling wave solutions with shocks such that the solution never enters the region $\alpha < C < \beta$. An example of a shock-fronted travelling wave solution for the strong Allee effect with both

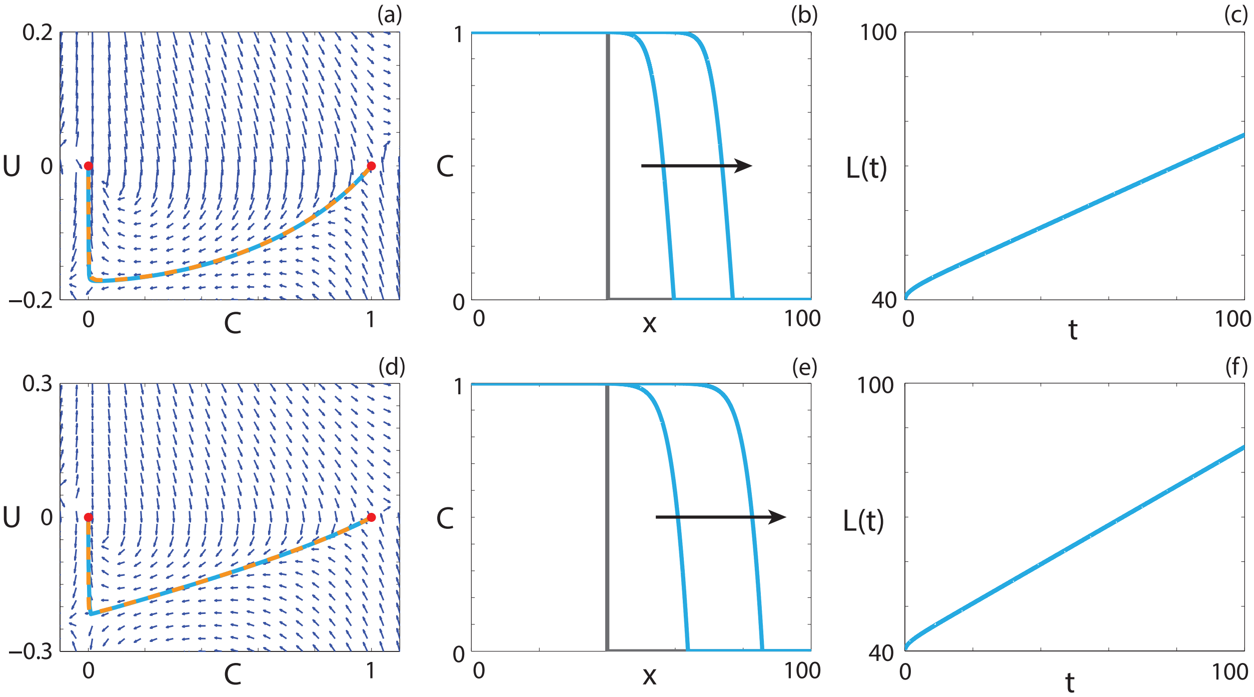

FIGURE S20. **Travelling wave behaviour for Equation** (S6.1) **with the (a)-(c) weak Allee effect and the (d)-(f) reverse Allee effect and extinction-degenerate non-negative $F(C)$ (Case 6.2).** (a),(d) Phase plane for the system (S6.3)-(S6.4) with the numerical solution to Equations (S6.1) (cyan, solid) and (S6.2) (orange, dashed), in (C, U) co-ordinates, superimposed. Red circles correspond to equilibrium points. (b),(e) Numerical solution to Equation (S6.1) calculated at $t = 50$ and $t = 100$. The grey lines indicate the initial condition and the arrows indicate the direction of increasing time. (c),(f) The time evolution of the position of the leading edge of the wave front. All results are obtained with $\delta x = 0.1$, $\delta t = 0.01$, $\epsilon = 10^{-6}$, $P_m^i = 0$, $P_m^g = 1.0$, $P_d^g = 0$, (a)-(c) $P_p^i = 0.4$, $P_p^g = 0.3$, $P_d^i = 0.3$, $v = 0.347$; (d)-(f) $P_p^i = 0.6$, $P_p^g = 0.2$, $P_d^i = 0.3$, $v = 0.438$.

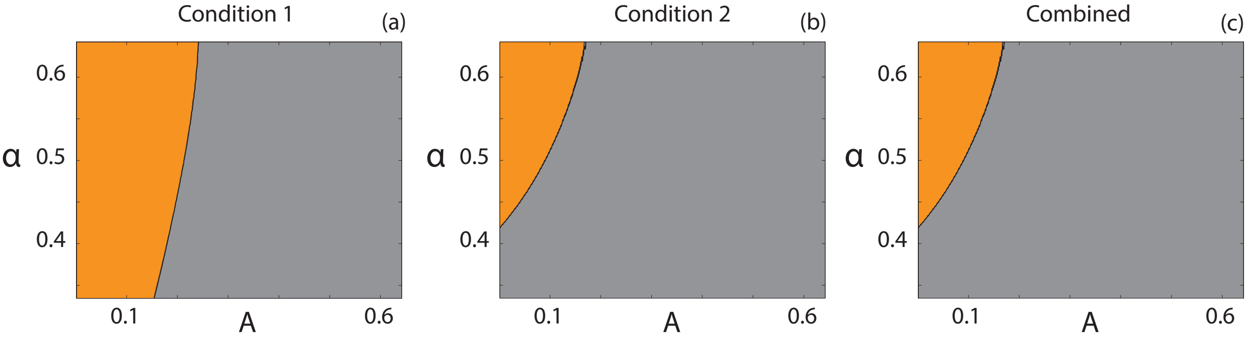

FIGURE S21. **Parameter pairs that satisfy Kuzmin and Ruggerini's Conditions [37].** (a) Condition (S6.10); (b) Condition (S6.11); (c) Conditions (S6.10)-(S6.11) combined. Orange regions correspond to parameter pairs that satisfy the respective condition(s), whereas grey regions correspond to parameter pairs that do not.

$v > 0$ and $v < 0$ is shown in Figures S23(a)-(c) and Figures S23(d)-(f), respectively. Solutions of diffusion equations, without any source terms, that contain shocks have been reported previously [50, 51]. Similarly,

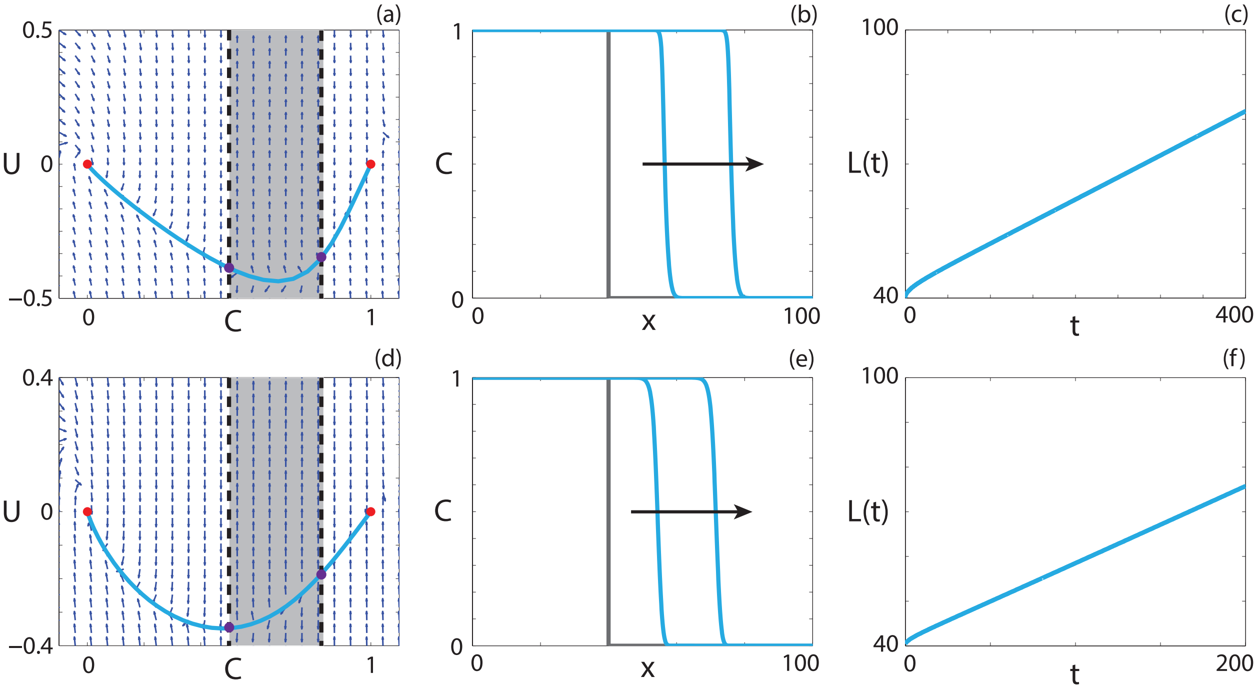

Figure S22. **Travelling wave behaviour for Equation** (S6.1) **with the (a)-(c) weak Allee effect and the (d)-(f) reverse Allee effect and positive-negative-positive** $F(C)$ **(Case 6.3)**. (a),(d) Phase plane for the system (S6.3)-(S6.4) with the numerical solution to Equation (S6.1) (cyan, solid), in (C, U) co-ordinates, superimposed. The dashed black lines denote a wall of singularities. Red circles correspond to equilibrium points and purple circles correspond to holes in the wall. (b),(e) Numerical solution to Equation (S6.1) calculated at (b) $t = 200$ and $t = 400$; (e) $t = 100$ and $t = 200$. The grey lines indicate the initial condition and the arrows indicate the direction of increasing time. (c),(f) The time evolution of the position of the leading edge of the wave front. All results are obtained with $\delta x = 0.1$, $\delta t = 0.01$, $\epsilon = 10^{-6}$, $P_d^g = 0$, (a)-(c) $P_m^i = 0.05$, $P_m^g = 0.01$, $P_p^i = 0.4$, $P_p^g = 0.3$, $P_d^i = 0.3$, $v = 0.098$; (d)-(f) $P_m^i = 0.5$, $P_m^g = 0.1$, $P_p^i = 0.6$, $P_p^g = 0.2$, $P_d^i = 0.3$, $v = 0.172$.

shock-fronted travelling wave solutions arise in other kinds of models, including multispecies models of combustion [54] and haptotactic cell migration [53]. However, the models presented here are very different, and it is therefore of interest to determine the properties of the reaction-diffusion equation that lead to shock-fronted travelling wave solutions.

Sub-case 6.4. **Capacity-degenerate positive-negative nonlinear diffusivity function.** Capacity-degenerate positive-negative $F(C)$, where $F(1) = 0$, arises if $P_m^g = 0$ and includes an interval $1/3 < C < 1$ where $F(C) < 0$. For Case 2.4, despite the degenerate nature of the nonlinear diffusivity function at $C = 1$, we did not obtain solutions with a sharp front near $C = 1$. Instead, the solution passes through the region of negative diffusivity and a hole in the wall at $C = 1/3$, leading to smooth travelling wave solutions. As such, we expect similar solutions for both the weak and reverse Allee effect due to the qualitatively similar behaviour of the $R(C)$ function. It is of interest to examine whether smooth or shock-fronted travelling wave solutions arise from Equation (S6.1) for the strong Allee effect, as for the positive-negative-positive diffusivity examined for Case 6.3 no smooth travelling wave solutions could be found.

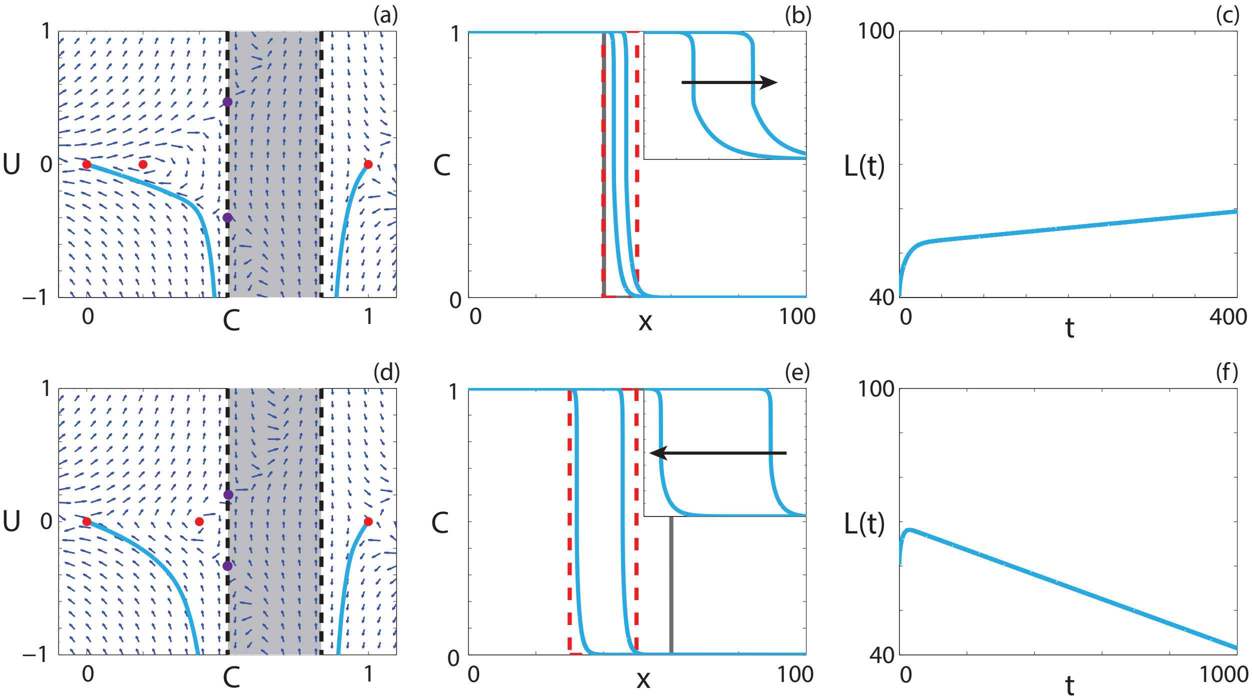

FIGURE S23. **Travelling wave behaviour for Equation** (S6.1) **with the strong Allee effect and positive-negative-positive** $F(C)$ **(Case 6.3)**. (a), (d) Phase plane for the system (S6.3)-(S6.4) with the numerical solution of Equation (S6.1) (cyan, solid), in (C, U) co-ordinates, superimposed. The dashed black lines denote a wall of singularities. Red circles correspond to equilibrium points and purple circles correspond to holes in the wall. (b), (e) Numerical solution of Equation (S6.1) calculated at (b) $t = 200$ and $t = 400$; (e) $t = 500$ and $t = 1000$. The grey lines indicate the initial condition and the arrows indicate the direction of increasing time. The insets correspond to the areas within the red dashed lines, and highlight the shocks. (c), (f) The time evolution of $L(t)$. All results are obtained with $\delta x = 0.05$, $\delta t = 0.001$, $\epsilon = 10^{-6}$, $P_d^g = 0$, (a)-(c) $P_m^i = 0.5$, $P_m^g = 0.1$, $P_p^i = 0.5$, $P_p^g = 0.4$, $P_d^i = 0.6$, $v = 0.009$; (d)-(f) $P_m^i = 0.5$, $P_m^g = 0.1$, $P_p^i = 0.4$, $P_p^g = 0.2$, $P_d^i = 0.5$, $v = -0.028$.

As expected, smooth travelling wave solutions for both the weak and reverse Allee effects with capacity-degenerate positive-negative $F(C)$ are obtained. The solution behaviour for both the weak and reverse Allee effects are presented in Figure S24. For the strong Allee effect, we examine a considerable number of parameter regimes and initial conditions and are unable to find travelling wave solutions.

Case 7. EQUAL MOTILITY RATES, DIFFERENT DEATH RATES.

Without the restriction that only isolated agents are able to undergo death events ($P_d^g \neq 0$), death events can be considered as either a co-operative mechanism ($P_d^i > P_d^g$), such as group defence against predation, or a competitive mechanism ($P_d^i < P_d^g$), where a population is more easily discovered and eradicated, compared to an isolated individual. In these parameter regimes, Equation (2) can be expressed as

$$\frac{\partial C}{\partial t} = D\frac{\partial^2 C}{\partial x^2} + (K_i - K_g - \lambda_i + \lambda_g)A_1 C\left(1 - \frac{C}{A_1}\right)\left(C - A_2\right), \tag{S7.1}$$

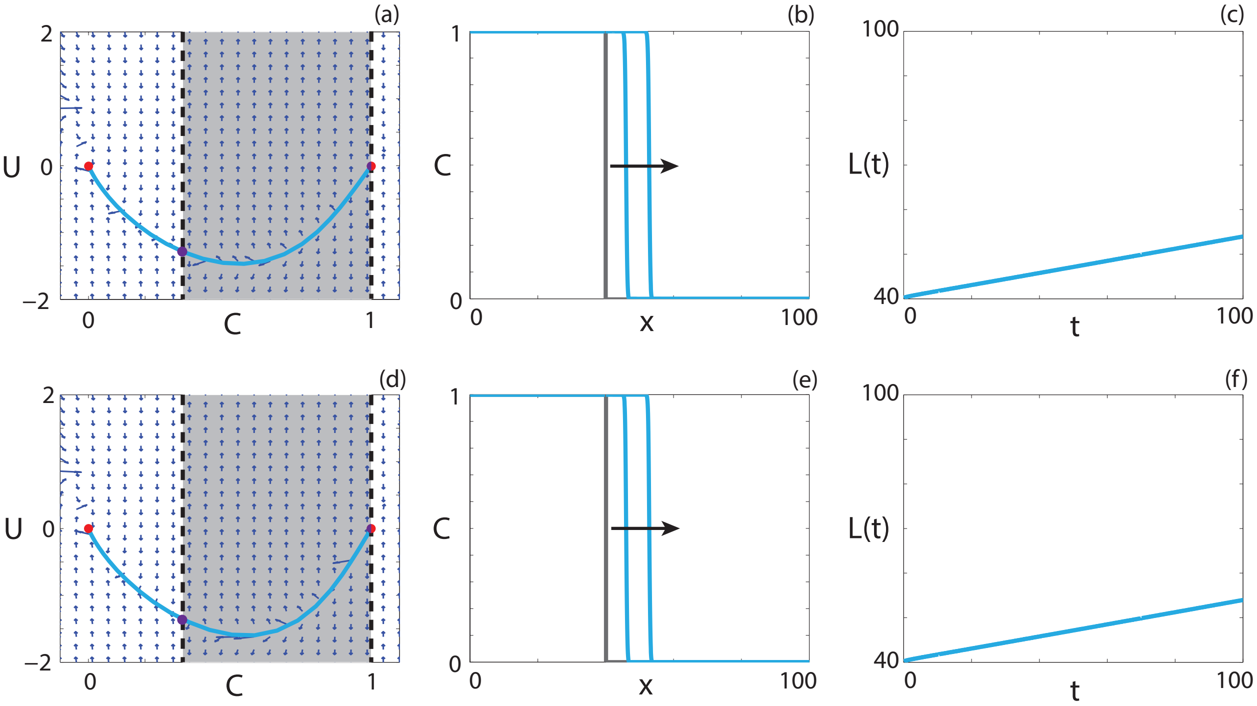

FIGURE S24. **Travelling wave behaviour for the (a)-(c) weak Allee effect and the (d)-(f) reverse Allee effect with capacity-degenerate $F(C)$ (Case 6.4).** (a), (d) Phase plane for the system (S6.3)-(S6.4) with the numerical solution of Equation (S6.1) (cyan, solid), in (C, U) co-ordinates, superimposed. The dashed black lines denote a wall of singularities. Red circles correspond to equilibrium points and purple circles correspond to holes in the wall. (b), (e) Numerical solution of Equation (S6.1) calculated at $t = 50$ and $t = 100$. The grey lines indicate the initial condition and the arrows indicate the direction of increasing time. (c), (f) The time evolution of $L(t)$. All results are obtained with $\delta x = 0.1$, $\delta t = 0.01$, $\epsilon = 10^{-6}$, $P_m^i = 0.01$, $P_m^g = 0$, $P_p^i = 1.0$, $P_d^i = 0.1$, $P_d^g = 0$, (a)-(c) $P_p^g = 0.8$, $v = 0.098$; (d)-(f) $P_p^g = 0.95$, $v = 0.136$.

where

$$A_1 = \frac{2\lambda_i - \lambda - 2K_i + 2K_g - \sqrt{\lambda_g^2 + 4K_g(\lambda_i - \lambda_g - K_i + K_g)}}{2(\lambda_i - \lambda_g - K_i + K_g)},$$

(S7.2) $$A_2 = \frac{2\lambda_i - \lambda - 2K_i + 2K_g + \sqrt{\lambda_g^2 + 4K_g(\lambda_i - \lambda_g - K_i + K_g)}}{2(\lambda_i - \lambda_g - K_i + K_g)},$$

provided that $\lambda_g \geq 2(K_g + \sqrt{Kg(K_i - \lambda_i)})$ or $\lambda_i > K_i$. If this is not satisfied, $R(C) \leq 0$ for $0 \leq C \leq 1$ and the population will tend to extinction. The corresponding ODE in travelling wave co-ordinates is

(S7.3) $$v\frac{\mathrm{d}C}{\mathrm{d}z} + D\frac{\mathrm{d}^2C}{\mathrm{d}z^2} + (K_i - K_g - \lambda_i + \lambda_g)A_1C\left(1 - \frac{C}{A_1}\right)\left(C - A_2\right) = 0, \quad -\infty < z < \infty,$$

and, making the substitution $U = \mathrm{d}C/\mathrm{d}z$, results in

(S7.4) $$\frac{\mathrm{d}C}{\mathrm{d}z} = U,$$

(S7.5) $$\frac{\mathrm{d}U}{\mathrm{d}z} = -\frac{vU}{D} - \frac{(K_i - K_g - \lambda_i + \lambda_g)A_1C}{D}\left(1 - \frac{C}{A_1}\right)\left(C - A_2\right).$$

Introducing a new variable $\overline{C} = C/A_1$ which, upon substitution into Equation (S7.1), results in

$$\frac{\partial \overline{C}}{\partial t} = D\frac{\partial^2 \overline{C}}{\partial x^2} + (K_i - K_g - \lambda_i + \lambda_g)A_1^2\overline{C}(1-\overline{C})\left(\overline{C} - \overline{A}\right), \tag{S7.6}$$

where

$$\overline{A} = \frac{A_2}{A_1} = \frac{2\lambda_i - \lambda - 2K_i + 2K_g + \sqrt{\lambda_g^2 + 4K_g(\lambda_i - \lambda_g - K_i + K_g)}}{2\lambda_i - \lambda - 2K_i + 2K_g - \sqrt{\lambda_g^2 + 4K_g(\lambda_i - \lambda_g - K_i + K_g)}}. \tag{S7.7}$$

Equation (S7.6) is a reaction-diffusion equation with Allee kinetics in terms of the scaled variable $\overline{C}$. Both the carrying capacity and Allee parameter are scaled by A_1, which influences the maximum population density as well as the threshold density required for positive growth. Following the analysis for Case 5, the minimum wave speed for Equation (S7.6) with $\overline{A} < -1/2$ is $v^* = 2\sqrt{(\lambda_i - K_i)D}$. Interestingly, this implies that introducing grouped agent death at a rate that does not result in a population tending to extinction has no influence on the invasion speed of the population. Specifically, the condition for $A < -1/2$ in Case 5 corresponds to $3(\lambda_i - K_i) > \lambda_g$. It can be shown that, with $3(\lambda_i - K_i) > \lambda_g$, we require $3K_g < \lambda_g$ for $\overline{A} < -1/2$. This implies that there is a range of K_g values that result in a travelling wave with a minimum wave speed that is independent of both K_g and λ_g. Interestingly, this suggests that if a control is implemented that increases the death rate of grouped agents, there is a threshold value for the control to influence the invasion speed and the subsequent persistence of the population. Introducing a non-zero K_g value for a parameter regime that results in the strong Allee effect with $K_g = 0$ never changes the type of Allee effect. Hence it is possible to go from a weak Allee effect to a reverse Allee effect by introducing a non-zero K_g value. Non-zero K_g values correspond to a decreased benefit for grouped agents, which explains why the source term, previously a weak Allee effect, becomes the reverse Allee effect, corresponding to inhibited growth at high density.

For the strong Allee effect, corresponding to $0 < A_2 < A_1 \leq 1$, the unique wave speed is $v = 2\sqrt{(K_i - K_g - \lambda_i + \lambda_g)D}(A_1/A_2)$ [28]. This implies that for $A_2 > A_1/2$, $v < 0$ and $v > 0$ otherwise. Furthermore, the same wave speed applies for $-A_1/2 < A_2 < 0$ [28]. For both intervals, the minimum wave speed does depend on the K_g value, and hence implementing any kind of partial eradication of the grouped agents will either reduce the speed of invasion or cause the extinction of the population.

Travelling wave behaviour for the weak and strong Allee effect and constant $F(C)$ is shown in Figure S25. For both numerical solutions, calculated with $K_g = 0.1$, the carrying capacity is reduced by approximately 27%. With the exception of K_g, the parameters used to obtain the numerical solutions in Figures S25(a)-(c) are the same as in Figures S14(a)-(c) and we observe that, as expected, the wave speed is the same. This demonstrates that, while the carrying capacity is reduced, the population is able to invade vacant space at the same speed, even though a control measure for the grouped agents has been implemented. Results for the reverse Allee effect are presented in Figure S24.

Case 8. Different motility rates, different death rates.

Setting $P_m^i \neq P_m^g$ and $P_d^i \neq P_d^g \neq 0$ allows for significant flexibility in describing a combination of competitive and/or co-operative mechanisms, depending on the relevant motivation. In this case, Equation (2) can be expressed as

$$\frac{\partial C}{\partial t} = \frac{\partial}{\partial x}\left(F(C)\frac{\partial C}{\partial x}\right) + (K_i - K_g - \lambda_i + \lambda_g)A_1C\left(1 - \frac{C}{A_1}\right)\left(C - A_2\right), \tag{S8.1}$$

where $F(C) = D_i(1 - 4C + 3C^2) + D_g(4C - 3C^2)$. Note that, again, this simplification requires that $\lambda_g \geq 2(K_g + \sqrt{Kg(K_i - \lambda_i)})$ or $\lambda_i > K_i$, otherwise the population will tend to extinction. In travelling

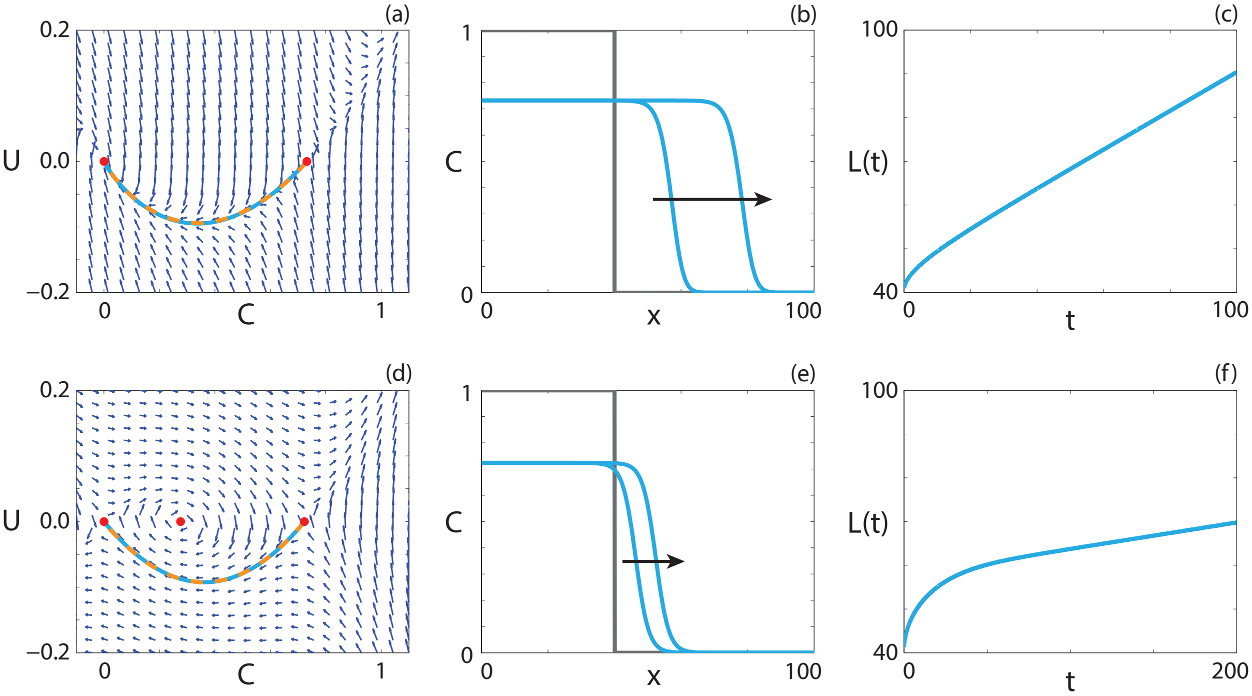

FIGURE S25. **Travelling wave behaviour for the (a)-(c) weak Allee effect and the (d)-(f) strong Allee effect with constant $F(C)$ (Case 7).** (a), (d) Phase plane for the system (S7.4)-(S7.5) with the numerical solution of Equations (S7.1) (cyan, solid) and (S7.3) (orange, dashed), in (C, U) co-ordinates, superimposed. Red circles correspond to equilibrium points. (b), (e) Numerical solution of Equation (S7.1) calculated at (b) $t = 50$ and $t = 100$; (e) $t = 100$ and $t = 200$. The grey lines indicate the initial condition and the arrows indicate the direction of increasing time. (c), (f) The time evolution of $L(t)$. All results are obtained with $\delta x = 0.1$, $\delta t = 0.01$, $\epsilon = 10^{-6}$, $P_d^g = 0.1$, (a)-(c) $P_m^i = 0.5$, $P_m^g = 0.5$, $P_p^i = 0.7$, $P_p^g = 0.4$, $P_d^i = 0.5$, $v = 0.44$; (d)-(f) $P_m^i = 1.0$, $P_m^g = 1.0$, $P_p^i = 0.7$, $P_p^g = 0.5$, $P_d^i = 0.8$, $v = 0.06$.

wave co-ordinates, Equation (S8.1) is

$$\text{(S8.2)}\quad v\frac{\mathrm{d}C}{\mathrm{d}z}+F(C)\frac{\mathrm{d}^2C}{\mathrm{d}z^2}+F'(C)\left(\frac{\mathrm{d}C}{\mathrm{d}z}\right)^2+(K_i-K_g-\lambda_i+\lambda_g)A_1C\left(1-\frac{C}{A_1}\right)\left(C-A_2\right)=0,\quad -\infty<z<\infty,$$

and, making the substitution $U = \mathrm{d}C/\mathrm{d}z$, it corresponds to

$$\text{(S8.3)}\qquad \frac{\mathrm{d}C}{\mathrm{d}z}=U,$$

$$\text{(S8.4)}\qquad \frac{\mathrm{d}U}{\mathrm{d}z}=-\frac{vU}{F(C)}-\frac{(D_i-D_g)(6C-4)U^2}{F(C)}-\frac{(K_i-K_g-\lambda_i+\lambda_g)A_1C}{F(C)}\left(1-\frac{C}{A_1}\right)\left(C-A_2\right).$$

Introducing the variable $\overline{C} = C/A_1$, Equation (S8.1) can be written as

$$\text{(S8.5)}\qquad \frac{\partial\overline{C}}{\partial t}=\frac{\partial}{\partial x}\left(F_A(\overline{C})\frac{\partial\overline{C}}{\partial x}\right)+(K_i-K_g-\lambda_i+\lambda_g)A_1^2\overline{C}(1-\overline{C})(\overline{C}-\overline{A}),$$

where $F_A(\overline{C}) = F(A_1\overline{C}) = D_i(1-4A_1\overline{C}^2+3A_1^2\overline{C}^2)+D_g(4A_1\overline{C}-3A_1^2\overline{C}^2)$. The transformed nonlinear diffusivity, $F_A(\overline{C})$, has the same characteristics as $F_s(\overline{C})$, presented in Figure S10, albeit in terms of the scaled Allee carrying capacity, A_1. Here we examine the five types of $F_A(\overline{C})$ for $A_1 \neq 1$.

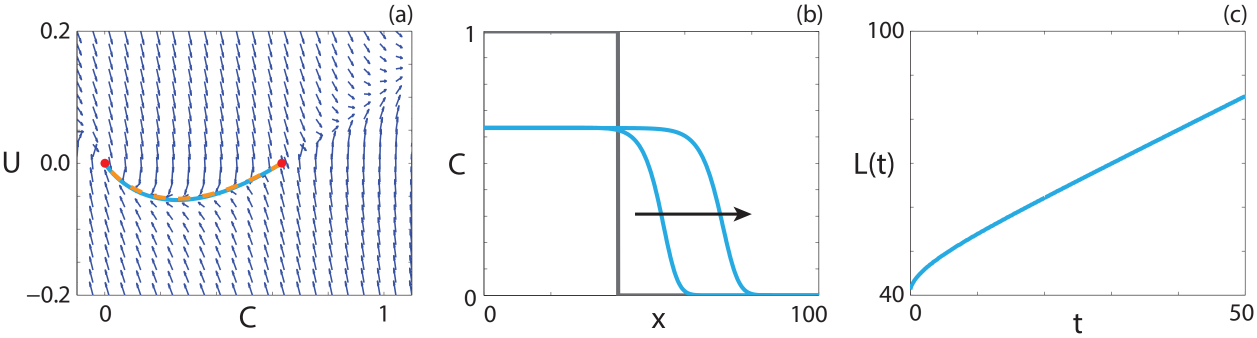

FIGURE S26. **Travelling wave behaviour for Equation** (S7.1) **with the reverse Allee effect and constant** $F_s(\overline{C})$ **(Case 7)**. (a) Phase plane for the system (S7.4)-(S7.5) with the numerical solution to Equations (S7.1) (cyan, solid) and (S7.3) (orange, dashed), in (C, U) co-ordinates, superimposed. Red circles correspond to equilibrium points. (b) Numerical solution to Equation (S7.1) calculated at $t = 25$ and $t = 50$. The grey lines indicate the initial condition and the arrow indicates the direction of increasing time. (c) The time evolution of the position of the leading edge of the wave front. All results are obtained with $\delta x = 0.1$, $\delta t = 0.01$, $\epsilon = 10^{-6}$, $P_m^i = 1.0$, $P_m^g = 1.0$, $P_p^i = 0.6$, $P_p^g = 0.2$, $P_d^i = 0.3$, $P_d^g = 0.1$, $v = 0.760$.

Sub-case 8.1. **Strictly positive nonlinear diffusivity function.** For $F_A(\overline{C}) > 0$ on the interval $0 < \overline{C} \leq 1$, we follow the approach of Hadeler [42–44]. The integral condition for the wave speed to be positive,

$$\text{(S8.6)} \quad \int_0^1 (K_i - K_g - \lambda_i + \lambda_g) A_1^2 (D_i(1 - 4A_1\overline{C}^2 + 3A_1^2\overline{C}^2) + D_g(4A_1\overline{C} - 3A_1^2\overline{C}^2))\overline{C}(1-\overline{C})(\overline{C} - \overline{A}) \, \mathrm{d}\overline{C} > 0,$$

corresponds to

$$\text{(S8.7)} \qquad D_i(5 - 10\overline{A} + 6A_1^2 - 9A_1A_2 - 12A_1 + 20A_2) - D_g(6A_1^2 - 9A_1A_2 - 12A_1 + 20A_2) > 0.$$

If $D_i = D_g$, then $\overline{A} > 1/2$ leads to $v < 0$. For the strong Allee effect, $A_1 > A_2 = \overline{A}A_1$, we can determine the threshold value for the persistence of the population, namely,

$$\text{(S8.8)} \qquad \overline{A} < \frac{5D_i + (D_i - D_g)(6A_1^2 - 12A_1)}{10D_i + (D_i - D_g)(9A_1^2 - 20A_1)}.$$

Considering the two limiting cases, where $D_i = 0$ and $D_i = 4D_g$, $\overline{A}$ takes on a value of $(6A_1^2 - 12A_1)/(9A_1^2 - 20A_1)$ and $(18A_1^2 - 36A_1 + 20)/(27A_1^2 - 60A_1 + 30)$, respectively. These values reduce to 6/11 and 2/7 in the case that $A_1 = 1$, as in Case 6.1. To illustrate how the threshold value changes with A_1, P_m^i and P_m^g, Figure S27 shows the maximum A_2 and $\overline{A}$ values for three different P_m^i and P_m^g combinations. The A_2 value corresponds to the persistence threshold for a given A_1 value. The $\overline{A}$ value can be interpreted as the highest proportion of a given A_1 value that will result in the persistence of the population. For example, in Figure S27(a), we see that with $P_m^i = 0$ and $A_1 = 0.5$ we require $A_2 < 0.194$ for persistence. This corresponds to $\overline{A} < 0.388$.

Travelling wave behaviour for Equation (S8.1) in a parameter regime corresponding to strictly positive $F_A(\overline{C})$ and the strong Allee effect is shown in Figures S28(a)-(c). This parameter regime leads to $A_1 = 0.723$ and $A_2 = 0.2764$, which is below the persistence threshold value of $A_2 = 0.315$ for this P_m^i and P_m^g combination, and hence the population persists.

Sub-case 8.2. **Extinction-degenerate non-negative nonlinear diffusivity function.** For extinction-degenerate $F_A(\overline{C})$, $P_m^i = 0$. As such, the persistence threshold corresponds to $(6A_1^2 - 12A_1)/(9A_1^2 - 20A_1)$. For Case 6.2 we observe that sharp fronts for the strong Allee effect with a extinction-degenerate non-negative $F(C)$ only occur if $v > 0$. Hence for $\overline{A} < (6A_1^2 - 12A_1)/(9A_1^2 - 20A_1)$ Equation (S8.1) should approach a

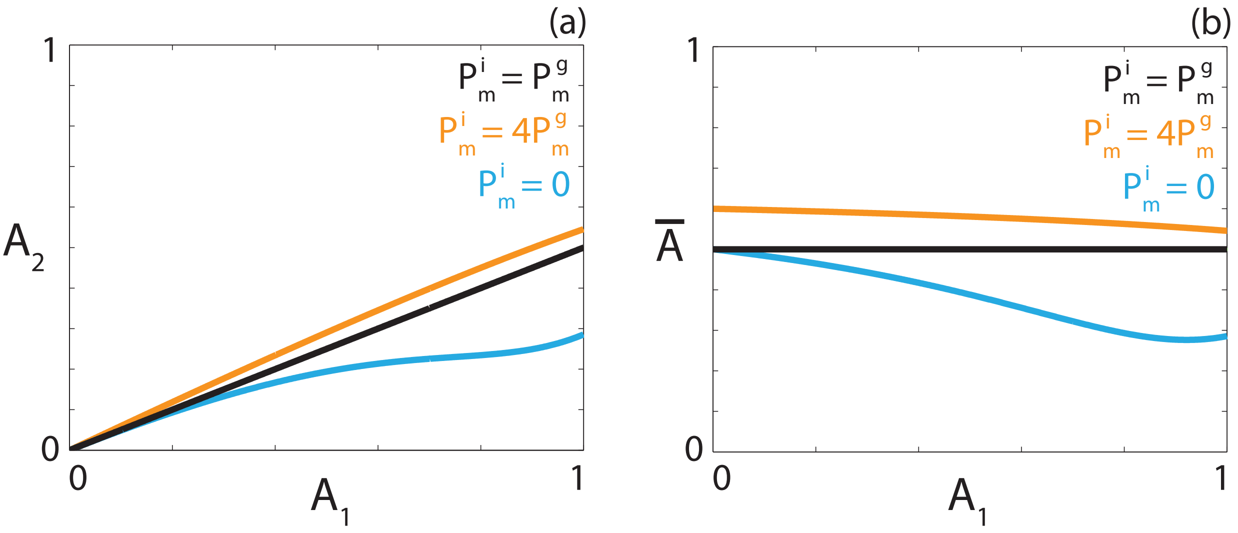

FIGURE S27. **Persistence threshold**. Persistence threshold as a function of the carrying capacity A_1, expressed as (a) an explicit value, and (b) a proportion of the carrying capacity for three different diffusivities, corresponding to $P_m^i = P_m^g$ (black), $P_m^i = 4P_m^g$ (orange) and $P_m^i = 0$ (cyan).

sharp-fronted travelling wave solution with $v > 0$, and a smooth travelling solution with $v < 0$ otherwise. Results in Figures S28(d)-(f) show numerical solutions of Equation (S8.1) with $A_1 = 0.723$ and $A_2 = 0.2764$, which satisfies the threshold for $v > 0$ and hence sharp-fronted travelling wave solutions exist. As expected, results in Figure S28(e) indicate that the solution of Equation (S8.1) approaches a travelling wave with $v > 0$ and a sharp front near $C = 0$.

Sub-case 8.3. **Positive-negative-positive nonlinear diffusivity function.**

For a positive-negative-positive $F_A(\overline{C})$, there are exactly two zeros at $C = \alpha$ and $C = \beta$. In Case 6.3 the strong Allee effect does not give rise to smooth travelling wave solutions, even with real-valued holes in the wall at $C = \alpha$ and $C = \beta$. However, interestingly, shock-fronted travelling wave solutions arise from the Heaviside initial condition. Again, we are unable to find numerical travelling wave solutions of Equation (S8.1) in parameter regimes with real-valued holes in the wall. Shock-fronted travelling wave solutions of Equation (S8.1) are given in Figures S28(g)-(i) where the observed wave speed is $v = 0.014 < 2\sqrt{\min\{F'(C)R(C)\}}$ on the interval $2A_1/3 < C < A_1$. Smooth travelling wave solutions obtained from the weak and reverse Allee effect are shown in Figure S29.

Sub-case 8.4. **Capacity-degenerate positive-negative nonlinear diffusivity function.** Capacity-degenerate positive-negative $F_A(\overline{C})$ requires $P_m^i = 0$ and, subsequently, $F_A(1) = 0$. Furthermore $F_A(\overline{C}) < 0$ for $\omega < C < S$. For Case 6.4 we found smooth travelling wave solutions for both the weak and reverse Allee effect with capacity-degenerate positive-negative $F(C)$ but could not obtain stable solutions for the strong Allee effect. As $F_A(\overline{C})$ is qualitatively similar to the $F(C)$ considered for Case 6.4 similar results are expected here.

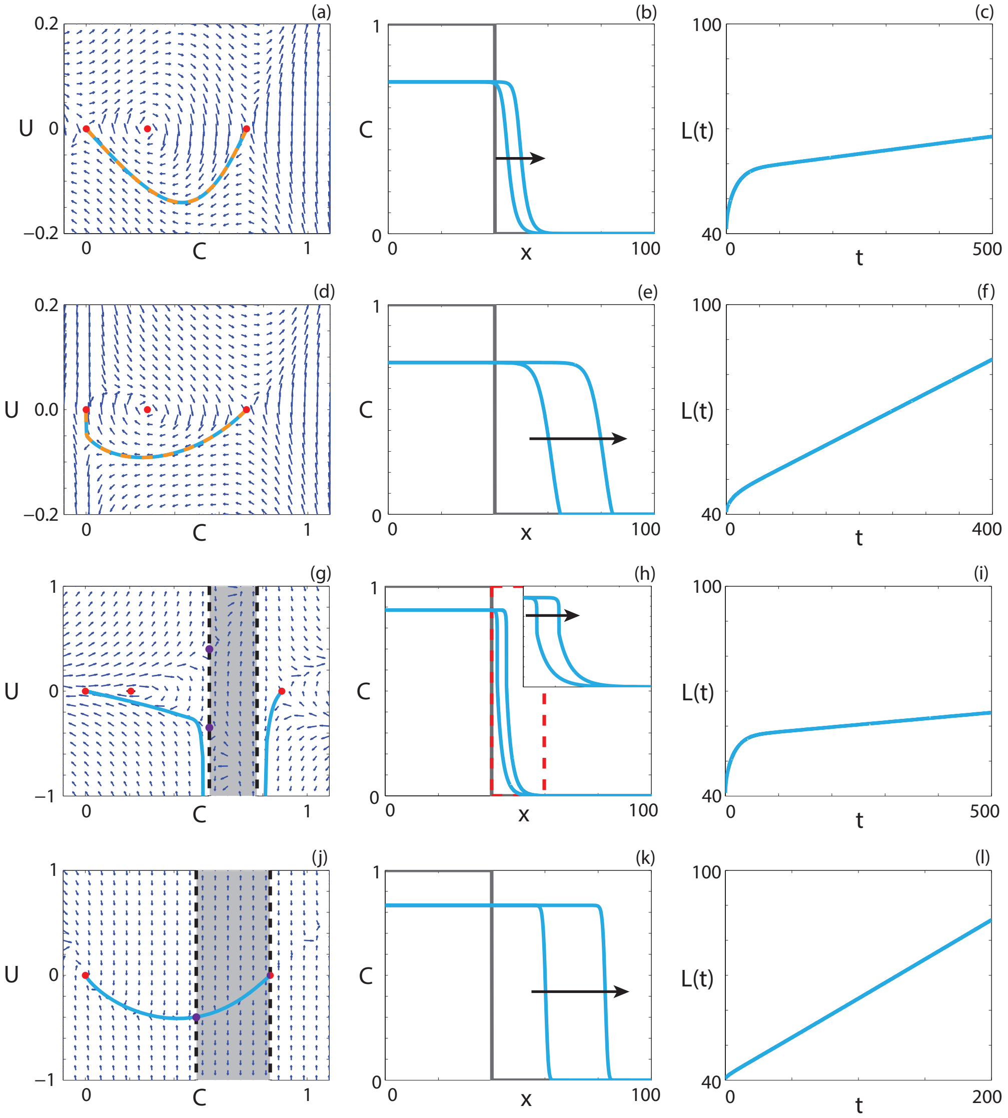

FIGURE S28. **Travelling wave behaviour for Equation** (S8.1) **with various Allee effects and** $F_A(\overline{C})$. We consider the (a)-(c) scaled strong Allee effect with strictly positive $F_A(\overline{C})$ (Case 8.1), (d)-(f) scaled strong Allee effect with extinction-degenerate non-negative $F_A(\overline{C})$ (Case 8.2), (g)-(i) scaled strong Allee effect with positive-negative-positive $F_A(\overline{C})$ (Case 8.3) and, (j)-(l) scaled reverse Allee effect with capacity-degenerate $F_A(\overline{C})$ (Case 8.4). (a), (d), (g), (j) Phase plane for the system (S8.3)-(S8.4) with the numerical solution of Equations (S8.1) (cyan, solid) and (S8.2) (orange, dashed), in (C, U) co-ordinates, superimposed. Red circles correspond to equilibrium points. (b), (e), (h), (k) Numerical solution of Equation (S8.1) calculated at (b) $t = 250$ and $t = 500$; (e) $t = 200$ and $t = 400$; (h) $t = 250$ and $t = 500$; (k) $t = 100$ and $t = 200$. The grey lines indicate the initial condition and the arrows indicate the direction of increasing time. The inset corresponds to the area within the red dashed lines, and highlights the shock. (c), (f), (i), (l) The time evolution of $L(t)$. All results are obtained with $\delta x = 0.05$, $\delta t = 0.001$, $\epsilon = 10^{-6}$, $P_d^g = 0.1$, (a)-(c) $P_m^i = 1.0$, $P_m^g = 0.5$, $P_p^i = 0.7$, $P_p^g = 0.8$, $P_d^i = 0.5$, $v = 0.02$; (d)-(f) $P_m^i = 0$, $P_m^g = 1.0$, $P_p^i = 0.7$, $P_p^g = 0.5$, $P_d^i = 0.8$, $v = 0.098$; (g)-(i) $P_m^i = 1.0$, $P_m^g = 0.23$, $P_p^i = 0.7$, $P_p^g = 0.5$, $P_d^i = 0.8$, $P_d^g = 0.05$, $v = 0.014$; (j)-(l) $P_m^i = 0.05$, $P_m^g = 0.01$, $P_p^i = 0.7$, $P_p^g = 0.5$, $P_d^i = 0.2$, $P_d^g = 6/70$, $v = 0.22$.

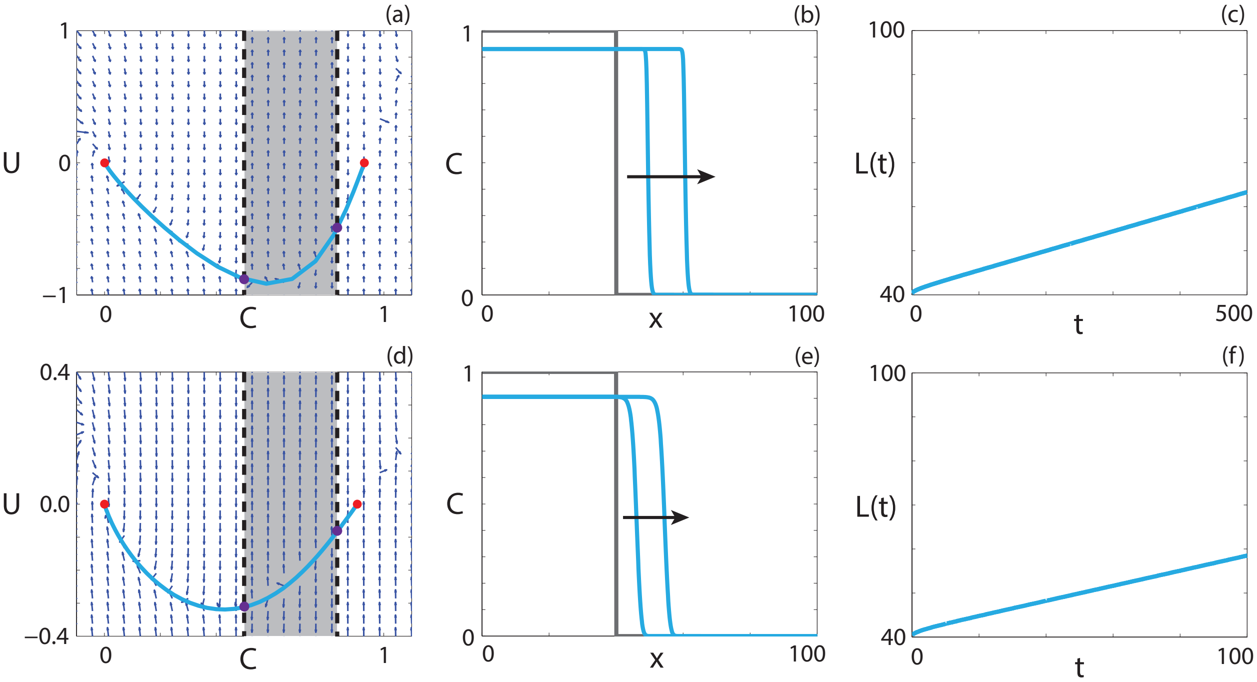

FIGURE S29. **Travelling wave behaviour for Equation** (S8.1) **with the (a)-(c) weak Allee effect and the (d)-(f) reverse Allee effect with positive-negative-positive** $F_s(\overline{C})$ **(Case 8.3)**. (a), (d) Phase plane for the system (S8.3)-(S8.4) with the numerical solution to Equations (S8.1) (cyan, solid) and (S8.2) (orange, dashed), in (C, U) co-ordinates, superimposed. Red circles correspond to equilibrium points. (b), (e) Numerical solution to Equation (S8.1) calculated at (b) $t = 250$ and $t = 500$; (e) $t = 50$ and $t = 100$. The grey lines indicate the initial condition and the arrow indicates the direction of increasing time. (c), (f) The time evolution of the position of the leading edge of the wave front. All results are obtained with $\delta x = 0.1$, $\delta t = 0.01$, $\epsilon = 10^{-6}$, (a)-(c) $P_m^i = 0.01$, $P_m^g = 0.002$, $P_p^i = 0.3$, $P_p^g = 0.4$, $P_d^i = 0.3$, $P_d^g = 0.02$, $v = 0.045$; (d)-(f) $P_m^i = 0.05$, $P_m^g = 0.01$, $P_p^i = 0.6$, $P_p^g = 0.2$, $P_d^i = 0.3$, $P_d^g = 0.02$, $v = 0.172$.

Again, smooth travelling wave solutions of Equation (S8.1) for both the weak (Figures S28(j)-(l)) and reverse (Figure S30) Allee effects are obtained. As for Case 6.4, we consider a variety of parameter regimes corresponding to the strong Allee effect with capacity-degenerate positive-negative $F_A(\overline{C})$, as well as a number of initial conditions, but are unable to find long time travelling wave-type solutions.

Sub-case 8.5. **Positive-negative nonlinear diffusivity function.** For the case where $F_A(\overline{C})$ has exactly one zero on the interval $0 \leq \overline{C} \leq 1$ at $C = \omega$, Maini *et al.* [39] examine the existence of travelling wave solutions, and provide the necessary conditions for existence,

$$A_2 < \omega, \qquad v > 0, \qquad \int_0^\omega F(C)R(C)\,\mathrm{d}C > 0, \tag{S8.9}$$

where $F(\omega) = 0$ and $0 < \omega < 1$. For the strong Allee effect in this parameter regime, the third part of Condition (S8.9) corresponds to

$$\begin{aligned} &D_i(20(A_1 + A_2)\omega - 30A_1A_2\omega - 15\omega^2)+ \\ &(D_i - D_g)((84A_1 + 36A_2)\omega^3 - (45A_1A_2 + 60A_1 + 60A_2)\omega^2 - 30\omega^4 + 80A_1A_2\omega) > 0. \end{aligned} \tag{S8.10}$$

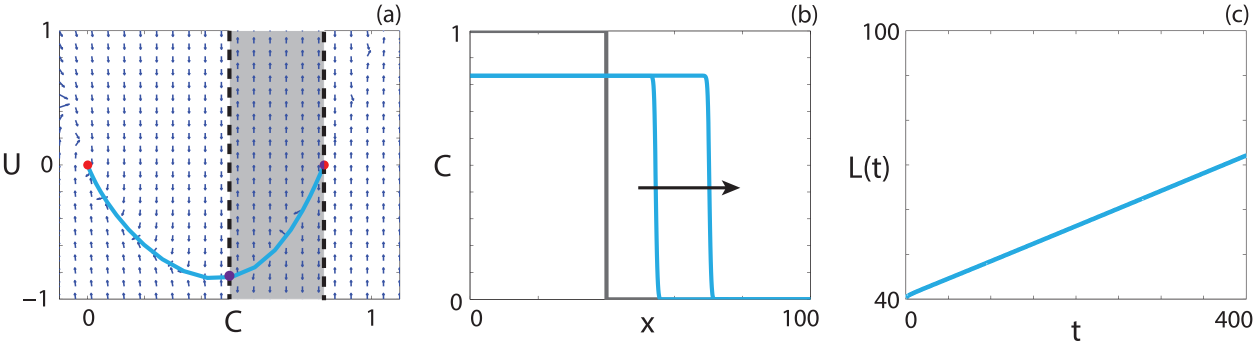

FIGURE S30. **Travelling wave behaviour for Equation** (S8.1) **with the weak Allee effect and positive-negative capacity-degenerate** $F_s(\overline{C})$ **(Case 8.5)**. (a) Phase plane for the system (S8.3)-(S8.4) with the numerical solution to Equations (S8.1) (cyan, solid) and (S8.2) (orange, dashed), in (C, U) co-ordinates, superimposed. Red circles correspond to equilibrium points. (b) Numerical solution to Equation (S8.1) calculated at $t = 200$ and $t = 400$. The grey lines indicate the initial condition and the arrow indicates the direction of increasing time. (c) The time evolution of the position of the leading edge of the wave front. All results are obtained with $\delta x = 0.1$, $\delta t = 0.01$, $\epsilon = 10^{-6}$, $P_m^i = 0.01$, $P_m^g = 0.002$, $P_p^i = 0.6$, $P_p^g = 0.5$, $P_d^i = 0.3$, $P_d^g = 0.08$, $v = 0.079$.

As in Case 4.4, Equation (S8.1) is equivalent to

$$\frac{\partial C}{\partial \hat{t}} = \frac{\partial^2 C}{\partial x^2} + (K_i - K_g - \lambda_i + \lambda_g) A_1 F(C) C \left(1 - \frac{C}{A_1}\right)\left(C - A_2\right), \ \hat{t} \geq 0, \tag{S8.11}$$

on the interval $0 \leq C < \omega$, and equivalent to

$$\frac{\partial C}{\partial \hat{t}} = \frac{\partial^2 C}{\partial x^2} + (K_i - K_g - \lambda_i + \lambda_g) A_1 \hat{F}(C)(1 - C)\left(1 - \frac{1 - C}{A_1}\right)\left(1 - A_2 - C\right), \ \hat{t} \geq 0, \tag{S8.12}$$

where $\hat{F}(C) = -F(1 - C)$, on the interval $\omega < C \leq A_1$. The final necessary and sufficient condition from Maini *et al.* [39] for the existence of travelling wave solutions is that the minimum wave speed for Equation (S8.11), v_1^*, is greater than, or equal to, the minimum wave speed for Equation (S8.12), v_2^*. On the interval $0 \leq C < \omega$, Equation (S8.1) has a strictly positive $F_A(\overline{C})$, where $F_A(\overline{C}) \leq D_i$, and strong Allee kinetics. Hence, the minimum wave speed for Equation (S8.11) has an upper bound, $v_1^* \leq \sqrt{2(\lambda_i - K_i) D_i}(1/2 - A_2)$. On the interval $\omega < C < A_1$ Equation (S8.12) has a source term qualitatively similar to the Fisher-Kolmogorov equation and hence a lower bound for the minimum wave speed exists [39], $v_2^* \geq 2\sqrt{-F(A_1)(\lambda^2 + 4K_g(\lambda_i - \lambda_g - K_i + K_g))^{1/2}}$. For all parameter regimes considered that correspond to the strong Allee effect with positive-negative $F_A(\overline{C})$ we never observe a case where the upper bound for v_1^* is higher than the lower bound for v_2^* and hence the conditions required for travelling wave solutions are not met. As expected, numerical solutions of Equation (S8.1) in these parameter regimes did not lead to travelling wave behaviour. For both the weak and the reverse Allee effect, we expect that solutions do exist as the source terms on both intervals are qualitatively equivalent to a Fisher source term.

Numerical solutions demonstrating the travelling wave behaviour of Equation (S8.1) with positive-negative $F_A(\overline{C})$ and both the reverse Allee effect and the weak Allee effect are given in Figures S31 and S32, respectively.

REFERENCES

[1] Alvord Jr, E. C. & Shaw, C. M. *Neoplasms affecting the nervous system of the elderly* (Oxford University Press, 1991).

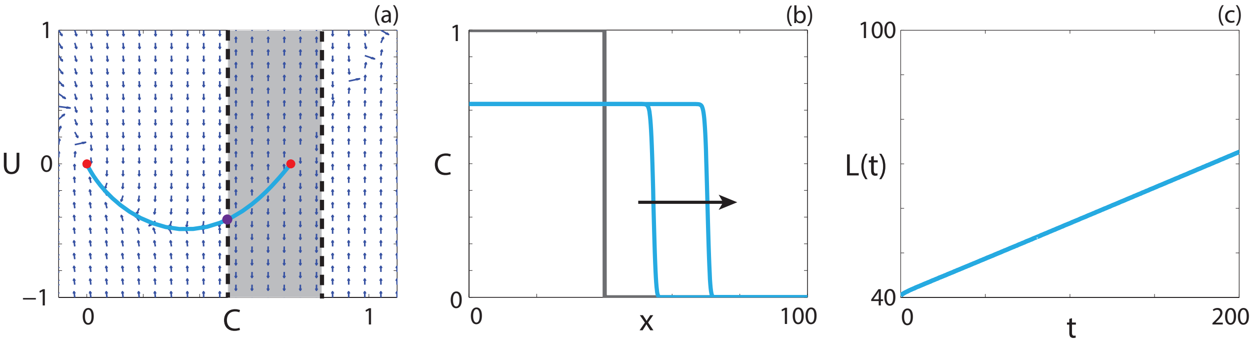

FIGURE S31. **Travelling wave behaviour for Equation** (S8.1) **with the reverse Allee effect and positive-negative** $F_A(\overline{C})$ **(Case 8.5)**. (a) Phase plane for the system (S8.3)-(S8.4) with the numerical solution of Equation (S8.1) (cyan, solid), in (C, U) co-ordinates, superimposed. Red circles correspond to equilibrium points. (b) Numerical solution of Equation (S8.1) calculated at $t = 100$ and $t = 200$. The grey lines indicate the initial condition and the arrows indicate the direction of increasing time. (c), (f) The time evolution of $L(t)$. All results are obtained with $\delta x = 0.1$, $\delta t = 0.01$, $\epsilon = 10^{-6}$, $P_d^g = 0.1$, $P_m^i = 0.025$, $P_m^g = 0.005$, $P_p^i = 0.7$, $P_p^g = 0.5$, $P_d^i = 0.2$, $P_d^g = 0.15$, $v = 0.16$.

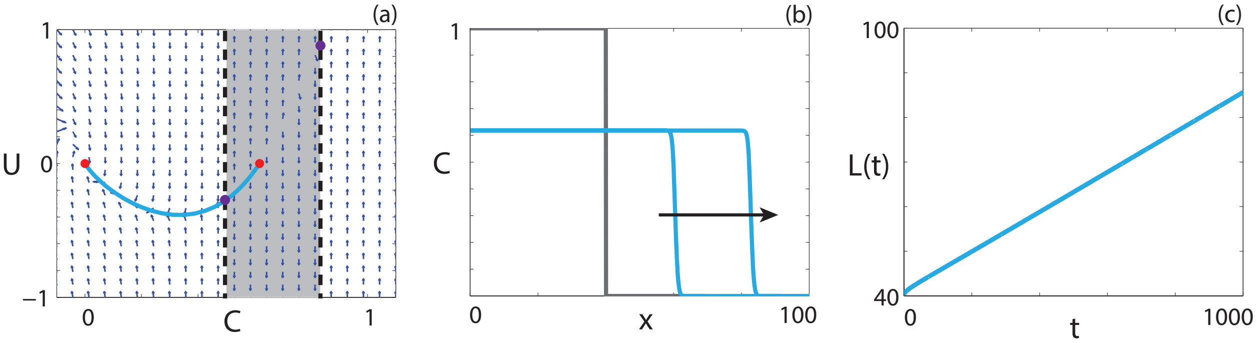

FIGURE S32. **Travelling wave behaviour for Equation** (S8.1) **with the weak Allee effect and positive-negative** $F_s(\overline{C})$ **(Case 8.4)**. (a) Phase plane for the system (S8.3)-(S8.4) with the numerical solution to Equations (S8.1) (cyan, solid) and (S8.2) (orange, dashed), in (C, U) co-ordinates, superimposed. Red circles correspond to equilibrium points. (b) Numerical solution to Equation (S8.1) calculated at $t = 50$ and $t = 100$. The grey lines indicate the initial condition and the arrow indicates the direction of increasing time. (c) The time evolution of the position of the leading edge of the wave front. All results are obtained with $\delta x = 0.1$, $\delta t = 0.01$, $\epsilon = 10^{-6}$, $P_m^i = 0.01$, $P_m^g = 0.002$, $P_p^i = 0.4$, $P_p^g = 0.3$, $P_d^i = 0.3$, $P_d^g = 0.1$, $v = 0.045$.

[2] Clavero, M. & García-Berthou, E. Invasive species are a leading cause of animal extinctions. *Trends in Ecology and Evolution* **20**, 110–110 (2005).

[3] Gabriely, G. *et al.* MicroRNA 21 promotes glioma invasion by targeting matrix metalloproteinase regulators. *Molecular and Cellular Biology* **28**, 5369–5380 (2008).

[4] Gerlee, P. & Nelander, S. The impact of phenotypic switching on glioblastoma growth and invasion. *PLOS Computational Biology* **8**, e1002556 (2012).

[5] Gerlee, P. & Nelander, S. Travelling wave analysis of a mathematical model of glioblastoma growth. *Mathematical Biosciences* **276**, 75–81 (2016).

[6] Johnston, S. T., Simpson, M. J. & McElwain, D. L. S. How much information can be obtained from tracking the position of the leading edge in a scratch assay? *Journal of The Royal Society Interface* **11**, 20140325 (2014).

[7] Martin, P. Wound healing – aiming for perfect skin regeneration. *Science* **276**, 75–81 (1997).

[8] Poujade, M. *et al.* Collective migration of an epithelial monolayer in response to a model wound. *Proceedings of the National Academy of Sciences* **104**, 15988–15993 (2007).
[9] Sherratt, J. A., Lewis, M. A. & Fowler, A. C. Ecological chaos in the wake of invasion. *Proceedings of the National Academy of Sciences* **92**, 2524–2528 (1995).
[10] Swanson, K. R., Bridge, C., Murray, J. D. & Alvord, E. C. Virtual and real brain tumors: using mathematical modeling to quantify glioma growth and invasion. *Journal of the Neurological Sciences* **216**, 1–10 (2003).
[11] Townsend, C. R. Invasion biology and ecological impacts of brown trout *salmo trutta* in New Zealand. *Biological Conservation* **78**, 13–22 (1996).
[12] Axelrod, R., Axelrod, D. E. & Pienta, K. J. Evolution of cooperation among tumor cells. *Proceedings of the National Academy of Sciences* **103**, 13474–13479 (2006).
[13] Courchamp, F., Clutton-Brock, T. & Grenfell, B. Inverse density dependence and the Allee effect. *Trends in Ecology and Evolution* **14**, 405–410 (1999).
[14] Godin, J. G. J. Antipredator function of shoaling in teleost fishes: a selective review. *Le Naturaliste Canadien* **113**, 241–250 (1986).
[15] Taylor, C. M. & Hastings, A. Allee effects in biological invasions. *Ecology Letters* **8**, 895–908 (2005).
[16] Semmens, B. X. *et al.* Quasi-extinction risk and population targets for the Eastern, migratory population of monarch butterflies (danaus plexippus). *Scientific Reports* **6**, 23265 (2016).
[17] Wang, J. *et al.* A framework for the assessment of the spatial and temporal patterns of threatened coastal delphinids. *Scientific Reports* **6**, 19883 (2016).
[18] Ablowitz, M. J. & Zeppetella, A. Explicit solutions of Fisher's equation for a special wave speed. *Bulletin of Mathematical Biology* **41**, 835–840 (1979).
[19] Aronson, D. G. & Weinberger, H. F. Multidimensional nonlinear diffusion arising in population genetics. *Advances in Mathematics* **30**, 33–76 (1978).
[20] Bramson, M. Convergence of solutions of the Kolmogorov equation to travelling waves **44**, 285 (1983).
[21] Canosa, J. On a nonlinear diffusion equation describing population growth. *IBM Journal of Research and Development* **17**, 307–313 (1973).
[22] Fife, P. C. & McLeod, J. B. The approach of solutions of nonlinear diffusion equations to travelling front solutions. *Archive for Rational Mechanics and Analysis* **65**, 335–361 (1977).
[23] Fife, P. C. Long time behavior of solutions of bistable nonlinear diffusion equations. *Archive for Rational Mechanics and Analysis* **70**, 31–36 (1979).
[24] Fisher, R. A. The wave of advance of advantageous genes. *Annals of Eugenics* **7**, 355–369 (1937).
[25] Hadeler, K. P. & Rothe, F. Travelling fronts in nonlinear diffusion equations. *Journal of Mathematical Biology* **2**, 251–263 (1975).
[26] Keitt, T. H., Lewis, M. A. & Holt, R. D. Allee effects, invasion pinning, and species borders. *The American Naturalist* **157**, 203–216 (2001).
[27] Kolmogorov, A. N., Petrovsky, I. G. & Piskunov, N. S. Étude de léquation de la diffusion avec croissance de la quantité de matiere et son application un probleme biologique. *Moscow University Mathematics Bulletin* **1**, 1–25 (1937).
[28] Lewis, M. A. & Kareiva, P. Allee dynamics and the spread of invading organisms. *Theoretical Population Biology* **43**, 141–158 (1993).
[29] Murray, J. D. *Mathematical Biology I: An Introduction* (Springer, New York, 2002).
[30] Rothe, F. Convergence to pushed fronts. *Journal of Mathematics* **11**, 617–634 (1981).
[31] Johnston, S. T., Shah, E. T., Chopin, L. K., McElwain, D. L. S. & Simpson, M. J. Estimating cell diffusivity and cell proliferation rate by interpreting IncuCyte ZOOM assay data using the Fisher-Kolmogorov model. *BMC Systems Biology* **9**, 38 (2015).
[32] Maini, P. K., McElwain, D. L. S. & Leavesley, D. Travelling waves in a wound healing assay. *Applied Mathematics Letters* **17**, 575–580 (2004).
[33] Maini, P. K., McElwain, D. L. S. & Leavesley, D. I. Traveling wave model to interpret a wound-healing cell migration assay for human peritoneal mesothelial cells. *Tissue Engineering* **10**, 475–482 (2004).
[34] Sherratt, J. A. & Murray, J. D. Models of epidermal wound healing. *Proceedings of the Royal Society of London B: Biological Sciences* **241**, 29–36 (1990).
[35] Ito, H. *et al.* Evolution of periodicity in periodical cicadas. *Scientific Reports* **5**, 14094 (2015).
[36] Ferracuti, L., Marcelli, C. & Papalini, F. Travelling waves in some reaction-diffusion-aggregation models. *Advances in Dynamical Systems and Applications* **4**, 19–33 (2009).
[37] Kuzmin, M. & Ruggerini, S. Front propagation in diffusion-aggregation models with bi-stable reaction. *Discrete and Continuous Dynamical Systems: Series B* **16**, 819–833 (2011).
[38] Maini, P. K., Malaguti, L., Marcelli, C. & Matucci, S. Diffusion-aggregation processes with mono-stable reaction terms. *Discrete and Continuous Dynamical Systems: Series B* **6**, 1175–1189 (2006).
[39] Maini, P. K., Malaguti, L., Marcelli, C. & Matucci, S. Aggregative movement and front propagation for bi-stable population models. *Mathematical Models and Methods in Applied Sciences* **17**, 1351–1368 (2007).
[40] Sánchez-Garduño, F. & Maini, P. K. Existence and uniqueness of a sharp travelling wave in degenerate non-linear diffusion Fisher-KPP equations. *Journal of Mathematical Biology* **33**, 163–192 (1994).
[41] Sánchez-Garduño, F. & Maini, P. K. Travelling wave phenomena in non-linear diffusion degenerate Nagumo equations. *Journal of Mathematical Biology* **35**, 713–728 (1997).

[42] Hadeler, K. P. Travelling fronts and free boundary value problems. In *Numerical Treatment of Free Boundary Value Problems*, 90–107 (Springer, 1982).
[43] Hadeler, K. P. Free boundary problems in biological models. *Free boundary problems: Theory and applications* **2**, 664–671 (1983).
[44] Hadeler, K. P. Traveling fronts in parabolic and hyperbolic equations. In *Dynamical Systems*, 154–164 (Springer, 1987).
[45] Malaguti, L. & Marcelli, C. Sharp profiles in degenerate and doubly degenerate Fisher-KPP equations. *Journal of Differential Equations* **195**, 471–496 (2003).
[46] Sherratt, J. A. & Marchant, B. P. Nonsharp travelling wave fronts in the Fisher equation with degenerate nonlinear diffusion. *Applied Mathematics Letters* **9**, 33–38 (1996).
[47] Sherratt, J. A. On the form of smooth-front travelling waves in a reaction-diffusion equation with degenerate nonlinear diffusion. *Mathematical Modelling of Natural Phenomena* **5**, 64–79 (2010).
[48] Padrón, V. Effect of aggregation on population recovery modeled by a forward-backward pseudoparabolic equation. *Transactions of the American Mathematical Society* **356**, 2739–2756 (2004).
[49] Malaguti, L., Marcelli, C. & Matucci, S. Front propagation in bistable reaction-diffusion-advection equations. *Advances in Differential Equations* **9**, 1143–1166 (2004).
[50] Landman, K. A. & White, L. R. Terraced spreading of nanofilms under a nonmonotonic disjoining pressure. *Physics of Fluids* **23**, 012004 (2011).
[51] Witelski, T. P. Shocks in nonlinear diffusion. *Applied Mathematics Letters* **8**, 27–32 (1995).
[52] Pettet, G. J., McElwain, D. L. S. & Norbury, J. Lotka-volterra equations with chemotaxis: Walls, barriers and travelling waves. *Mathematical Medicine and Biology* **17**, 395–413 (2000).
[53] Wechselberger, M. & Pettet, G. J. Folds, canards and shocks in advection–reaction–diffusion models. *Nonlinearity* **23**, 1949 (2010).
[54] Forbes, L. K. & Derrick, W. A combustion wave of permanent form in a compressible gas. *The ANZIAM Journal* **43**, 35–58 (2001).
[55] Korolev, K. S., Xavier, J. B. & Gore, J. Turning ecology and evolution against cancer. *Nature Reviews Cancer* **14**, 371–380 (2014).
[56] Sewalt, L., Harley, K., van Heijster, P. & Balasuriya, S. Influences of Allee effects in the spreading of malignant tumours. *Journal of Theoretical Biology* **394**, 77–92 (2016).
[57] Anguige, K. & Schmeiser, C. A one-dimensional model of cell diffusion and aggregation, incorporating volume filling and cell-to-cell adhesion. *Journal of Mathematical Biology* **58**, 395–427 (2009).
[58] Johnston, S. T., Simpson, M. J. & Baker, R. E. Mean-field descriptions of collective migration with strong adhesion. *Physical Review E* **85**, 051922 (2012).
[59] Keller, E. F. & Segel, L. A. Model for chemotaxis. *Journal of Theoretical Biology* **30**, 225–234 (1971).
[60] Codling, E. A., Plank, M. J. & Benhamou, S. Random walk models in biology. *Journal of the Royal Society Interface* **5**, 813–834 (2008).
[61] Baker, R. E. & Simpson, M. J. Correcting mean-field approximations for birth-death-movement processes. *Physical Review E* **82**, 041905 (2010).
[62] Chowdhury, D., Schadschneider, A. & Nishinari, K. Physics of transport and traffic phenomena in biology: from molecular motors and cells to organisms. *Physics of Life Reviews* **2**, 318–352 (2005).
[63] Simpson, M. J., Landman, K. A. & Hughes, B. D. Cell invasion with proliferation mechanisms motivated by time-lapse data. *Physica A: Statistical Mechanics and its Applications* **389**, 3779–3790 (2010).
[64] Johnston, S. T., Baker, R. E. & Simpson, M. J. Filling the gaps: A robust description of adhesive birth-death-movement processes. *Physical Review E* **93**, 042413 (2016).
[65] Simpson, M. J. & Baker, R. E. Corrected mean-field models for spatially dependent advection-diffusion-reaction phenomena. *Physical Review E* **83**, 051922 (2011).
[66] Johnston, S. T., Simpson, M. J. & Baker, R. E. Modelling the movement of interacting cell populations: a moment dynamics approach. *Journal of Theoretical Biology* **370**, 81–92 (2015).
[67] Hughes, B. D. *Random Walks and Random Environments*, vol. 1 (Clarendon Press; Oxford University Press, 1995).
[68] Press, W. H. *Numerical Recipes 3rd Edition: The Art of Scientific Computing* (Cambridge University Press, 2007).
[69] Shampine, L. F. & Reichelt, M. W. The Matlab ODE suite. *SIAM Journal on Scientific Computing* **18**, 1–22 (1997).
[70] Briscoe, B. K. & Lewis, M. A. & Parrish, S. E. Home range formation in wolves due to scent marking. *Bulletin of Mathematical Biology* **64**, 261–284 (2002).
[71] Turchin, P. Population consequences of aggregative movement. *Journal of Animal Ecology* **58**, 75–100 (1989).
[72] Lews, M. A. Spatial coupling of plant and herbivore dynamics: the contribution of herbivore dispersal to transient and persistent “waves” of damage. *Theoretical Population Biology* **45**, 277–312 (1994).
